# A scoping review of published literature on chikungunya virus

**DOI:** 10.1371/journal.pone.0207554

**Published:** 2018-11-29

**Authors:** Mariola Mascarenhas, Sophiya Garasia, Philippe Berthiaume, Tricia Corrin, Judy Greig, Victoria Ng, Ian Young, Lisa Waddell

**Affiliations:** 1 National Microbiology Laboratory at Guelph, Public Health Agency of Canada, Guelph, Ontario, Canada; 2 National Microbiology Laboratory at St. Hyacinthe, Public Health Agency of Canada, St. Hyacinthe, Quebec, Canada; 3 School of Occupational and Public Health, Ryerson University, Toronto, Ontario, Canada; Faculty of Science, Ain Shams University (ASU), EGYPT

## Abstract

Chikungunya virus (CHIKV) has caused several major epidemics globally over the last two decades and is quickly expanding into new areas. Although this mosquito-borne disease is self-limiting and is not associated with high mortality, it can lead to severe, chronic and disabling arthritis, thereby posing a heavy burden to healthcare systems. The two main vectors for CHIKV are *Aedes aegypti* and *Aedes albopictus* (Asian tiger mosquito)*;* however, many other mosquito species have been described as competent CHIKV vectors in scientific literature. With climate change, globalization and unfettered urban planning affecting many areas, CHIKV poses a significant public health risk to many countries. A scoping review was conducted to collate and categorize all pertinent information gleaned from published scientific literature on *a priori* defined aspects of CHIKV and its competent vectors. After developing a sensitive and specific search algorithm for the research question, seven databases were searched and data was extracted from 1920 relevant articles. Results show that CHIKV research is reported predominantly in areas after major epidemics have occurred. There has been an upsurge in CHIKV publications since 2011, especially after first reports of CHIKV emergence in the Americas. A list of hosts and vectors that could potentially be involved in the sylvatic and urban transmission cycles of CHIKV has been compiled in this scoping review. In addition, a repository of CHIKV mutations associated with evolutionary fitness and adaptation has been created by compiling and characterizing these genetic variants as reported in scientific literature.

## Introduction

Chikungunya disease is caused by chikungunya virus (CHIKV), a mosquito-borne alphavirus belonging to the family *Togaviridae*. The virus was first isolated from a patient in Tanzania during an outbreak of a dengue-like illness that had been circulating in that area between 1952 and 1953. Frequent descriptions of this illness had been reported in earlier years in other parts of Africa, including Mozambique [1]. The virus got its name from a word in the local dialect of the Makonde area in Tanzania and is literally translated as “bent over in pain” [2], describing the stooped posture of many chikungunya afflicted patients due to severe joint pains and arthralgias, both hallmark features of the disease. The proportion of asymptomatic CHIKV infections during epidemics has been reported to be between 3 to 28% [3–5]. Together with severe joint pain, patients typically exhibit febrile exanthema with a saddleback fever pattern, symptoms also seen with Dengue virus (DENV) and Zika virus (ZIKV) infections [6]. This makes CHIKV diagnosis difficult, particularly since there are many reports of co-circulating CHIKV, DENV and ZIKV in many areas with co-infected patients [7–14].

CHIKV is an enveloped, positive strand RNA virus with a genome usually a little less than 12000 nucleotides. Its genome consists of five structural proteins; E1, E2, E3, C (Capsid protein), 6K, and four non-structural proteins; non-structural proteins 1 (nsP1), 2 (nsP2), 3 (nsP3) and 4 (nsP4) [15–16]. Evolutionary studies on the virus provide evidence that the West African lineage of CHIKV originated in Africa and subsequently moved into Asia where it evolved into a distinct Asian lineage. The CHIKV isolate from the epidemic in Reunion Island in 2005 showed that the virus evolved into the East/Central/South African (ECSA) lineage with more recent Indian Ocean strains forming an Indian Ocean sub-lineage within ECSA [17–20]. The large chikungunya epidemic in Brazil that started in late 2013 was reported to be the outcome of two separate introductions of two individual CHIKV lineages; Asian and ECSA [21–23].

CHIKV is maintained in a sylvatic cycle involving wild primates and arboreal mosquitoes in Africa, and a mosquito to (and from) human urban cycle in countries with human epidemics [24]. The urban cycle of CHIKV has been responsible for several large CHIKV epidemics in countries spanning many continents. During the 2005–2006 epidemics in the Indian Ocean Islands and India, more than a third of the population in Reunion Island were infected with CHIKV (34.3%) resulting in many severe clinical cases, while case-fatality rates of 4.5% and 4.9% were reported in Mauritius and India respectively [25–26]. There have been sweeping epidemics throughout Asia for a number of years following the Indian Ocean Islands epidemics [27–29]. In late 2013, CHIKV emerged in the Caribbean for the first time and caused an epidemic in St. Martin, followed by its expansion into other islands in close proximity [30–33]. Around the same time, CHIKV also spread across South and Central America causing major epidemics throughout the region where competent vectors were already established.

The two main competent vectors of CHIKV are considered to be *Aedes aegypti* and *Aedes albopictus*, although the virus has been isolated from other mosquitoes [34–36]. *Ae*. *aegypti* mosquitoes thrive in tropical and arid areas but cannot withstand cool temperatures. These mosquitoes were responsible for all the recorded CHIKV epidemics until the Indian Ocean Islands epidemic in 2005–2006 when a point mutation occurred in the outer membrane E1 glycoprotein of CHIKV, causing an amino acid change with an Alanine to Valine substitution at position 226 [37]. This variant offered *Ae*. *albopictus* the propensity to emerge as a highly competent CHIKV vector with enhanced transmission capability [37–38]. *Ae*. *albopictus* mosquitoes are highly invasive and have successfully established populations in temperate climates [39–41]. With climate change, there is a strong concern that these vectors will expand into new areas.

A scoping review (ScR) on CHIKV and its competent vectors was conducted to identify, characterize, categorize, and elucidate global evidence from published research on this topic. The product of this ScR is a comprehensive report of the evidence and knowledge gaps that exist on this important emerging public health issue. A ScR utilizes reproducible, systematic and rigorous knowledge synthesis methodology to identify and summarize the extent and range of knowledge on a particular topic or issue [42–44]. Results generated from this ScR can inform policy-makers, as well as the scientific community, and facilitate decision-making on mitigation of chikungunya and/or CHIKV competent vectors.

## Methods

A ScR protocol was created *a priori* to ensure transparency, reproducibility and consistency during all stages of our review; S1 Protocol. We followed the general framework outlined by Arksey and O’Malley [42] which included: 1) establishing a research question, 2) conducting a stringent literature search, 3) relevance screening of captured articles, 4) characterizing all relevant articles, and 5) collating and summarizing results from extracted data. This ScR was designed and executed to be reproducible by employing good synthesis research practices, which included a complete search strategy. Screening and data characterization tools were developed *a priori* and uniformly implemented on all citations and articles included in the review by two independent reviewers [42–44]. The reporting of this ScR is aligned with the Preferred Reporting Items for Systematic Reviews and Meta-Analyses (PRISMA) guidelines [45].

### Expert consultation

Prior to finalizing the research question, we solicited input from an expert advisory team from Canada and South America consisting of four government and two academic scientists with expertise on the topic. Our topic experts consented to help us frame the scope of this study by answering a questionnaire in writing; S1 Expert Questionnaire. All but one expert also participated in a teleconference call aimed to discuss key issues and information needs required for public health professionals to assess and address this emerging issue. Subsequent discussions were held with the experts whenever clarification was needed. The finalized protocol was shared with our topic experts before commencing our review.

### Research question

This review had two main research questions:

“What is the current state of research knowledge on chikungunya virus (CHIKV), including its pathogenesis, attributes of clinical infection and diagnosis, surveillance and epidemiology, economic burden, prevention and control strategies, risk factors for developing CHIKV infection, societal attitudes and perceptions regarding CHIKV infection, in humans and other vertebrate hosts**.”**

**And**,

“What vectors are competent for transmission of CHIKV, what are the parameters for effective CHIKV transmission and vector competence, what ecological risk factors contribute to CHIKV competent vector abundance, and what vector mitigation strategies can prevent and control CHIKV vectors?”

#### Search strategy

A comprehensive and stringent search algorithm was developed and pretested to capture relevant literature. The final search algorithm included two parts, one encompassing chikungunya research and the other to capture additional research on CHIKV vectors:

(Chikungunya OR CHIK OR CHIKV)

OR

(alphavirus AND mosquito* AND control)

The search was conducted on May 27, 2015 and updated on January 6, 2017 in the following databases: Scopus, PubMed, Cumulative Index to Nursing and Allied Health Literature (CINAHL), Centre for Agriculture and Biosciences International (CABI), Literatura Latino Americana em Ciências da Saúde (LILACS), Agricola and Cochrane library. The update was conducted to include more recent studies from Central and South America due to the recent emergence of chikungunya in those regions. To verify that our search algorithm had an optimal degree of sensitivity to capture all relevant articles pertaining to our research question, we hand-searched reference lists from 10 relevant narrative reviews for missed citations [46–55]. The grey literature search was conducted in October 2015 and included targeted, iterative hand searching of 22 government and/or research organization websites that were suggested during the expert consultation and are listed in S1 Protocol. Twenty two additional citations were added to the review from the grey literature search.

#### Inclusion/Exclusion criteria

Articles included in this ScR reported primary data that were generated by the authors, or through reliable government and/or research organizations. Included articles had to be relevant to one or more aspects of the review question: i.e., information on CHIKV and chikungunya disease, competence parameters of CHIKV vectors, or control of CHIKV vectors. Articles in languages other than English, French, Spanish or Portuguese were excluded due to limited translation resources. Documents without primary data, such as whole conference proceedings, books, literature reviews, systematic reviews, meta-analyses and some types of grey literature were also excluded from the review.

#### Relevance screening and data characterization

Relevance screening was conducted in two stages. The first screening tool comprised of two questions to determine whether the citation could potentially be relevant to the review based on the title and abstract. We erred on the side of inclusiveness by including citations whenever their relevance remained unclear. Full-text articles of citations that passed through the first relevance screening stage were procured and assigned to a second level of relevance screening. The purpose of this step was to exclude articles after full text assessment, so that only relevant articles in full alignment with the research question remained for further characterization. At the second level of relevance screening, articles were excluded after full text assessment if they did not report on: 1) the impact of CHIKV on humans, hosts or vectors, 2) CHIKV studies including phylogeny and molecular characterization of genetic determinants for fitness and other evolutionary markers, CHIKV pathogenesis and infection mechanisms, 3) mitigation strategies to prevent and control CHIKV infections including strategies for CHIKV vector prevention and control, 4) clinical aspects of CHIKV infection and risk factors contributing to such infections, and 5) vector competence parameters—both behavioural and physiologic, as well as environmental factors associated with CHIKV transmission, 6) economic burden of chikungunya disease, or 7) modeling studies on chikungunya or CHIKV vectors. Articles that did not meet the inclusion criteria for language and primary data were also excluded at this stage. For articles that met the inclusion criteria, questions were answered at this level to broadly categorize articles by publication period, study location, study population (stratified as human and non-human hosts, vectors and/or virus) and research focus. This stage in the ScR process allowed us to gauge the quantity and breadth of research within the different focus areas and ensure that the data characterization and utility (DCU) tool would adequately capture relevant data from all studies.

The third stage of the ScR was data characterization of all relevant articles using the DCU tool; S1 Protocol. Questions included general information on when and where the research was conducted, the study population and genotype of CHIKV. The research focus area(s) was selected at this step and reviewers could access a unique set of questions tailored to each focus area, which aided in efficient characterization of the research. *In vitro* studies examining pathogenesis and/or immunomodulatory mechanisms associated with CHIKV infection in hosts and vectors were identified at the second stage of screening; however, due to the large number of articles on this single focus area, these studies were not characterized in detail at the third stage of the ScR as this was logistically unfeasible for completion within a reasonable timeframe.

#### Review management

All citations captured using the search algorithm were imported from the bibliographic databases to RefWorks (ProQuest, LLC), an online citation management program. The citation database was de-duplicated so that only unique citations remained. The citations were then imported into an online systematic review software, Distiller SR (Evidence Partners, Ottawa, Canada), which is specifically designed to manage all aspects of scoping reviews. All stages of the ScR were conducted by two reviewers working independently using the *a priori* developed data collection tools within Distiller SR. All forms were pre-tested by all reviewers to confirm reproducibility, clarity and consistency between reviewers on each form. Reviewers pre-tested the Relevance Screening Level 1 (RS1) form with 36 citations, the Relevance Screening Level 2 (RS2) form was pre-tested with ten articles and the Data Extraction and Utility (DCU) form was pre-tested on ten articles representing all the main focus areas of the review question, (kappa >0.8); S1 Protocol. After pre-testing, any necessary adjustments were made to the forms to improve clarity and consistency between reviewers. Any discrepancies on extracted data between reviewers were resolved by consensus, or with the help of a third reviewer. The completed dataset was exported into Microsoft Excel for data cleaning, categorization, descriptive analysis and charting.

## Results

**Fig 1:**
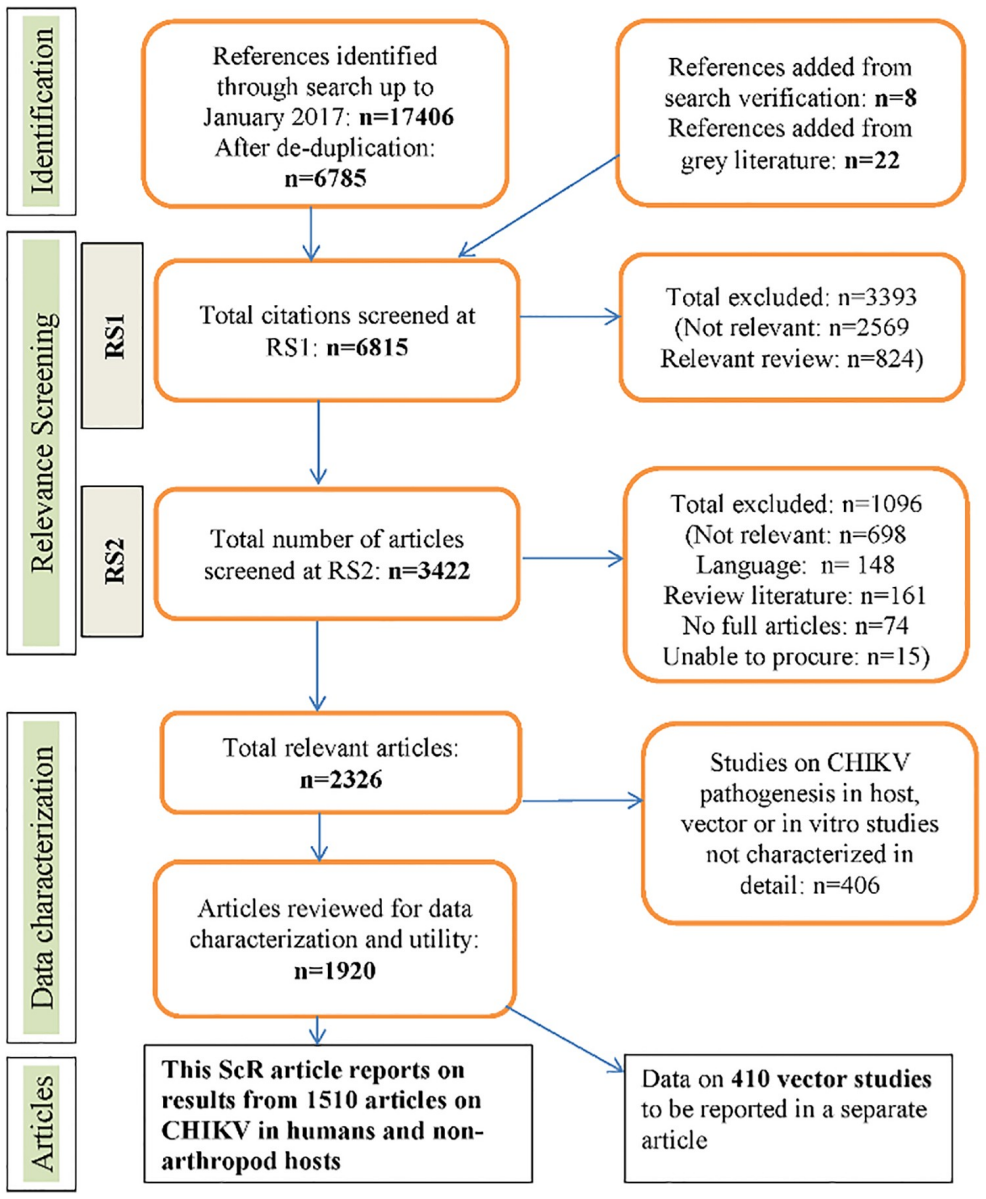
PRISMA chart of the flow of articles through the ScR. See S1 Checklist.

From 6815 unique citations captured by the search, 3422 potentially relevant articles were procured and 2326 were considered primary research relevant to the research question (Fig 1). Four percent (148/3422) of potentially relevant articles were excluded from the review on the basis of language: 57 Chinese, 23 German, 20 Russian, 14 Japanese, 9 Italian, 6 Dutch, 4 Croatian, 3 Danish, 3 Norwegian, 2 Czech, 1 Romanian, 1 Polish, 1 Turkish, 1 Malay, 1 Korean, 1 Thai, and 1 Albanian. We were unable to procure 15 articles despite contacting the authors. Additionally, 74 citations were excluded that were either correspondence or abstracts referencing articles that we were unable to procure or review.

The focus area researched most extensively was epidemiology of chikungunya related to hosts and vectors, with a total of 912/2326 (39%) studies. A little over one third of the articles (764/2326; 33%) reported on signs, symptoms and diagnosis of CHIKV in humans. Treatment for chikungunya in humans was reported in 283/2326 (12%) studies, and mitigation strategies against CHIKV in both humans and vectors comprised 323/2326 (14%) studies. There were 410/2326 (18%) vector studies with relevance to vector competence for transmitting CHIKV and vector behavioural characteristics, while 256/2326 (11%) studies examined phylogeny and signature evolutionary mutations of CHIKV. Also included were 208/2326 (9%) studies describing surveillance programs for CHIKV in humans, other hosts and vectors. The accuracy of CHIKV diagnostic tests was evaluated in 167/2326 (7%) studies. Research areas with fewer studies included modeling studies with relevance to CHIKV and/or its vectors; 103/2326 (4%), social impact examining knowledge, attitudes and perceptions of chikungunya in various target human populations in 46/2326 (2%) studies, and 23/2326 (1%) studies on the economic burden of chikungunya. There were 406/2326 (17%) pathogenesis studies on pathways or mechanisms at the cellular level and detailed study characterization (DCU) was not conducted on these studies. Many studies reported on multiple focus areas. Table 1 is a heat chart of the number of studies in each major and minor focus area by study population category and is referenced throughout the manuscript to aid the reader in tracking the quantity of research underpinning different areas of chikungunya research. This ScR focuses on the detailed characteristics of the 1510 /2326 (65%) articles on vertebrate hosts for CHIKV, while only high level category information is presented for 406/2326 (17%) articles on pathogenesis and immunity related pathways of CHIKV infection. Results from CHIKV vector studies (410/2326; 18%) on important competence and behavioural vector characteristics will be summarized in a subsequent article.

**Table 1:** Heat Chart of study numbers captured by focus area (multiple focus areas in some studies; article numbers associated with colour density).

EVIDENCE MAPPING (n = 2326 studies)							
Number of Studies		Humans (n = 1159)	Non-human primates (NHPs) (n = 51)	Other animals (n = 147)	Vectors: Mosquitoes (n = 657), Non-mosquito arthropods (n = 7)	Virus (n = 256)	*In vitro* and *in silico* studies (n = 158)
**Accuracy of diagnostic tests (n = 167; 7%)**	**Tests assessed for accuracy**	127	1	9	16	−	21
	Molecular tests	70	−	−	7	−	11
	Serology/hemolymph tests	75	−	7	6	−	8
	Virus isolation and identification	9	1	2	3	−	2
	Clinical signs and symptoms algorithm	16	−	−	−	−	−
**Epidemiology (n = 912; 39%)**	**Epidemiology**	848	24	37	134	−	−
	Burden of CHIKV	562	17	34	73	−	−
	Risk factors	70	−	−	128	−	−
	Epidemic investigations	141	1	3	47	−	−
	Travel related CHIKV	215	−	−	16	−	−
	**Co-infection**	109	−	−	2	−	−
	DENV+CHIKV	77	−	−	1	−	−
	ZIKV+CHIKV	9	−	−	1	−	−
	DENV+CHIKV+ZIKV	4	−	−	−	−	−
	**Co-morbidity**	74	−	−	−	−	−
	Diabetes	39	−	−	−	−	−
	Cardiovascular disease	42	−	−	−	−	−
	**Transmission**	54	11	6	50	−	−
	Sylvatic	−	11	5	2	−	−
	Congenital/Vertical	49	−	−	18	−	−
	Sexual/Venereal	−	−	−	1	−	−
	Blood products	2	−	−	−	−	−
**Surveillance (n = 208; 9%)**	**Surveillance**	126	4	3	122	−	−
	Epidemic	46	−	−	19	−	−
	Non-epidemic	117	4	3	105	−	−
	Travel related	36	−	−	−	−	−
	Blood donor related	2	−	−	−	−	−
**Signs, Symptoms of CHIKV infection (n = 764; 33%) and CHIKV diagnostics (n = 687; 30%)**	**Diagnosis**	687	−	−	−	−	−
	Molecular tests	345	−	−	89	−	−
	Serology/hemolymph tests	574	−	−	57	−	−
	Virus culture and identification (includes suckling mouse tests)	117	−	−	35	−	−
	Clinical signs and symptoms	126	−	−	−	−	−
	**Atypical signs nad symptoms**	121	−	−	−	−	−
	Hyperpigmentation	38	−	−	−	−	−
	Ocular	15	−	−	−	−	−
	Neurological	85	−	−	−	−	−
	**Sequelae**	239	−	−	−	−	−
	Arthritis	158	−	−	−	−	−
	Guillain Barré Syndrome	9	−	−	−	−	−
	Other neurological	27	−	−	−	−	−
	Ocular	18	−	−	−	−	−
**Treatment (n = 283; 12%)**	**Prescribed**	172	−	−	−	−	−
	**Assessed for efficacy**	74	−	−	−	−	−
	Non-steroidal anti-inflammatrories (NSAIDs)	17	−	−	−	−	−
	Steroids	19	−	−	−	−	−
	Analgesics	4	−	−	−	−	−
	Antivirals	17	−	−	−	−	17
	Antimalarials	12	−	−	−	−	−
	Traditional medicine	19	−	−	−	−	−
**Knowledge, Attitudes and Perception (n = 46; 2%)**	Concerns about toxicity of insecticides	3	−	−	−	−	−
	Perceptions about disease severity	16	−	−	−	−	−
	Perceived efficacy of protection measures	13	−	−	−	−	−
	Knowledge on behavioural mitigation practices	33	−	−	−	−	−
	Knowledge on chikungunya disease	29	−	−	−	−	−
	Knowledge on CHIKV vectors	27	−	−	−	−	−
	Attitudes towards paying for protection against vectors	1	−	−	−	−	−
**Mitigation (n = 323; 14%)**	Vaccination	8	7	42	−	−	21
	Behavioral protection measures	79	−	−	−	−	−
	Use of insecticides	−	−	−	156	−	−
	Sterile Insect Technique (SIT)	−	−	−	7	−	−
	Incompatible Insect Technique (IIT)	−	−	−	14	−	−
	Release of Insects with Dominant Lethal (RIDL) Mosquitoes	−	−	−	2	−	−
	Bacterial infection of mosquitoes	−	−	−	23	−	−
	Use of larvivores	−	−	−	7	−	−
	Public education	48	−	−	−	−	−
**Economic Burden (n = 23; 1%)**	Economic burden	23	−	−	−	−	−
	Cost-benefit of control measures	4	−	−	−	−	−
**Modeling (n-103; 4%)**	Risk assessment	44	−	−	−	−	−
	Economic models	4	−	−	−	−	−
	Disease transmission	51	−	−	−	−	−
	Vector mapping	−	−	−	23	−	−
**Virus studies (n = 256; 11%)**	Phylogeny	−	−	−	−	118	−
	Molecular characterization	−	−	−	−	155	−
**Pathogenicity or Infection mechanisms (n = 406; 17%)**	**Data not extracted**						

### Publications by continent and time

**Fig 2:**
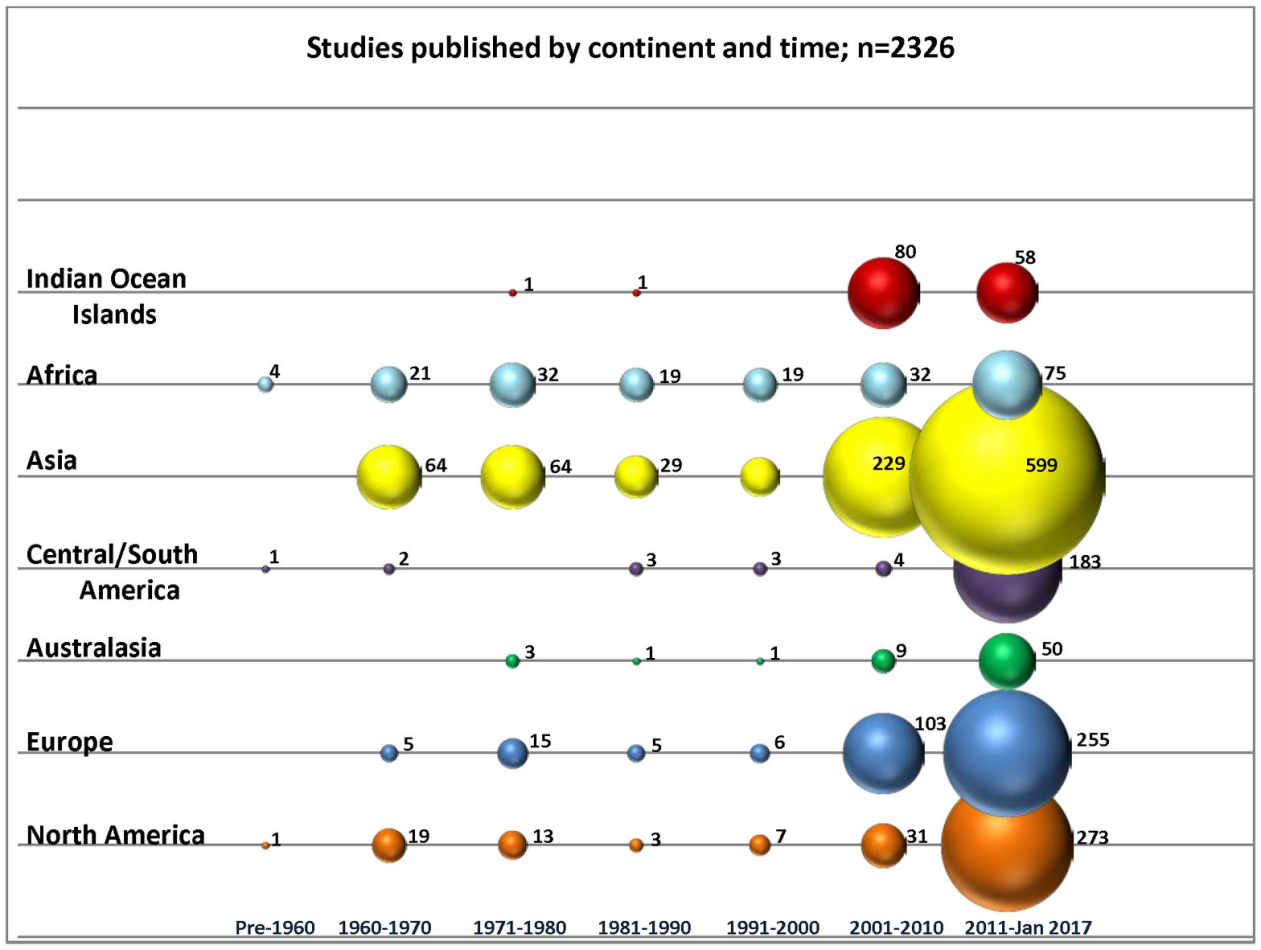
Publications (n = 2326) on CHIKV or its competent vectors by decade of publication and continent of research. Study numbers for each category represented by bubble size and reported with corresponding bubbles.

Generally, interest in CHIKV research increased in regions soon after they were affected by epidemics (Fig 2). The majority of research has been conducted since 2000 across Asia, North America, Europe and the Indian Ocean Islands. Many of the articles that reported on the major CHIKV epidemic in Reunion Island and other nearby Indian Ocean Islands during 2005–2006 were published by authors in both France and the affected islands. There were major CHIKV epidemics reported in India and Southeast Asia from 2006 onwards and multiple articles have described various aspects of these epidemics. Autochthonous transmission of CHIKV was reported in Italy [56]; and France [57] between 2007 and 2010 and many studies reported on these locally transmitted outbreaks. Since the emergence of CHIKV in the Americas, there have been several reports on autochthonous CHIKV transmission in newly affected areas [58–59].

### Chikungunya virus

Of the 1920 studies fully characterized in this ScR, lineage of the CHIKV strains isolated or used in experiments was reported in 324 articles; West African lineage (62/324), ECSA lineage (176/324) and Asian lineage (106/324). The Indian Ocean sub-lineage of ECSA was reported in 69/176 (39%) articles and two articles reported a new Caribbean clade [60–61]. Many gene mutations that contribute to evolutionary adaptation and fitness of CHIKV have been reported in scientific literature [62–65]. A repository of these mutations has been created from results gathered in this ScR; S1 Table.

#### Diagnostic testing for CHIKV

Data were captured from three types of testing for CHIKV, predominantly in humans (687/2326; 30%), to: 1) diagnose active CHIKV infection in patients, 2) screen sample populations of hosts for antibodies to CHIKV to detect prior exposure and 3) studies related to the evaluation of the performance of diagnostic tests; Table 1. Results show that 126/687 (18%) studies reported the use of clinical signs and symptoms alone for diagnosis, mostly in resource limited countries with large ongoing epidemics. CHIKV diagnosis in humans reported in the earliest research was conducted by using a suckling mouse animal model to grow and test for the virus, but antigenic tests were soon developed. More recently, molecular tests have been developed and utilized to detect viral RNA, some offering results with a quick turnaround time. Details on the number of studies reporting on each category of CHIKV tests are available in S2 Table.

The vast majority of studies reported CHIKV testing on blood or cerebrospinal fluid (CSF); S2 Table. Uncommon samples that were successfully tested for CHIKV included corneas [66], oral secretions [67–69], peritoneal, gastric and blister fluids [70–73], urine and semen [74]. Saliva was found to be useful in molecular testing only for a week post symptom onset and was reported to be less sensitive than blood samples in one study [69]. Another study reported identifying viable virus in urine and semen samples even up to 30 days post infection [74].

There were 167/2326 (7%) studies on determining the accuracy of CHIKV tests by comparing the sensitivity and specificity of one or more tests; Table 1. Evaluating various diagnostic algorithms based on clinical symptoms to reach an accurate clinical diagnosis was conducted in 16 /167 (10%) studies to help with rapid diagnosis, mainly in regions where laboratory testing is not easily accessible or feasible. Diagnostic accuracy studies for CHIKV included 15/167 (9%) articles assessing virus isolation and culture tests, 90/167 (54%) evaluating serological tests, and 88/167 (53%) on molecular tests; S3 Table.

#### CHIKV in non-human vertebrate hosts

Chikungunya infection was studied in non-human primates (NHPs) (51/1920; 3% studies), other vertebrates such as rodents (12/1920; 1% studies), reptiles (4/1920; 0.2% studies), bats (6/1920; 0.3% studies), wild birds (12/1920, 0.6% studies), farm animals (33/1920; 2% studies) and domestic pets (8/1920; 0.4% studies); Table 1. Surveillance for CHIKV in animals was identified in 7/208 (3%) surveillance studies; Table 1. All non- human vertebrate surveillance studies were conducted during non-epidemic periods and investigated exposure to CHIKV infection.

#### Epidemiological studies on potential sylvatic hosts for CHIKV

The potential contribution of non-human vertebrate hosts towards sylvatic transmission of CHIKV was investigated in 12 observational studies (Table 1) with 11/12 (92%) articles on NHPs, 3/12 (25%) on domestic and farm animals, 1/12 (8%) on wild birds, and 4/12 (33%) on non-primate wild animals and reptiles. Sylvatic transmission has only been reported in Africa where early studies implicated primates, rodents and bats as possible sylvatic hosts; Table 2. Outside Africa, a few surveys have provided some evidence for a number of monkey species, small rodents and bats to possibly play a role as sylvatic hosts for CHIKV; Table 2.

**Table 2:** Results from observational studies evaluating potential vertebrate sylvatic hosts for CHIKV*.

Sampling date	Country	Positive results**	Negative results	Reference
1962–1963	Zimbabwe	Vervet monkey(*Cercopithecus aethiops)* and baboon (*Papio ursinus)*		[75]
1962–1967	Senegal	Vervet monkey(*Cercopithecus aethiops)*, African ground squirrel (*Xerus erythropus*), Senegal bushbaby (*Galago senegalensis***),** birds and bats (unspecified)		[76]
1968	Uganda	Redtail monkeys (*Cercopithecus ascanius*)	Vervet monkeys (*Cercopithecus aethiops)*, rodents of 10 species (unspecified), birds of many spp. (unspecified), dairy cattle (unspecified), civet cat (*Civettictis civetta*)	[77]
1964–1971	Southern Africa	Monkeys *(Cercopithecus aethiops*), baboons (*Papio ursinus*)		[78]
1976	South Africa	Baboons (*Papio ursinus*)		[79]
1992–1993	Central African Republic		Zebu cattle	[80]
1970s and 1980s	Senegal	Vervet monkey *(Cercopithecus aethiops)*, baboon (*Papio papio)*, Patas monkey *(Erythrocebus patas)*, Senegal bushbaby (*Galago senegalensis galago*), African ground squirrel (*Xerus erythropus)*, palm squirrel (*Funambulus palmarum*), and bats (*Scotophillus and chiroptera sp*.), rodents and birds (unspecified)		[81]
1996–1997	Malaysia		Wild and semi-captive orangutans from Borneo *(Pongo pygmaeus)*	[82]
1991–2009	Congo basin	Mandrills *(Mandrillus sphinx)*, African forest buffalo *(Syncerus caffer Nanus)*, elephants *(Loxodonta Africana*)	Duikers *(Cephalophus dorsalis*, *C*. *nigrifrons*, *C*. *weynsi*, *C*. *leucogaster*, *C*. *silvicultor*, *Philantomba monticola)*, mountain gorillas *(Gorilla beringei beringei)*, L’Hoest’s monkeys *(Cercopithecus lhoesti)*, chimpanzees *(Pan troglodytes)*, golden *monkeys (Cercopithecus kandti)*, Grauer’s gorillas *(Gorilla beringei graueri)*	[83]
2006–2007	Mayotte, Mauritius and Reunion Island	Ship rats (*Rattus rattus*), brown lemurs *(Eulemur fulvus*), crab eating macaques *(Macaca fascicularis)*.	Cat *(Felis catus)*, *dog (Canis lupus)*, horse (*Equus ferus*), cattle *(Bos primigenius)*, goat *(Capra aegagrus)*, sheep (*Ovis aries*), pig (*Sus scrofa*), poultry (*Gallus gallus*), shrew *(Suncus murinus)*, Norway rat (*Rattus norvegicus*), house mouse (*Mus musculus*), Hamadryas Baboon (*Papio hamadryas*), Southern pig-tailed macaque (*Macaca nemestrina*), Campbell’s monkey (*Cercopithecus Campbell*)	[84]
2008–2009	Thailand	Northern pig-tailed macaques *(Macaca nemestrina)*		[85]
2009–2010	Malaysia	Long-tailed macaques *(Macaca fascicularis)*		[86]

*Multiple species investigated in many articles;

** Possible sylvatic host

#### Transmission of CHIKV in non-human vertebrate hosts

Very few studies have been conducted outside Africa to investigate sylvatic transmission, but evidence from Africa has shown cyclic surges in CHIKV circulating among arboreal mosquitoes and vertebrate host populations [81]. A study on sylvatic transmission of CHIKV among Bornean orangutans was inconclusive as there was no evidence for CHIKV infection in the orangutans or humans from the local areas [82]. One experimental study demonstrated that genetically modified mice with deficiencies in their type 1 interferon response (IRF3/7-/-) were able to transmit CHIKV mouse-to-mouse without an arthropod vector. In addition, results from the same study showed that saliva from several infected monkeys was positive for the presence of viral RNA and infectious virus, which could potentially lead to CHIKV transmission without a vector [67].

The observational studies above provide data on possible sylvatic hosts and the ecology of CHIKV within the studied areas. Many experimental studies were conducted to understand the potential contribution of diverse non-human vertebrate hosts to CHIKV transmission by investigating the susceptibility of these hosts to the virus. A susceptible vertebrate host in these studies is defined as an animal that exhibits symptoms of CHIKV infection and/or detectible levels of viremia within the experimental host. There were 20 studies that looked at several NHP species and other vertebrate hosts listed in Table 3. General observations included higher susceptibility in infant mice, chicks, rats and rabbits, although this was not consistent across all studies. Several studies demonstrated that a number of NHP species, some rodents and bats were susceptible to CHIKV infection, while most wild birds, domestic and farm animals and adult experimental rodents were not susceptible hosts.

**Table 3:** Results from challenge trials investigating host susceptibility to CHIKV in non-human vertebrate hosts.

Reference	Susceptible host	Not Susceptible to CHIKV
[87]	Mice (A129) deficient in alpha/beta interferon signaling	
[88]	Infant mice	
[89]	Infant white leghorn chicks, infant rats	Sparrows, domestic pigeons, house rats, fruit eating bats, rabbits, guinea pigs and wild hare
[90]	Infant mice, 2 day old rabbits were moderately susceptible	Adult mice, rats, guinea pigs, rabbits, cats, fowl, kittens, and chickens
[91]	Mice < 6 weeks of age	Mice> 6 weeks of age, infant chicks
[92]	Mice (C57BL/6)	
[93]	Syrian hamster, mice (C57BL/6 and house mouse), big brown bats	Horse, calf, goat, wild boar, dog, European rabbit, American mink, armadillo, raccoon, chicken, mallard, red-winged blackbird, double-crested cormorant, American white pelican, house sparrow, rock pigeon, ring-billed gull, cattle egret
[94]	Syrian hamsters	
[95]	Japanese monkeys	
[96]	Rhesus monkeys	
[97]	Rhesus monkeys, Cercopithecus mona monkeys, chicks	Rabbit, rats
[98]	Infant mice, vervet monkeys,	
[99]	Rodents—Mystromys, Arvicanthis, Mastomys, Saccostomus, Aethomys, Tatera	
[100]	Rhesus monkeys	
[101]	Infant mice, monkeys	Adult mice
[102]	Rhesus monkeys	
[103]	Mystromys albicaudatus rodents, vervet monkeys, hamsters	
[104]	Mice (Swiss albino), Rhesus monkeys	
[67]	Mice (IRF3/7-/-), Cynomolgus monkeys	
[105]	Infant mice, Rhesus monkeys, rabbits, guinea pigs	Adult mice,

### CHIKV in humans

Chikungunya in humans was studied in 1159/2326 (50%) articles: 1040 observational and 49 experimental studies. The experimental studies comprised 3/49 (6%) challenge trials for potential vaccine candidates. Of the controlled trials (17/49; 35%), four studied vaccination in humans. All vaccination studies were at the experimental or trial stages. The efficacy of drugs to treat chikungunya symptoms was assessed in 3/17 (18%) controlled trial studies in humans. Other controlled trials included *in vitro* studies using human blood cells from volunteers to assess potential antiviral or vaccine candidates (11/17; 65%).

#### Epidemiology and surveillance in humans

There were 126/208 (61%) surveillance articles reporting on CHIKV in humans (Table 1), including 36/126 (29%) articles on travel-related syndromic surveillance and 2/126 (2%) articles on surveillance for CHIKV in the blood donor system. Active surveillance for CHIKV in humans during epidemics was reported in 26/126 (21%) studies, often in a hospital or tertiary care setting. Some results originated from resource limited countries where large epidemics were occurring and the surveillance protocol involved public health staff going door-to-door in impoverished neighbourhoods to identify CHIKV cases [106–108]. Monitoring programs in epidemic settings were reported in 6/126 (5%) human surveillance studies. These were systematic and purposeful programs mainly focussed on collecting numbers of chikungunya patients without a rigid action plan. Passive surveillance, mainly syndromic surveillance for CHIKV during epidemics was reported in 14/126 (11%) articles where data on chikungunya patients were collected in a hospital or other healthcare setting. In non-epidemic settings, there were 43/126 (34%) active surveillance articles for CHIKV in humans, 17/126 (13%) articles on monitoring programs, and 57/126 (45%) articles on passive surveillance.

Most of the epidemiological studies were on humans (848/912; 93%); Table 1. These included 117/848 (14%) epidemic investigations and 242/848 (29%) prevalence surveys. Human CHIKV prevalence results representing the burden of CHIKV during epidemics were reported in 16/242 (6.6%) studies, while 134/242 (55%) studies investigated the presence of antibodies against CHIKV in populations after an epidemic. Incidence was reported in 51/848 (6%) studies with 12/848 (1%) studies reporting CHIKV incidence during epidemics, while attack rates were reported in 31/848 (4%) studies soon after the epidemic. CHIKV related mortality was reported in 36/848 (4%) studies and in many cases death was associated with a pre-existing chronic morbidity [109–110]. The viremic period for CHIKV infection was reported in 30 studies and ranged from the first day of illness to 23 days (one report of prolonged viremia in a pediatric case detected at day 23 of illness [111]. The intrinsic incubation period reported in 13 studies also varied between studies and ranged from two to twelve days. One study reported evidence of possible sequential CHIKV infection [112].

Risk factors for developing chikungunya infection in humans were investigated in 345 studies. Landscape associated risk factors were reported in 100/345 (29%) studies with variables such as coastal (8/100), inland (3/100), elevated (5/100), urban (24/100), suburban or peri-urban (8/100), rural (21/100), farm or agricultural land (3/100), forest (5/100), parks (2/100), private gardens (7/100) and type of dwelling (19/100) investigated. Climatic indices as risk factors were investigated in 75/345 (22%) studies with precipitation (28/75; 37%), season (34/75; 45%) and temperature (9/75; 12%) predominantly studied. Other risk factors studied were occupation (26/345; 8%), outdoor recreational activities (4/345; 1%), storage of tires (4/345; 1%) and stagnant water on property, in containers, vases and flower pots (23/345; 7%). Travel to and from endemic areas was reported as a risk factor in 215/345 (62%) studies. Chikungunya incidence was reported to be highest in individuals aged 55–64 years and lowest in the 0–14 age group [113]. Other risk factors for chikungunya were pre-existing chronic conditions such as Type 2 diabetes, cardiovascular disease [114–117] and obesity [118–119].

#### CHIKV epidemics

Over 70 chikungunya epidemics have occurred in several countries across many continents between 1959 and 2016 and were documented in 141articles; Tables 1 and 4. The number of large epidemics increased steadily since the E1:A226V mutation occurred in CHIKV when *Ae*. *albopictus* became another and more efficient competent vector. During the epidemics in the Indian Ocean Islands in 2005–2006, a third of the population was estimated to be infected [120–122] and both *Ae*. *aegypti* and *Ae*. *albopictus* were found to carry the virus [123]. Over half the population was reported to be infected with CHIKV during a 2007 epidemic in Kerala, India [124–125]. Several articles described results from major epidemics across the Indian subcontinent from 2006 onwards (39/141; 28%). Epidemic investigation reports after CHIKV emergence in the Caribbean and South America also indicated high prevalence of exposure [92, 126–127]. These results depict the spread of CHIKV over time, either as an emerging pathogen in a new area, or a re-emerging public health risk in previously affected regions.

**Table 4:** Human CHIKV epidemics reported between 1959 and 2016.

Continent	Country	Epidemic start	Refid
Africa	Rhodesia	1959	[128]
Africa	Zimbabwe "Southern Rhodesia"	1962	[129]
Asia	Thailand	1962	[130]
Asia	India	1963	[131]
Asia	India	1964	[132–134]
Asia	Sri Lanka	1965	[135]
Asia	India	1965	[136]
Asia	India	1969	[137]
Asia	India	1973	[138]
Africa	South Africa	1977	[139]
Africa	Senegal	1982	[140]
Asia	Malaysia	1988	[141]
Africa	Democratic Republic of Congo	1999 and 2000 (3 epidemics)	[142]
Asia	Indonesia	2001, 2002 (2 epidemcis)	[143]
Africa	Kenya	2004	[144]
Indian Ocean Islands	Reunion Island	2005	[109], [116], [145–156]
	Mayotte, Comoros	2005	[157]
Asia	India	2005	[158–160]
Africa	Madagascar	2006	[161]
Asia	Malaysia	2006	[28], [162]
Asia	India	2006	[27], [107], [163–172]
Asia	India	2006, 2009 (2 epidemics)	[30]
Asia	Maldives	2006	[29]
Asia	Sri Lanka	2006	[173–174]
Asia	India	2007	[124], [175–179]
Europe	Italy	2007	[56], [180–184]
Africa	Gabon	2007	[185–186]
Asia	India	2008	[3], [187–188]
Asia	Bangladesh	2008	[189]
Asia	Singapore	2008	[190–192]
		2008, 2009 (multiple clusters)	[193]
Asia	Malaysia	2008	[194]
Asia	Thailand	2008	[195–197]
		2009	[198]
Asia	India	2010	[106], [199–201]
Africa	Gabon	2010	[202–203]
Asia	China	2010	[204–206]
Indian Ocean Islands	Reunion Island	2010	[207–208]
		2010, 2011 (2 clusters)	[121]
Asia	India	2011	[209–210]
Asia	Bangladesh	2011	[211–212]
Australasia	New Caledonia	2011	[213]
Africa	Republic of Congo	2011	[214–215]
Asia	Yemen	2011	[216]
Asia	Philippines	2011	[217]
Australasia	Papua New Guinea	2012	[218–219]
Asia	Lao People’s Democratic Republic	2012	[220]
Asia	Bhutan	2012	[221]
Asia	Cambodia	2012	[222]
Asia	Philippines	2012	[223–224]
Asia	Bangladesh	2012	[225]
Asia	Lao PDR	2013	[226]
Central America/South America/Caribbean	St. Martin	2013	[31]
Central America/South America/Caribbean	St. Martin, St. Barthelemy, Martinique and Guadeloupe	2013	[32]
Central America/South America/Caribbean	French Guiana, Guadeloupe, Martinique, Saint Barthélemy, Saint Martin,Windward Islands, Leeward Islands	2013	[33]
Central America/South America/Caribbean	Dominica	2013	[227]
North America	USA	2014	[228]
North America	Mexico	2014	[22]
Asia	India	2014	[229]
Europe	France	2014	[230–231]
Australasia	French Polynesia	2014	[232–233]
Central America/South America/Caribbean	Puerto Rico	2014	[234]
Central America/South America/Caribbean	Dominica	2014	[235]
Central America/South America/Caribbean	Colombia	2014	[236–243]
Central America/South America/Caribbean	Dominican Republic	2014	[244–245]
Central America/South America/Caribbean	Barbados	2014	[246]
Central America/South America/Caribbean	US Virgin Islands	2014	[113]
Central America/South America/Caribbean	French West Indies	2014	[247]
Central America/South America/Caribbean	Haiti	2014	[248]
Central America/South America/Caribbean	Brazil	2014	[21], [249]
Central America/South America/Caribbean	Suriname	2015	[250]

#### Travel-related chikungunya

There were 215/1159 (19%) articles reporting travel-related CHIKV infections; Table 1. These results included 74/215 (34%) studies where travelers acquired their infections during travel to endemic countries, while 81/215 (38%) studies reported that travelers developed clinical symptoms days after returning to their home countries from a chikungunya endemic country where they were exposed to the virus. Of the travel-related studies, 29/215 (13%) studied prevalence of chikungunya infection among travelers.

### Clinical manifestation of acute phase CHIKV infection in humans

**Fig 3:**
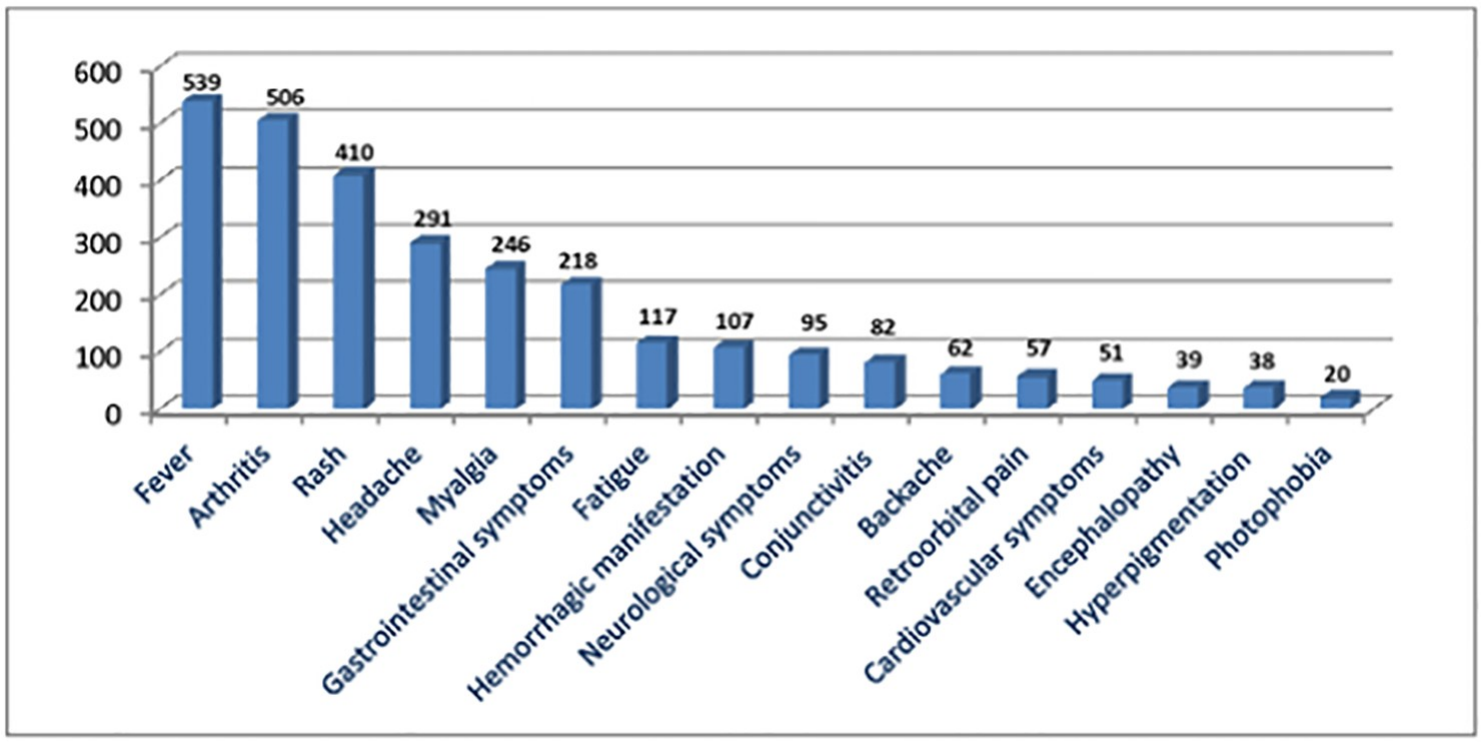
Number of studies reporting each sign or symptom of clinical CHIKV disease in humans*. *Multiple signs and symptoms reported in many articles.

A total of 764/2326 (33%) articles reported clinical signs and symptoms of chikungunya infection, of which 269/764 (35%) were case or case series reports; Table 1. The commonly accepted triad of CHIKV symptoms is fever (539/764, 71%), itchy rash (410/764, 54%) and joint pain (506/764, 66%), however, the literature reports many other frequently reported symptoms of CHIKV including: headache (291/764, 38%), myalgia or muscle ache (246/764, 32%), conjunctivitis (82/764, 11%), gastrointestinal symptoms such as nausea, vomiting, diarrhea or abdominal pain (218/764, 29%), and fatigue, malaise or weakness (117/764, 15%); Fig 3. Although described more often in association with DENV infections, 57/764 (7%) articles reported retro-orbital pain, 107/764 (14%) reported hemorrhagic disorders and 95/764 (12%) articles described neurological symptoms associated with chikungunya disease. These results do not represent the frequency of occurrence of these signs and symptoms as some articles reported exclusively on a single disease manifestation.

#### Atypical signs and symptoms

Atypical signs and symptoms of chikungunya infection were described in 121/764 (16%) articles, all with case / case series or cross-sectional study designs. Hyperpigmentation was reported as an atypical observation in 38/764 (5%) articles, and was first reported after the 2005–2006 epidemics in the Indian Ocean Islands. Other atypical observations seen increasingly after the Indian Ocean Island epidemics included severe ocular (15/764, 2%) [251–253] and neurological conditions (95/764, 12%) [254–256]; S4 Table.

#### Sequelae following CHIKV infection in humans

There were 239/764 (21%) articles describing long term sequelae associated with CHIKV; Table 1. Persistent post-infection morbidities included tendinitis (5/239; 2%), synovitis (14/239; 6%), balance disorders (1/239; 0.4%), and tunnel syndromes (11/239; 5%). There were 9/239 (4%) articles that reported Guillaine-Barré Syndrome (GBS) associated with chikungunya infection. Other long term complications reported were chronic arthritis (163/239; 68%), neurological disorders such as encephalopathies (18/239; 8%), acute disseminated encephalomyelitis (ADEM) (5/239; 2%), and ocular disorders (17/239; 7%). These post-chikungunya long term sequelae were reported to last from a few months to several years. A recent study suggests that hyperferritinemia may be a potential marker of chronic chikungunya disease [257].

#### Co-infections with CHIKV in humans

Chikungunya co-infections with either single or multiple co-infecting pathogens were reported in the literature. There were 109/1159 (9%) articles reporting co-infections with chikungunya: dengue (77/109; 71%), malaria (13/109, 12%) and zika (9/109; 8%); Table 1. There were four articles from the Americas that reported triple concurrent co-infections with CHIKV, DENV and ZIKV, all viruses that are transmitted by the same vectors [9,14, 258–259]. Other co-infections reported with CHIKV were Japanese Encephalitis, West Nile Virus, Tuberculosis, Human Immunodeficiency Virus (HIV), Leptospirosis, Yellow Fever, *Klebsiella pneumoniae* and *Pseudomonas aeruginosa*. A complete list of co-infections reported with CHIKV is compiled in S5 Table.

### Treatment for chikungunya infections in humans

Chikungunya disease is generally managed through supportive treatment and treatment strategies were reported in 283/2326 (12%) studies. Prescribed analgesics and anti-pyretics were reported in 87/283 (31%) studies, non-steroidal anti-inflammatory drugs (NSAIDs) in 80/283 (28%) studies and steroids in 70/283 (25%) studies; Table 1. Antibiotics were prescribed as treatment for chikungunya in 30/283 (11%) studies. There were 7/283 (2%) studies that described the use of antiviral drugs for chikungunya treatment and 29/283 (10%) studies described using an anti-malarial agent such as hydroxychloroquine to treat chikungunya symptoms. A randomized controlled trial examining the effectiveness of chloroquine to treat chikungunya concluded that there is no justification for its use during the acute stage of the illness [260]. Physical therapy or acupuncture were prescribed in 9/283 (3%) studies and 14/283 (5%) studies described the use of traditional medicine for treating chikungunya and its symptoms. The efficacy of plant-based drugs (19 studies), NSAIDs (17 studies), corticosteroids (19 studies), antivirals (17 studies) chloroquines (12 studies) and traditional medicines (8 studies) for treating acute and chronic symptoms of chikungunya was assessed in experimental studies.

#### CHIKV transmission in humans

All the major epidemics reported in Table 4 were due to the CHIKV urban transmission cycle where the virus is reported to be maintained between humans and *Ae*. *aegypti* and *Ae*. *albopictus* mosquitoes in tropical and sub-tropical regions of the world. Other potential modes of CHIKV transmission have been reported, including human exposure through infected blood transfusions (2/1159; 0.2%), or needle pricks with infected blood (3/1159; 0.3%); Table 1. Accidental CHIKV transmission was reported in three articles; two in laboratory staff working with infected mosquitoes [261–262], and one report of an infected nurse who was potentially exposed while drawing blood from an infected patient [263]. No studies investigated human sexual transmission of CHIKV as is frequently reported for ZIKV. One study found evidence of CHIKV transmission through corneal grafts and the authors propose banning cornea donations in CHIKV endemic areas [66].

Several studies (49/1159; 4%) reported mother-to-child or vertical transmission of CHIKV and many investigated fetal outcomes in neonates infected *in utero* or post partum; S6 Table. Pregnant women and neonates were the study subjects in 34/49 (69%) studies, while 13/49 (27%) studies reported on neonates alone. The impact of congenital CHIKV infection in neonates was reported in 16/49 (33%) studies and 6/49 (12%) studies investigated pregnancy outcomes due to CHIKV infection [5, 264–267]. One article recommended close monitoring of viremic pregnant mothers in maternity units. The same study reported that 12% of newborns are symptomatic, with many neonates requiring prolonged or intensive care due to complications such as meningoencephalits and intravascular coagulation [265].

### Public knowledge, attitudes and perceptions of chikungunya disease and CHIKV vectors

Knowledge, attitudes and perceptions about CHIKV, mosquito-borne diseases and their prevention were investigated in 46/2326 (2%) cross-sectional articles; Table 1. Articles on attitudes and perceptions to chikungunya disease focused on concerns about toxic or environmental effects of chemical control measures (3/46; 7%), perception about the severity and risks associated with chikungunya disease (16/46; 35%), and perception on the efficacy of various protective measures (13/46; 28%). Several studies evaluated knowledge on chikungunya disease (29/46; 63%), CHIKV vectors (27/46; 59%) and behavioral mitigation practices for CHIKV (33/46; 72%). A single study examined willingness to pay for protection against CHIKV [268].

### Economic burden of CHIKV

A total of 23/2326 (1%) studies investigated the economic burden of CHIKV, of which 19/23 (83%%) studied the economic burden of chikungunya disease in humans, 3/23 (13%) examined the cost-benefit of implemented vector control measures during epidemics and 1/23 (4%) reported the cost effectiveness of interventions to prevent the transmission of CHIKV through blood transfusions in Canada; Table 1, S7 Table. Publications on the economic burden due to CHIKV were predominantly from countries impacted by major epidemics and used data collected from surveillance during epidemics to estimate costs [122, 182, 269–271]. The epidemics from these areas were reported to have high attack rates, case fatalities and many severe long term sequelae, posing a burden to healthcare systems. All but one study were published after 2005; S7 Table. The 1980 study from Fiji calculated the cost effectiveness of vector control mitigation strategies in general [272].

### Mitigation strategies to prevent and control CHIKV and its vectors

A total of 323/2326 (14%) articles reported on mitigation or prevention and control strategies for CHIKV, mainly focusing on vaccination against CHIKV (78/323; 24%), personal protective measures (79/323; 25%), the use of insecticides (156/323; 48%), public education (48/323; 15%), quarantine of infected individuals (5/323; 2%) and biological control of mosquitoes (51/323; 16%); Table 1.

**Vaccination:** There were 78 studies evaluating potential vaccine candidates. Twenty-one included *in vitro* experiments, 42 used laboratory animals (mice and rabbits), seven used non-human primates and eight reported results from testing the potential vaccine on humans; Table 1. There was no validated and approved ready-to-use vaccine against chikungunya for humans identified from our data analyses. However, there were articles on two potential vaccine candidates. One demonstrated complete immunity against CHIKV in mice and NHPs [273], while the other, a novel live-attenuated vaccine candidate, has undergone a phase II clinical trial and is suggested as an option for emergency CHIKV vaccinations, in addition to being a potential vaccine candidate that can be further developed [274].

**Behavioral protective measures:** The 79/323 articles on personal protective measures included using insect repellant (38 articles), wearing full coverage clothing that prevents mosquito access to skin while outdoors (8 articles), using mosquito bed nets and window screens (36 articles), avoiding practices that lead to collecting stagnant water and removing suitable vector habitats, such as emptying pots, containers or tires (64 articles). The use of many of the protective interventions were evaluated before and/or after an education program to teach the general public or targeted population group about CHIKV (12 articles), personal and/or behavioral protective measures (7 articles), insecticides (5 articles) or all three aforementioned categories of measures (22 articles). Study designs varied from quasi-experiments and cross-sectional studies to predictive models of the various protective measures. Few articles report data on the effectiveness of personal protection interventions (38/79, 48%).

**Insecticidal use:** Insecticide use to control CHIKV vector populations was reported in 156/323 (48%) articles. Ten used a targeted ovicide (10/156; 6%),105/156 (67%) used a larvicide and 49/156 (31%) used an adulticide. Of all the articles reporting on insecticidal use, 24/156 (15%) articles evaluated the insecticide for its efficacy at the community level, while the remaining investigated plant extracts and other compounds experimentally for their efficacy as a potential insecticide. Two articles described the use of lethal ovitraps as a successful mitigation strategy against CHIKV vectors [275–276]. The use of larvivorous fish, beetles, nematodes or other copepods as a vector mitigation strategy was reported in 7/323 (2%) studies [277–283].

**Biological control strategies:** There were 51/323 (16%) articles describing biologic control of vectors as a mitigation strategy against CHIKV. Of these, 7/51 (14%) reported on Sterile Insect Technology (SIT), 14/51 (27%) were on Incompatible Insect Technique (IIT or Cytoplasmic incompatibility) and 2/51 (2%) articles described Release of Insects with Dominant Lethal mosquitoes (RIDL) as an effective strategy to mitigate CHIKV transmission through vectors. Infection of mosquito vectors with bacteria for controlling or reducing CHIKV replication was reported in 23/51 (45%) studies, with 12/51 (24%) studies examining the efficacy of *Wolbachia* and 11/51 (22%) investigating the role of *Bacillus thuringiensis* for effective control of CHIKV transmission through CHIKV carrying vectors. One study investigated silver particles synthesized by the fungus *Trichoderma atroviride* as a potential nanolarvicide against *Ae*. *aegypti* larvae and found this to be a very effective strategy for killing the larvae [284].

### Modeling studies

There were 103/2326 (4%) modeling studies including quantitative risk assessment articles on chikungunya disease or competent CHIKV vectors. Exposure to CHIKV, illness, or costs attributed to chikungunya disease in humans was the outcome modeled in 58/103 (56%) articles, 1/103 (1%) article modeled CHIKV spread through birds [285], while 70/103 (68%) modeled CHIKV vectors; mainly *Ae*. *aegypti*, *Ae*. *albopictus*, or both. Of these, 40/103 (39%) were spatio-temporal models, 48/103 (47%) were temporal models without a spatial component, 8/103 (8%) were spatial models without a time component and 7/103 (7%) models did not incorporate time or space. There were 9/103 (9%) risk assessment models estimating reproductive numbers or the magnitude of the threat posed by CHIKV to the general population. Modeling results were relevant to North America (Canada, USA and Mexico) (10/103, 10%), Central/South America and the Caribbean (15/103, 15%), Africa (3/103, 3%), Europe (21/103, 20%), Asia (17/103, 17%), Australia and New Zealand (2/103, 2%), Oceania (2/103, 2%) and Reunion Island (15/103, 15%). We reviewed 27/103 (26%) modeling articles that were general transmission models applicable to any region. Studies also included those that predicted an outcome, such as number of cases, incidence or prevalence rates (69/103, 67%), generated a better understanding of disease transmission (41/103, 40%), evaluation of mitigation measures or programs (28/103, 27%), or evaluation of the economic burden of CHIKV outbreaks (6/103, 6%). Other studies included travel associated importation models (11/103; 11%), models assessing risk through blood donation (9/103; 9%) and a single model on spread of CHIKV through transportation of cargo (1/103; 1%). Many modeling studies generated results that fell under more than one of the above mentioned categories.

## Discussion

Although CHIKV was first isolated in 1953, it is postulated that some epidemics dating back to the eighteenth century could have been caused by CHIKV and had been incorrectly attributed to DENV [134, 286]. Our chronological analysis of published CHIKV epidemic reports depicts the emergence and re-emergence of CHIKV across continents over time. A single mutation in the viral genome of CHIKV resulted in advantageous changes to the virus by increasing its fitness and ability to adapt to *Ae*. *albopictus*, a new vector that is more tolerant to cold temperature, subsequently changing the global distribution and risk profile of CHIKV [19].

### Transmission

Many studies report sylvatic transmission in Africa, where the virus is thought to be maintained by circulating between NHPs, rodents, reptiles and bats, with arboreal mosquitoes as vectors in the sylvatic cycle [24], however, there is limited research on sylvatic transmission outside Africa. While the urban transmission cycle between humans and mosquitoes is well understood, sylvatic cycles in areas outside Africa likely exist and may not be elucidated at the present time. Due to the hypermutability of CHIKV, it is conceivable that the virus could have adapted to new hosts and vectors in areas where CHIKV has expanded recently, thereby introducing a substantial risk for CHIKV to initiate a sylvatic cycle in the Americas [287]. A knowledge gap exists in our full understanding of vertebrate hosts that may contribute to the sylvatic transmission cycle and maintenance of CHIKV in endemic areas. Future research can focus on filling current knowledge gaps that could facilitate a better understanding of the roles of various hosts and vectors in CHIKV transmission cycles contributing to the spread, emergence and maintenance of CHIKV globally, as this knowledge is important for developing effective mitigation strategies and more accurate risk analyses.

Vertical transmission of CHIKV was observed in humans [146,149, 244, 288]. The impact of intra-uterine CHIKV infection on the fetus and neonates is not clearly understood with conflicting results from several studies. One study looked at dizygous twins born to a mother infected during pregnancy and reported one healthy neonate, while the other twin was severely ill due to congenitally acquired chikungunya [267]. Another study examining outcomes of CHIKV infection to the fetus during each trimester of pregnancy until post-partum reported vertical transmission in all three trimesters, with more serious outcomes to neonates when mothers were viremic at delivery [289]. It is important to keep in mind that vertical transmission of chikungunya in an asymptomatic infected mother or neonate can also trigger a cluster of cases among pregnant women or new mothers and neonates in maternity units. A single case of microcephaly in a neonate born to a mother with gestational chikungunya infection has been described [290]. Microcephaly is believed to be an outcome associated with ZIKV infections [291]. We acknowledge that this could be a coincidental finding, but CHIKV associated microcephaly certainly warrants future research given that CHIKV and ZIKV co-circulate in many regions. There has been no research on sexual transmission of CHIKV, despite evidence for this with ZIKV. Interestingly, viable CHIKV has been isolated from an individual’s semen and urine sample up to 30 days post-infection [74] and one study provided experimental evidence for the presence of infectious CHIKV in human saliva [67], so the possibility of sexual transmission of CHIKV in humans needs to be clearly elucidated.

The risk of viremic travelers importing CHIKV into non-endemic regions with competent vectors is quite high, as highlighted by locally transmitted epidemics in France [230–231]; Italy [182–183] and many more recent travel- related importations into the Americas [54, 59]. In non-endemic areas where CHIKV vectors are present, it is important for public health authorities and physicians to be aware of chikungunya disease and the possible epidemic risks associated with the importation of the virus into new areas through viremic travelers. Large epidemics are costly but can be controlled with proper prevention and control strategies such as personal protection against vectors and vector mitigation.

### CHIKV diagnostics

Although there are diagnostic tests to confirm clinical diagnosis, there is evidence for antigenic cross reactivity between CHIKV and other alphaviruses [292–293]. A rapid, field-ready and cost effective gold standard diagnostic test with high sensitivity and specificity for CHIKV has not been reported in the literature and would be useful, especially in resource limited settings. A wide array of samples have been successfully tested for CHIKV using currently available tests. While the most common sample was blood, others included corneas and minimally invasive samples such as urine and semen [74].

### Pre-existing risk factors for developing chronic sequelae

Although chikungunya is a self-limiting disease, it can result in long term sequelae, most often resulting in chronic arthritis. Researchers have postulated that pre-existing morbidities can predispose an individual to more severe chikungunya disease and chronic sequelae post-CHIKV infection [110] and that initial clinical manifestation of CHIKV illness can predict progression to chronic arthritis [294]. The list of pre-existing morbidities in CHIKV patients compiled from the literature can broadly be categorized as chronic respiratory, cardiovascular, immunological disorders, and Type 2 Diabetes. Further analysis of the literature on the relationship between pre-existing morbidities and the development of chronic sequelae could be explored in a systematic review to summarize the magnitude and confidence in any potential association that might exist.

### Atypical chikungunya disease

Several studies described atypical and severe forms of chikungunya disease during both acute and convalescent phases, as well as chronic morbidities associated with CHIKV infection. Many articles from India reporting on epidemics from 2006 onwards frequently describe hyperpigmentation as an atypical observation during and after the infection [295–297]. A study from Venezuela described unexpected nasal skin necrosis in a few patients with severe chikungunya disease [298]. It remains unclear whether this atypical sign is linked to a particular mutation in the virus or to host genetics. Interestingly, observations from our results show that hyperpigmentation is always reported in individuals with darker skin. In addition to hyperpigmentation, we observed several reports of severe ocular disease associated with chikungunya, ranging from varied forms of uveitis and other manifestations, to complete blindness [299–300]. Five studies described acute disseminated encephalomyelitis (ADEM) in CHIKV patients from India [256, 301–304]. It remains to be determined if any pre-existing morbidity is associated with these outcomes. Other neurological complications were infrequently observed across early chikungunya outbreaks, however, Guillain Barré Syndrome (GBS) is a relatively new complication associated with CHIKV infection reported in the literature since the Indian Ocean Island epidemics of CHIKV [27, 156, 247, 305–306]. A study on a chikungunya epidemic in French Polynesia reported an increase in the number of GBS cases [233]. These observations may be a result of larger epidemics with better reporting, or due to newly acquired genomic changes that contribute to higher virulence in the virus. ZIKV which often co-circulates with CHIKV has also been reported to be associated with GBS [307].

### Treatment

Management of chikungunya illness is predominantly supportive, with analgesics, steroids and NSAIDs that alleviate the symptoms of CHIKV rather than specifically target the virus. Chloroquine, an antimalarial, was often prescribed to manage CHIKV symptoms, even though its efficacy in alleviating symptoms during the acute phase of infection is questioned [260]. Some studies reported the use of antibiotics to treat CHIKV infections [30, 308–311], but their efficacy as an effective treatment option was not evaluated in any study.

### CHIKV lineages and genomic plasticity

Many outbreaks in India have been linked to different CHIKV lineages [312–313]. Since there is evidence for two separate virus introductions in Brazil with different lineages of CHIKV during the same epidemic period [21, 23, 314], it is important to keep in mind that there may be a strong likelihood that this can also occur elsewhere. With many reports of co-circulating CHIKV, DENV and ZIKV [8, 249, 259], it would be interesting to examine whether there is evidence for possible horizontal acquisition of genetic elements for virulence between co-circulating viruses in endemic areas. There has been a lot of research on CHIKV mutations and phylogeny for investigating the evolutionary relatedness of CHIKV isolates across epidemics in different geographical areas, as well as to identify genetic changes in the virus as it evolves over time. Many articles reported an important amino acid change in the EI outer membrane glycoprotein region of the virus with an alanine to valine substitution at position 226 [315–316]. This mutation reportedly allowed the virus to gain *Ae*. *albopictus* as a new and highly competent vector to aid in its transmission more efficiently [317]. This mutation contributed significantly to the global expansion of risk areas for chikungunya and also highlights the plasticity of CHIKV and its potential to mutate, adapt and emerge or re-emerge in different areas. There is great concern that more selective mutations in the virus will facilitate its success in efficiently gaining more competent vectors and host reservoirs from invasive species [316], and if this occurs, public health risks from CHIKV could increase globally. At the same time, there is also evidence of genetic stability in CHIKV isolates, where isolates that share a common origin but were collected over many years and in different regions maintained a high degree of identity [18, 318]. Several CHIKV mutations associated with adaptation and fitness were reported in the literature and are compiled in this ScR.

### Burden of chikungunya disease

One of the challenges of estimating the true burden of CHIKV is due to a high proportion of asymptomatic and mild cases who may not seek medical attention. This underestimation can affect results for predictive models, mitigation strategies and other analyses that use surveillance data as inputs. Misclassification of chikungunya as dengue or zika infections could also occur as these infections are often co-circulating and share overlapping symptoms [7, 319–320], therefore prevalence results reported on the basis of clinical signs and symptoms should be considered with caution. A complete understanding of whether co-infections with CHIKV can lead to altered symptoms or sequelae is also required. One study reported evidence of possible sequential CHIKV infection [112] and more studies are needed to validate this.

CHIKV epidemics can result in great financial burdens to affected countries. The 2014 CHIKV epidemic in Colombia was estimated to cost at least 73.6 million USD and put an enormous burden on that country’s economy [238]. It is imperative for countries at risk for CHIKV to have effective and efficient surveillance systems and mitigation strategies to alleviate the potential impact from future epidemics. The best way to mitigate public health risks from CHIKV is by preventing exposure to the virus through personal protective measures and the implementation of efficient vector control strategies to curb viral transmission.

### Limitations of this study

This ScR was conducted by using rigorous and reproducible synthesis research methodology, however, it is possible that some articles were not identified by our search or search verification strategy. Excluding articles that were not in English, French, Spanish or Portuguese could contribute to potential language bias and may have resulted in an underrepresentation of some geographic regions in our results. The impact of these exclusions is unknown. The lapse in time between the updated search and the completion of the analysis for this study has certainly resulted in our inability to capture information from many recently published papers and is a limitation of our study. However, we believe that this limitation is offset by the rigour and thoroughness of our methodology and the included literature adequately represents affected areas around the world. We presume that the impact of missing research is minimal. The articles used for data extraction and the dataset generated from this project mailto:are available in the DRYAD digital repository and can be accessed using the review link: DOI: https://doi.org/10.5061/dryad.qd65020.

## Conclusion

In conclusion, this ScR identified and characterized all available literature on CHIKV and allowed us to capture important information from the many epidemic waves observed with CHIKV infection over time. We have reported important observations relating to trends in disease manifestation, long term sequelae associated with CHIKV infection, plasticity of the viral genome, important epidemiological characteristics, as well as characteristics of surveillance and monitoring programs typically used. In addition, our results describe the extent of research on treatment strategies, mitigation methods for preventing or controlling CHIKV disease and/or CHIKV vectors. Global evidence on treating and preventing CHIKV can be used to inform future public health initiatives. The ScR repository also includes a comprehensive list of research on important mutations that are potentially linked to evolution, adaptation and fitness of the virus. Within these results, knowledge gaps and areas with abundant research were noted. In-depth knowledge synthesis projects and a risk assessment project have used data generated from this ScR as inputs [321–322].

Results from this ScR can be used to inform policy-makers and researchers on the evidence base for CHIKV and point to where additional research is needed to close knowledge gaps.

## Supporting information

S1 Checklist PRISMA checklist. (DOC)

S1 Protocol ScR on Chikungunya infections—Protocol. (DOCX)

S1 Expert Questionnaire Expert Questionnaire. (DOCX)

S1 Table List of CHIKV genetic variants for fitness and adaptation. (DOCX)

S2 Table Diagnostic tests and samples. *Multiple diagnostic tests reported in many articles.
(DOCX)

S3 Table Diagnostic test evaluation. *Multiple tests evaluated in many articles.
(DOCX)

S4 Table Atypical signs and symptoms. (DOCX)

S5 Table Coinfections with CHIKV. *Pathogen not specified.
(DOCX)

S6 Table Materno-fetal transmission. (DOCX)

S7 Table Economic burden of CHIKV. (DOCX)
