# Supplementary material for: A scoping review of published literature on chikungunya virus
Source: PLoS One. 2018 Nov 29;13(11):e0207554. doi: 10.1371/journal.pone.0207554 (PMC6264817; doi:10.1371/journal.pone.0207554)
Supplement: S1 Protocol — (DOCX) [file pone.0207554.s002.docx]

# Title:

A scoping review of research investigating Chikungunya Virus and its competent vectors

# Authors:

Mariola Mascarenhas^1*^, Lisa Waddell^1^, Judy Greig^1^, Philippe Berthiaume^1^, Sophiya Garasia^1,2^

^1^Public Health Risk Sciences Division of the Laboratory for Foodborne Zoonosis, Public Health Agency of Canada.

^2^Department of Population Medicine, University of Guelph

Contact: Mariola Mascarenhas, Tel: 519-826-4185 or email: [mariola.mascarenhas@phac-aspc.gc.ca](mailto:mariola.mascarenhas@phac-aspc.gc.ca)

# Important Dates:

Evidence published up to January 6, 2017

Protocol version 1, initiated February, 2015

Contents

[Title: 1](#_Toc482350084)

[Authors: 1](#_Toc482350085)

[Important Dates: 1](#_Toc482350086)

[Background 3](#_Toc482350087)

[Objectives of the ScR 5](#_Toc482350088)

[Study Question 5](#_Toc482350089)

[Study Sub- Questions 5](#_Toc482350090)

[Planned Study Outputs 6](#_Toc482350091)

[Methods 6](#_Toc482350092)

[Review Team Expertise and Responsibilities. 6](#_Toc482350093)

[Search Strategy 7](#_Toc482350094)

[Algorithms 7](#_Toc482350095)

[Databases 7](#_Toc482350096)

[• Scopus, PubMed/MEDLINE, Embase, CINAHL (Cumulative Index to Nursing & Allied Health), CAB, LILACS (South American), Agricola, COCHRANE library for any relevant trials in the trial registry 7](#_Toc482350097)

[Grey Literature Sources and Procedures 7](#_Toc482350098)

[Search Verification 7](#_Toc482350099)

[Relevance Screening (RS) 8](#_Toc482350100)

[Inclusion / Exclusion criteria 8](#_Toc482350101)

[Study Characterisation 8](#_Toc482350102)

[Review Management 8](#_Toc482350103)

[Data Analysis 8](#_Toc482350104)

[Appendix 1: Relevance Screening Tool 9](#_Toc482350105)

[Appendix 2: Second level relevance screening 13](#_Toc482350106)

[Appendix 3: Data Characterization Form 19](#_Toc482350107)

[Appendix 4: Search Strategy Implemented 47](#_Toc482350108)

[Databases 47](#_Toc482350109)

[Grey Literature 47](#_Toc482350110)

[Search Verification 47](#_Toc482350111)

# Background

Chikungunya (CHIK) infections in humans are caused by the chikungunya virus (CHIKV), an *alphavirus* transmitted by two predominant species of mosquitoes, namely, *Aedes aegypti* and *Ae. Albopictus.* Challenge studies conducted in *Ae. hensilli* show that this mosquito species is very sensitive to infection with CHIKV and can also be a suitable vector for it (Ledermann et al)*.* Although this disease does not contribute largely to mortality, it causes severe morbidity through acute, sub-acute and chronic conditions such as disabling arthralgias. CHIKV is spread from the blood of an infected person through a female mosquito vector into the bloodstream of a susceptible individual. As with many infectious diseases, high risk groups for CHIK infections are the elderly, the very young and immunocompromised individuals. Specific vaccines and targeted drugs are currently unavailable for CHIK infections.

CHIKV has steadily made its way into the Americas; however, autochthonous transmission has not occurred in Canada and most of the United States to date, except for the state of Florida, which was recently reported to have locally transmitted cases. *Aedes aegypti* and *Ae. Albopictus* species have not yet inhabited Canada, although the latter is known to survive and overwinter in the egg stage in temperate and cold temperate climates (WHO). With the inevitability of climate change and acclimatization of many vector species, there is a possibility for a CHIKV-competent vector to become indigenous in Canada, thereby increasing the risk of CHIKV infections to susceptible Canadians.

There are three genotypes for CHIKV; West African, East/Central/South African (ECSA) and Asian. Research suggests that CHIKV incurred a mutation that enabled its transmission using *Ae. Albopictus* as a new competent vector *(*Enserink M et al., Tsetsarkin KA et al., ProMED- mail Archive Number 20071209.3973). In November 2014, Brazil reported a novel and more serious genotype of CHIKV that was locally transmitted (Scientific American, November 2014). This is the first novel and more serious genotype for CHIKV documented in the Americas, and poses a great threat if it spreads to epidemic proportions.

Several diagnostic tests are available for detecting CHIKV in blood, CNS, synovial fluid and other samples from infected patients. These tests are reported to have varying sensitivity and specificity in terms of diagnostic capability. The timing of the sample post-infection seems to play a large role in the accuracy of the diagnostic test results.

CHIKV can be transmitted from infected blood donors to recipients. Experimental animal models have shown that infected donors can be asymptomatic with high enough viraemic levels to cause disease (Appassakij H, et al). So far, there have not been any reports of CHIKV transmission in humans through transfusion or organ transplantation. There have been reports of mother-to-child transmission, particularly when women develop the disease towards the very end of their pregnancies (Gerardin P et al., Ramful D et al).

Prevention and control strategies for CHIKV largely involve curtailing the breeding of mosquitoes by minimizing standing water in the environment and chemical spraying of areas to kill mosquitoes or larvae. In addition, using insect repellants, wearing protecting clothing and using protective netting also prevent humans from being infected by mosquitoes harbouring CHIKV.

***Rationale:***

Over many decades, CHIKV ravaged countries in Asia and Africa, causing several epidemics. Numerous countries in those regions are now endemic for CHIK infections. In fact, this virus is quickly expanding its habitat across geographic borders and has recently spread to countries such as Italy, Croatia, France and other Indian and Pacific Ocean islands (WHO). Since late 2013, there have been several reports of CHIK infections in South American countries that have never previously seen this virus, and these numbers are steadily growing. Many countries across Europe and North America are seeing a growing number of travel-related CHIK cases. It is imperative to have early detection systems for imported cases in place to prevent further CHIKV transmission, especially in countries with competent CHIKV vectors.

To date (February 18, 2015), there have been 2,320 imported CHIK cases in the USA from across 8 US states (CDC and CCDR). There have been 11 locally transmitted cases in the state of Florida (CCDR: Volume 41-01, January 8, 2015), with most reported cases being travel- related (CDC). CHIK infections are now nationally notifiable in the USA (CDC).

A report from the Public Health Agency of Canada (PHAC) published in January 2015 (CCDR: Volume 41-01, January 8, 2015), identifies a surge in the number of travel-related chikungunya cases that have been diagnosed in Canada. The report identifies 320 confirmed cases and an additional 159 probable cases (Fig 1) in Canada, and attributes these soaring numbers to travel within countries in the Caribbean and the Americas, and due to the geographical expansion of CHIKV. Currently, chikungunya is not a nationally reportable infection in Canada.


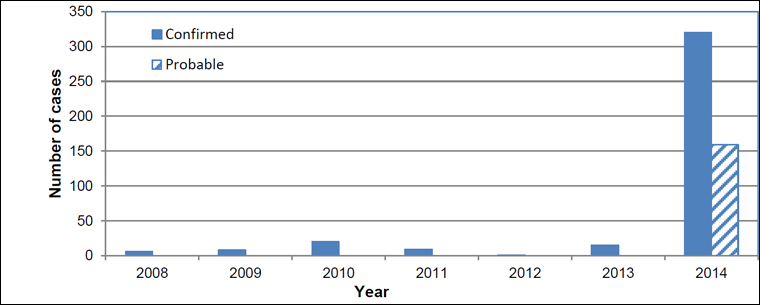


**Fig 1: Number of confirmed and probable CHIKV cases in Canada since 2008. Source of graph: Drebot et al., CCDR: Volume 41-01, January 8, 2015**

More than 1.2 million suspected cases have been reported from the Americas alone within the last two years (PAHO), highlighting the rapid emergence of this pathogen in previously uninhabited areas. Due to the increasing threat of expansion of this virus, it poses to be a huge public health concern and requires strong and constant vigilance from public health authorities to ensure that these infections do not reach epidemic proportions.

Conducting a scoping review of all the literature available on chikungunya infections will enable us to compile and analyse relevant information pertinent to prevention and control strategies for mitigating the introduction and development of this disease in Canada. Effective and efficient strategies can be put in place in a timely manner with this collated and readily available information. There is a need to systematically summarize the current state of knowledge regarding CHIKV surveillance and potential prevention and control measures, to support evidence-informed decision-making that ensures enhanced surveillance and effective education activities to address this emerging public health issue. A comprehensive review of current knowledge can also highlight areas where future field and experimental work is needed to fill in existing knowledge gaps surrounding CHIK disease.

# Objectives of the ScR

## Study Question

*What is the current state of research knowledge on Chikungunya virus (CHIKV) including its diagnosis, surveillance methods, prevention and controls strategies, risk factors, social attitudes and perceptions and the epidemiology of the virus, infection in humans, animals and vectors and characteristics of competent CHIKV vectors and mitigation strategies to control these vectors?*

This research aligns with PHRSD priorities, which include enhancing and guiding public health decision-making and policies by providing the authoritative analyses, recommendations and scientific collaborative services (using methods such as epidemiological studies and knowledge synthesis) to address the occurrence, trend and determinants of infectious disease in Canada with expert focus on the prevention of public health risks arising from the food chain, animals and the environment (LFZ, 2013).

## Study Sub- Questions

The scoping review will aim to capture all published literature addressing the following aspects of CHIK infections *(to be discussed and agreed upon by the advisory committee)*:

- Surveillance methods for CHIKV harbouring mosquitoes and CHIKV infections [vectors, animal reservoirs, humans]
- The effectiveness of prevention/intervention/control/education programs or strategies [vectors, animal reservoirs, humans]
- Risk factors for developing CHIK infections in humans and mosquito exposure/abundance risk factors
- Societal attitudes and perceptions towards CHIK infection prevention and control strategies
- Diagnostic/detection tests for CHIK infection in humans and for the presence of CHIKV in animal reservoirs, or vectors
- Treatment options for CHIKV infection
- Economic burden of CHIK disease and/or cost-benefit analysis of potential prevention and control strategies
- Vector adaptation, competence (suitability) and transmission
- Molecular characterization of virus
- Clinical characteristics and complications of CHIKV infections/presence in humans and animal reservoirs
- Prevalence/incidence of CHIKV infections in humans and of CHIKV in animal reservoirs and vectors
- Reports of outbreaks and sporadic cases

## Planned Study Outputs

1. Primary scoping study of the review findings.
2. A summary of findings fact sheet.
3. A repository and dataset of all relevant literature captured in this study.

# Methods

## Review Team Expertise and Responsibilities.

**Hypothesis Generation Working Group**

**Members and Roles**

| **Member** | **Organization** | **Project Role*** |
| --- | --- | --- |
| Mariola Mascarenhas | RISK - Guelph | Project Lead  Synthesis expertise - participant |
| Dr. Lisa Waddell | RISK - Guelph | Synthesis expertise- participate as needed |
| Judy Greig | RISK - Guelph | Synthesis expertise – participate as needed |
| Dr. Philippe Berthiaume | PED – St. Hyacinthe | Modeling expertise - participant |
| Dr. Ian Young | Ryerson University | Synthesis expertise- participate as needed |
| Dr. Victoria Ng | LFZ (PHRSD) | Advisory |
| Dr. Nick Ogden | CFEZID | Advisory |
| Dr. Harvey Artsob | Retired NML | Advisory |
| Dr. Martin Petric | BCCDC | Advisory |
| Dr. Pablo Martinez de Salazar | CARPHA | Advisory |
| Dr. Raymond Tellier | University of Calgary | Advisory |
| Tricia Corrin |  | Participant |
| Catherine Hierlihy |  | Particpant |
| Sophiya Garasia |  | Participant and co-administrator |
| Danielle Reimer |  | Participant |

## Search Strategy

### Algorithms

### Databases

### Scopus, PubMed/MEDLINE, Embase, CINAHL (Cumulative Index to Nursing & Allied Health), CAB, LILACS (South American), Agricola, COCHRANE library for any relevant trials in the trial registry

### Grey Literature Sources and Procedures

***Grey Literature searched:***

- WHO library (including SEARO, IMSEAR, IMEMR)
- The World Bank (*databank.worldbank.org/data/databases/infectious-diseases*
- CDC – MMWR, FastStats – Infectious Disease ( [www.cdc.gov/nchs/faststats/infectious -disease.htm](http://www.cdc.gov/nchs/faststats/infectious%20-disease.htm), ArboNET
- PHAC
- PHO
- National Institutes of Health (NIH)
- Australia’s **National Notifiable Disease Surveillance System (NNDSS)**
- European Centre for Disease Prevention and Control (ECDC)
- **ENHanCEd Infectious Diseases (EID2 database); www.zoonosis.ac.uk/eid2**
- **Communicable Diseases Intelligence – Australian government’s Department of Health**
- PAHO
- Eurosurveillance
- Global Health Database (<http://www.lshtm.ac.uk/library/resources/databases/info_globalhealthovid.html>)
- ProMED-mail
- Infochangeindia.org
- Asia Development Bank
- IndMED
- MedCarib
- Caribbean Public Health Agency (www.carpha.org)

### Search Verification

A snowball strategy for search verification will be used. This may include all relevant paper references being screened for potential relevance and if not already included in our ScR database, they will be added to the review for screening.

- Hand searching MMWR, CDC website
- Screening reference lists (10 reviews)

## Relevance Screening (RS)

The relevance screening level will be done on the title, abstract and keywords where available. Two questions will be e used to quickly determine the relevance of a citation. The questions are based upon the inclusion / exclusion criteria defined with the help of our topic expert advisors. The RS tool can be found in the appendix.

### Inclusion / Exclusion criteria

***Potential inclusion/exclusion criteria (to be defined in consultation with our expert advisors)***

1. Time frame – no time frame
2. Country – All
3. Language – English, French, Spanish and Portuguese
4. Document Type: All - any peer review primary articles, reviews, commentaries, PhD/MSc Theses

## Study Characterisation

The characterisation level of this scoping study is to first confirm relevance of a publication and second to extract all important information from a citation to understand the value and characteristics of CHIKV infections. This will include; study design, evaluation of cost, timeliness, surveillance methodologies and results, reported risk factors for susceptibility to infection or environmental risk factors contributing to mosquito exposure/abundance, CHIKV epidemiology, short and long-term complications of CHIKV infections, diagnostic tests and their accuracy, CHIKV infection treatment, potential prevention and control measures and their effectiveness, attitudes and perceptions towards CHIKV infections, …..

## Review Management

The search strategy will be compiled and deduplicated in a RefWorks database. This database will then be exported to DistillerSR, a web-based systematic review software designed to manage all stages of conducting scoping reviews and systematic reviews. All stages of the scoping study from relevance screening to data extraction will be conducted within this software. The final dataset will be exported into MS Excel, cleaned and tabulated for use in the publication and reports.

## Data Analysis

This will likely be a descriptive tabulation of all pertinent information regarding CHIKV; including risk factors, surveillance, diagnostics, prevention and control, and perceptions and attitudes. Findings and recommendations, methods incorporated and their usefulness, and study limitations will also be captured. All knowledge gathered will be summarised and discussed.

## Appendix 1: Relevance Screening Tool

*What is the current state of research knowledge on pathogenesis, surveillance methods, prevention and control strategies, risk factors and, knowledge, societal attitudes and perceptions towards chikungunya (CHIK) infections in humans and chikungunya virus (CHIKV) in mosquito vectors and animal reservoirs?*

**Relevance Screening Tool for Abstracts:**

| **Question** | **Options** | **Definitions/additional notes** |
| --- | --- | --- |
| RefID | Will be retrieved automatically from Distiller. |  |
| 1. Does the abstract investigate:  1) Chikungunya infections in humans, CHIKV in any host or vector?  OR  2) Studies on CHIKV (e.g. pathogenesis, transmission and molecular mechanisms)  OR  3) Strategies to:  a) prevent CHIKV infections  OR/AND  b) control CHIKV harbouring vectors  OR  4) Environmental/ climatic conditions for vector adaptation  OR  5) Studies on aedes agypti and/or aedes albopictus abundance, presence, mortality, longevity, behaviors (e.g. biting rate), activity and extrinsic incubation period |  Yes – primary research on CHIKV or CHIKV harbouring vectors  No – Review on CHIKV or CHIKV harbouring vectors  No (excluded, submit form) | **At this initial stage of the scoping study:**  **If the article is not on Chikungunya infections in humans, Chikungunya virus in mosquitoes or vertebrate hosts, prevention/control of CHIKV vectors, it is not relevant!**  Chikungunya infections are caused by the chikungunya virus (CHIKV) and are transmitted to humans through bites from mosquito vectors that harbor CHIKV.  CHIKV, an *alphavirus,* is transmitted by two predominant species of mosquitoes, namely, *Aedes aegypti* and *Ae. Albopictus.* Known CHIK vectors include but are not limited to:  Aedes aegypti, Aedes Albopictus Aedes africanus Aedes camptorhynchites Aedes dalzieli Aedes furcifer-taylori Aedes fulgens Aedes luteocephalus Aedes vigilax Aedes vittatus Culex annulirostris  Culex gelidu Mansonia uniformis  CHIKV is spread from the blood of an infected host through a female mosquito vector into the bloodstream of a susceptible individual. Humans and animals act as reservoirs. Animal reservoirs include **monkeys, birds, cattle and rodents (may include others); transmission is also possible from animal reservoirs to humans** |
| **ONLY PROCEED IF YOU ANSWERED YES TO Q1** | | |
| **2. Is the main focus likely one of the following (note most studies will only have 1 possibly 2 areas of focus)** |  Yes   - Surveillance methods used to determine the extent of Chikungunya infections in humans and/or Chikungunya virus in animal reservoirs or mosquito vectors - Evaluation of diagnostic tests for CHIK in humans or detection tests for CHIKV in animal reservoirs or mosquito vectors - Pathogenesis of CHIK disease - Risk factors reported for Chikungunya infections in humans or for the occurrence of CHIKV in animal reservoirs or mosquito vectors - Efficacy of mitigation strategies to prevent and/or control Chikungunya infections in humans or CHIKV in animal reservoirs or mosquito vectors - Public and health professionals/physicians knowledge, attitudes and/or risk perceptions towards CHIK and potential prevention and control strategies - Economic burden of CHIK infections and/or cost-benefit of potential prevention and control strategies - Molecular characterization of CHIKV (e.g. mutations) - Vector adaptation/suitability and transmission - Diagnosis, clinical characteristics and complications of CHIKV infections - Burden of illness   - Prevalence and/or incidence (exclude)   - Outbreaks and/or sporadic cases    No (excluded, submit form) | Public health ***surveillance*** is the ongoing and systematic collection, analysis, and interpretation of outcome-specific data for use in the planning, implementation, and evaluation of public health practice. This includes numbers of cases, or geographic coverage of Lyme endemic areas. **Please include studies evaluating surveillance methods/programs.**  Examples of relevant applications include:   - Surveillance of human cases - Sylvatic host surveillance (Sylvatic cycling is when pathogen transmission occurs between animal i.e., sylvatic hosts and vectors) - Mosquito/vector surveillance   ***Diagnostic tests*** for detecting the presence of CHIKV in humans, other non-human hosts and vectors  ***Pathogenesis*** refers to the biological processes/mechanisms/pathways that lead to CHIK disease  ***Risk factors*** are environmental, behavioural, or biologic factors usually in longitudinal, cross-sectional, cohort or case control studies where exposures and outcomes are studied. A risk factor indicates an association with an increase or decrease in disease in the population with the risk factor compared to that without.  ***Mitigation strategies = interventions***   - Studies looking at intervention efficacy include control or challenge trials and quasi experiments (before and after). - Program evaluations can fall in here. - Risk factors looking at presence/absence of an intervention should also be checked here. - Examples include (but are not limited to) land management, vector management and control, personal protection, and public education campaigns   If any CHIK-harboring vector is mentioned without the mention of CHIKV and the paper is on prevention/control, include the paper  ***Risk perceptions*** *are* the subjective judgements that people make about the characteristics and severity of a risk. Do individuals feel they are at risk? Do they have knowledge that they can implement to decrease their risk? What are their feelings concerning using sprays or treating mosquitoes to decrease the risk of disease transmission?  ***Economic burden*** will include an actual dollar amount or discussion of implied cost associated with mitigation strategies.    ***Cost benefit analysis*** is a systematic process for calculating and comparing benefits and costs of a project, decision or government policy. It has two purposes:   - to determine if it is a sound investment/decision (justification/feasibility), - to provide a basis for comparing projects. It involves comparing the total expected cost of each option against the total expected benefits, to see whether the benefits outweigh the costs, and by how much. |

## Appendix 2: Second level relevance screening

*What is the current state of research knowledge on chikungunya virus (CHIKV) including its pathogenesis, diagnosis, surveillance methods, prevention and control strategies, risk factors, societal attitudes and perceptions and the epidemiology of the virus, infection in humans, animals and vectors and characteristics of competent CHIKV vectors and mitigation strategies to control those vectors?*

**Second Relevance Screening Tool**

| Question | Responses | Notes |
| --- | --- | --- |
| 1. Does the article focus on one of the following categories:  1) Chikungunya infection in humans, CHIKV in any host or vector, or social/economic impact of CHIKV  2) Studies on CHIKV (e.g. pathogenesis, transmission, infection mechanisms including viral entry/exit, virulence factors, evolution and phylogeny)  3) Strategies to:  a) prevent CHIKV infections  b) control CHIKV harboring vectors  4) Environmental/climatic conditions for vector adaptation (option 2)  5) Studies on Aedes Aegypti and/or Aedes Albopictus abundance, presence, mortality, longevity, behaviour (e.g. biting rate), activity and extrinsic incubation period (option 2) | - Yes, on CHIKV topic - Yes, only on competent vector, no CHIKV - No (Exclude, please submit) | Article is deemed relevant if the focus of the paper is on one or more of the mentioned categories. |
| If exclusion criteria was selected above, submit the form before proceeding | | |
| 2. Is the article published in English, French, or Spanish? | - English - French - Spanish - Portuguese - Other, please specify: ____ (Exclude, please submit) |  |
| If exclusion criteria were selected above, submit the form before proceeding | | |
| 3. What type of document is this article? | - Primary research or model in peer-reviewed journal - Thesis - Grey literature with primary data (government or research reports) - Conference proceedings (Exclude, please submit) - Literature review (Exclude, please submit) - Systematic review/meta-analyses (Exclude, please submit) - Grey literature; may report previously reported research (newspaper or magazine articles; exclude, please submit) | **Primary research:** original research/investigation/study carried out by the researcher (incl. surveys, interviews, outbreak reports, observations, etc.)  **Thesis:** a long paper/essay or dissertation involving personal research (usually written for a university degree)  **Conference proceeding abstract/short paper:** A collection of published academic papers  **Literature review:** Examination of published literature  **Systematic review/meta-analyses:** Analysis and interpretation of primary research  **Grey literature:** Research that is unpublished or published in a non-commercial form |
| If exclusion criteria were selected above, submit the form before proceeding | | |
| 4. What is the subject/species studied in the article? | - Humans - Vector, mosquitoes - Other vector, please specify: ____ - Non-human vertebrate hosts, please specify: _________ - Virus only - Other, please specify ___ | Reservoirs of CHIKV include but are not limited to: humans, monkeys, rodents and birds. CHIKV can spend time in the sylvatic cycle (fraction of the pathogen’s lifespan spent cycling between non-human hosts and vectors) |
| 5. Where was the study conducted? (specify country) | - North America, please specify: ______ - Europe, please specify: _______ - Australasia, please specify: ________ - Central America/South America/Caribbean, please specify: ______ - Asia, please specify: ____ - Africa, please specify:_____ - Indian Ocean Islands, unknown continent (includes Seychelles, Reunion, Mauritius) | **North America:** Includes Canada, USA and Mexico  **Europe:** includes, Belarus, Latvia, Ukraine, Estonia, Cyprus & west (incl. Iceland and Greenland)  **Australasia:** limited to Australia, New Guinea, New Zealand, New Caledonia, and neighbouring islands, including the Indonesian islands from Lombok and Sulawesi eastward  **Central America/South America/ Caribbean:** Includes Caribbean, and all of south and central America.  **Asia:** Russia, Turkey, middle eastern countries and east  **Please specify country in the text box with full name (exception: USA)** |
| 6. When was the article published? | - Pre 1960 - 1960-1970 - 1971-1980 - 1981-1990 - 1991-2000 - 2001-2010 - 2011-Present |  |
| 7. What do the results of the paper focus on?  (Check all that apply; When answering this question, only check off the topics for which there are study outcomes and do not check if a category was just “mentioned” in the paper.) | - Surveillance of CHIK infection in humans and/or CHIKV in animal reservoirs or vectors - Epidemiology of CHIKV related to the host, vector and/or CHIKV (includes human, non-human and vector hosts) - Pathogenesis (cellular level to signs and symptoms) of CHIKV in hosts and vectors - Signs and symptoms of CHIK infection (Includes studies on how to diagnose CHIKV) - Treatment of CHIKV infection - Accuracy of diagnostic tests for CHIKV - Mitigation strategies to prevent/control CHIKV - Social impact papers including public and health professionals risk perceptions, attitudes and/or knowledge - Economic burden or cost-benefit analysis of CHIKV infection and/or mitigation strategies - Predictive model, please specify:______ - CHIKV **vector** studies - Insecticide resistance - Characteristics of competent vector (genes, adaptations, etc...) - Range and density of CHIKV vector and/or environmental/climatic conditions to sustain vector population - Vector activity (biting rate, Fecundity/fertility rate, reproductive rate, vector daily mortality rate) - Extrinsic incubation period (Interval between the uptake of CHIKV by vector and vector’s ability to transmit CHIKV to other susceptible hosts) - Transmission/rate of infectivity (ie: how many people could be exposed by one infected mosquito and how many mosquitos are likely to become infected by one viraemic human) - Other: ___ - CHIK **virus** studies - Molecular characterization of CHIKV (e.g. mutations, phylogeny) - CHIKV pathogenic attributes (e.g. virulence factors, viral entry/exit/cycle (includes latency period), viral replication) - CHIKV transmission and adaptability - Sylvatic-arbovirus dynamics - Survival of CHIKV - Other:____ - Other relevant topic, please specify: ____ (Use if absolutely necessary) | ***Surveillance:*** is the ongoing and systematic collection, analysis, and interpretation of outcome-specific data for use in the planning, implementation, and evaluation of public health practice. This includes numbers of cases, or geographic coverage of CHIK endemic areas. Please include studies evaluating surveillance methods/programs.  ***Epidemiology:*** Please include articles describing outbreak and sporadic cases, incidence/prevalence for CHIKV, and/or risk factors for developing CHIK infection or risk factors/conditions (environmental and climatic mostly) for CHIKV survival in vectors.  ***Pathogenesis*** refers to the biological processes/mechanisms/pathways that lead to CHIK infection and/or disease in vector or host. This includes the following:   - Infection mechanisms (at cellular level, stages of infection) including CHIKV entry/exit or inhibitors of CHIKV entry/exit - Immune response (Proteins/genes/receptors involved; in host and vector) - Pathology of disease (chronic or acute symptoms and organs affected) - Animal models studying pathogenesis - Signs and symptoms: Check if clinical signs and symptoms of CHIKV infection in humans and/or data on how to diagnose CHIKV are described in this paper   ***Diagnostic tests*** refer to tests detecting the presence of CHIKV in humans and other non-human hosts (including vectors).  ***Mitigation strategies*** are interventions to control CHIKV infection, CHIKV-harboring vectors or CHIKV. This includes program evaluations, quasi experiments, and/or studies looking at presence/absence of intervention. Examples include (but are not limited to) land management, vector management and control, personal protection, and public education campaigns  ***Economic burden*** will include an actual dollar amount or discussion of implied cost associated with CHIKV infection.    ***Cost benefit analysis*** is a systematic process for calculating and comparing benefits and costs of a project, decision or government policy.  ***Predictive models*** are mathematical or statistical models used to forecast outcomes, spread of CHIKV and/or trends. Examples include (but are not limited to) using climate to predict outbreaks and/or models predicting high-risk populations. In the provided text box, please describe model in one line. If possible, copy and paste text from the abstract/objectives section.  ***Vector studies*** include:   - Insecticide resistance - Vector mortality - Characteristics of competent vector (genes, adaptations, etc...; ability to transmit disease) - Range and density of CHIKV vector and/or environmental/climatic conditions to sustain vector population - Vector activity (biting rate, Fecundity/fertility rate, reproductive rate etc.) - Extrinsic incubation period (Interval between the uptake of CHIKV by vector and vector’s ability to transmit CHIKV to other susceptible hosts) - Transmission/rate of infectivity (ie: how many people could be exposed by one infected mosquito and how many mosquitos are likely to become infected by one viraemic human)   ***CHIK Virus studies*** include:   - Molecular characterization of CHIKV (e.g. mutations, phylogenetic analysis) - CHIKV pathogenic attributes (Describes how viruses cause disease e.g. virulence factors, viral entry/exit/cycle (includes latency period), viral replication) - CHIKV transmission (passing of virus from an infected host to another vector/host; e.g. mosquito transmission, mother to child and/or through blood transfusion) and adaptability (ability to adapt to new host/environment or become resistant to drug) - Check sylvatic-arbovirus dynamics if results discuss sylvatic cycle (In this cycle, the virus cycles between vector/arthopod and non-human hosts) - Survival of CHIKV (This includes studies on survival of CHIKV in environment, vector and/or host |

#

## Appendix 3: Data Characterization Form

**Broad topic:**

*What is the current state of research knowledge on Chikungunya virus (CHIKV) including its diagnosis, surveillance methods, prevention and controls strategies, risk factors, social attitudes and perceptions and the epidemiology of the virus, infection in humans, animals and vectors and characteristics of competent CHIKV vectors and mitigation strategies to control these vectors?*

**Article-level data characterisation and utility form (applicable to all relevance-confirmed articles)**

**Note:** Remember to only extract information in that applicable question – **not all questions apply.** Be very specific about the data you extract AND only **extract primary information** (information collected by the author in the course of the experiment.)

| Question | Options | Definitions/Additional notes |
| --- | --- | --- |
| What is the study design?  *(Check all that apply)* | - Observational study   - Cross-sectional   - Cohort   - Case-control   - Prevalence survey   - Surveillance or monitoring program   - Case study or case-series   - Outbreak investigation   - Sporadic cases investigation   - Longitudinal study   - Evaluation of diagnostic tests   - Other, specify: _____ - Experimental study - Controlled trial - Challenge trial - Quasi experiment - Other, specify:_____ - Qualitative study, specify:________ - Mixed methods - Economic model - Disease transmission model - Risk assessment - Vector mapping model - Other, please specify: ___ - Virus study (includes only vaccines, potential treatments or molecular characterization of the virus) - N/A (Virus pathogenesis only studies; Stop reviewing and submit form) - Pathogenesis of CHIKV in humans - Pathogenesis of CHIKV in mosquitoes - Viral pathogenesis (no humans) - Non-relevant mosquito paper - Other non-relevant study | **Observational study**: Assignment of subjects into a treated group versus a control group is outside the control of the investigator.   - **Cross-sectional:** Examines the relationship of a risk factor and outcome (disease) at a point in time on representative samples of the target population. - **Cohort study**: is a study in which individuals with differing exposures to a suspected risk factor are observed through time for occurrence of an outcome - **Case-control study**: compares exposure to the risk factor in subjects who have an outcome (the 'cases') with subjects who do not have the outcome, but are otherwise similar (the 'controls') and drawn from the same sampling frame. - **Prevalence survey:** Measurement of an outcome at a point in time but doesn’t measure or investigate potential predictors – include here routine monitoring or surveillance data collection - **Case or case-series:** a descriptive study of a single individual (case report) or small group (case series). - **Outbreak investigation:** Studies an outbreak retrospectively or while it is occurring. An outbreak is a sudden increase in the occurrence of CHIKV illness in a given area with linked cases - **Sporadic cases investigation:** Studies cases of CHIKV infection that are not linked in space and/or time. - **Longitudinal study:** A research method in which data is gathered for the same subjects over a period of time.   **Evaluation of Diagnostic Tests:** One or more diagnostic test is evaluated for sensitivity, specificity against a gold standard, clinical symptoms or another test.  **Experimental study:** Each subject is assigned to a treated group or a control group before the start of the treatment   - **Control trial:** an experimental study in which people are allocated to intervention groups and evaluated for outcomes. - **Challenge trial:** An experiment where subjects are artificially challenged or exposed to the disease agent and then allocated to the intervention groups for evaluation of the outcome. - **Quasi-experiment:** An experiment in which subjects are not randomly assigned to groups. Often this is the method of choice in field trials where the samples of the outcome are taken from the same individuals before and after the experiment/intervention   **Qualitative study:** Aimed at understanding social phenomena, exploring issues, and answering questions of “why” and “how.” Please specify the design/methodology that is identified by the author, and if none is identified explicitly then indicate “not specified”  **Mixed methods:** Tackles a research question using different research methodologies.  **Economic models** use mathematical equations to describe how costs are affected by different inputs. The structure of the equations reflects the model builder’s attempt to describe reality.  **Disease transmission models** are mathematical models used to link the biological process of transmission and the emergent dynamics of infection at the population level.  **Risk assessment** is the determination of quantitative or qualitative value of risk related to a situation and a recognized threat (hazard). Quantitative risk assessment requires calculation of the magnitude of the potential loss and the probability that the loss will occur.  **Vector mapping** is collection of data on spread/range of vector population (may use GIS)  **N/A- Virus pathogenesis only studies: Exclude studies** on pathogenesis. Don’t exclude studies on treatment, mutations/phylogeny and/or mitigation studies. |
| What host species were investigated?  *(Check all that apply)* | ** Humans**  **Samples taken to test for CHIKV**  ****Blood sample  ****Other sample, specify:  ____  ****Contextual information collected (e.g. questionnaire, survey, interview), specify: ____  **Characterize the human population ( or case report) sampled for CHIKV**   General population   Specific age demographic   - Paediatric - Adults - Elderly   ** Immunocompromised**   - Drug induced - Physiological - Pregnant - Other, specify: ____   ** Co-infection**   - Dengue and CHIKV - Other, specify: ___   ** Co-morbidity**   - Diabetes - Cardiovascular - Other, specify: ___   ** Mosquitoes**  *Aedes aegypti*  *Aedes albopictus*  *Aedes dalzieli*  * Aedes tunestus*  *Aedes furcifer*  *Aedes hensilii*  *Aedes luteocephalus*  * Aedes polynesiensis*  *Culex* species, specify: ____  Other, specify: ___  ** Wild animals, species _____**  Was the species investigated for sylvatic cycle transmission?  Yes, specify species investigated: ___  Was the species identified to be a part  of the sylvatic cycle?  Yes, specify species identified: ___  No  No    ** Domestic pets, species______**  Was the species investigated for sylvatic cycle transmission?  Yes, specify species investigated: ___  Was the species identified to be a part  of the sylvatic cycle?  Yes, specify species identified: ___  No  No  ** Wild birds, species _____**  Was the species investigated for sylvatic cycle transmission?  Yes, specify species investigated: ___  Was the species identified to be a part  of the sylvatic cycle?  Yes, specify species identified: ___  No  No    ** Farm animals, species______**  Was the species investigated for sylvatic cycle transmission?  Yes, specify species investigated: ___  Was the species identified to be a part  of the sylvatic cycle?  Yes, specify species identified: ___  No  No  ** Non-mosquito arthropods, species______**    ** Animal model**  Mice   - Was the species tested for susceptibility? - Yes, species found to be susceptible: ____ - Yes, species found not to be susceptible: _____ - No   Non-human primates, please specify____   - Was the species tested for susceptibility? - Yes, species found to be susceptible: ____ - Yes, species found not to be susceptible: _____ - No   Other, please specify: _____   - Was the species tested for susceptibility? - Yes, species found to be susceptible: ____ - Yes, species found not to be susceptible: _____ - No   ** No specific host. Study focuses on an in-vitro vaccine, treatment and/or phylogeny** | Examples of **type of sample** includes blood samples, CSF, and FFPE (Formalin-fixed-paraffin-embedded tissue) samples. Contextual information includes questionnaires, focus groups and surveys.  Population sampled for CHIKV refers to age range of the population studied. If it is a case report, specify age in epidemiology section.   - **General population (**0-infinity). No focus on a specific age group - **Specific population** selected when authors differentiate between age groups. For the purpose of this review: - **Pediatric** < 16 - **Adults**: 16 to 65 - **Elderly** >65   **Immunocompromised** Examples are persons with weakened immune systems include those with AIDS; cancer and transplant patients who are taking certain immunosuppressive drugs; and those with inherited diseases that affect the immune system   - **Drug induced**: Includes steroid treatment - **Physiological** is due to gene defect/spleen removed. It can be congenital or due to infections such as HIV.   **Wild animals** includes all wild animals other than birds and arthropods. Example of wild animals include chimpanzees, wild rabbits, and rodents.  In the **sylvatic cycle**, the virus cycles between vector/arthopod and non-human hosts  Example of **domestic pets** include cats and pet rabbits.  **Farm animals** includes chickens, cattle, sheep and all domesticated fowl  **Non-mosquito arthropods** includes ticks, fleas and other insects.  **N/A** includes cell-cultures, in-vitro models. |
| Please describe any specific population or geographical attributes (including the sampling site) as stated by the author | **[text]** |  |
| What is the molecular characterization of CHIKV as described by the authors?  *(Check all that apply)* | **Genotypes**   West African   East/Central/South African (ECSA) only   Epidemic IOL (Indian Ocean Lineage)   Asian (No specific lineage)   Asian –Indian lineage   Asian- Southeast Asian lineage   Other____   Not reported   N/A, no molecular characterisation or no CHIKV.  **Mutations**   A226V E1 (or E1/A226V)   E1 A98T   E1 D284   E1 V269   E2 1211T   E2 T2111   E2 L210Q   E2 K252Q   E2 S118G   E2 R198Q  ** Other ____   Not reported   N/A no molecular characterisation or no CHIKV. | **Not reported:** Authors did test CHIKV but didn’t report its molecular characterization or mutations.  **Not applicable:** . No aspects of the paper involved CHIKV.  **A226V E1 or E1/A226V** is the mutation that caused the Indian Ocean Islands outbreak. **Not reported:** Mutation not reported. Only looked at it at the macro level |
| What do the results of the study focus on?  *(Check all that apply)* | - Surveillance of CHIK infection in humans and/or CHIKV in animal reservoirs or vectors OR surveillance of vectors - Epidemiology of CHIKV related to the host, vector and/or CHIKV - Signs, symptoms and diagnosis of CHIK infection in humans - Treatment of CHIKV infection - Accuracy of diagnostic tests for CHIKV - Mitigation strategies to prevent/control CHIKV - Social impact papers including public and health professionals risk perceptions, attitudes and/or knowledge - Economic burden or cost-benefit analysis of CHIKV infection and/or mitigation strategies - CHIKV vector studies - Predictive models - Other, specify: ___________ | Studies on pathogenesis of CHIKV in hosts and vectors (cellular level) and studies on CHIK virus will not be further characterized.  **Surveillance** is the systematic and ongoing collection, collation, and analysis of health-related information. Please check this box if surveillance programs (for hosts and/or vectors) were already ongoing prior to the study, or if new programs were put in place as a result of reported CHIKV cases.  **CHIKV vector studies** include studies on mosquito competence, vector behaviour, CHIKV diagnosis in mosquitoes and /or extrinsic incubation period  **Other** includes those that were misclassified in RS2 |
| If Surveillance is selected please answer the questions below with respect to the ongoing surveillance program that existed prior to the study or incident cases. | | |
| Please describe the environment/site where samples were taken?  *(Check all that apply)* |  Low-density housing   Medium-density housing   High-density housing   Private home gardens   Parks   Pools/beaches   Farms/agricultural landscape   Forest   Storage/Shipment   Tires   Dracaena (lucky bamboo plants)   Other, please specify,_____   Other please specify, _____   Not reported   N/A | **Low-density housing** includes houses surrounding city but are sparsely  located  **Medium-density housing** includes suburbs  **High-density housing** includes cities  with built environments and urban areas  **Pools** are man-made and can be containers that are filled with water. Examples include public pools and ocean pools. **Beach** is a landform/ strip along the coast of the ocean or sea (salt-water) or edge of a lake or river (fresh-water)  **Shipment** is the transportation of goods by water, land or air. |
| Please describe the sample used for surveillance (What are they sampling?)  *(Check all that apply)* |  Humans   Travel related   Blood transfusion related   Mosquitoes   Wild animals, species _____   Domestic pets, species______   Wild birds, species _____   Farm animals, species______   Non-mosquito arthropods, species______   Other, please specify_____  N/A | In text box, only provide species name. Example: cats, dogs, rabbits |
| What is the goal of the surveillance system/program? | **[text]** | Please copy and paste the goal of the surveillance system. For example, to identify the number of mother-to-child CHIKV transmissions. |
| Describe region under surveillance | **[text]** | Describe area (i.e. urban/rural) and comment on scale of it (i.e. size of area sampled) |
| When did the surveillance program start? (yyyy/mm/dd) | **[text]** | State NA if not provided. |
| When did the surveillance program end? (yyyy/mm/dd) | **[text]** | If it’s still on-going, write “on-going” in the end date section |
| What surveillance methods are described?  *(Check all that apply)* |  Active:   Targeted sampling protocol used: ____   Monitoring program   Targeted sampling protocol used: ____   Passive   Public reporting/submitting of mosquitoes   Physician/ veterinarian reporting   Syndromic surveillance   Laboratory-based   Event-based   - Web-based aggregators (e.g. ProMed, GPHIN)    Social media programs (e.g. Twitter, Google)   Other, specify: _____ | **Sampling protocol:** Describe the sampling strategy used. Ex. 10 shipping containers checked, 200 km^2^ area searched  **Active surveillance**, in contrast to passive surveillance, requires that public health staff take direct action to collect disease information. For example, they may contact physicians, hospitals, laboratories, or other health entities to actively search for disease cases. Active surveillance may also occur through direct review of clinical or hospital charts, laboratory records, or emergency room patient logs. Active surveillance provides the most complete picture of disease incidence, i.e., cases are found in a timely manner, a greater number of cases are found, and more thorough information is obtained compared to passive surveillance methods. Active surveillance is an on-going activity and contains thresholds.  **Monitoring program:** Systematic purposeful program without active action plan. Simply counts numbers.  With **passive surveillance**, a member of the reporting community initiates a disease report that is communicated to a health department. For example, a physician may telephone a health department to discuss a case immediately upon seeing a patient with a suspected or confirmed case of a disease or an infection control practitioner may contact a health department upon receipt of positive laboratory results for a more common disease.  **Laboratory surveillance** differs from population-wide surveillance in that it can only monitor patients who are already receiving medical treatment and having lab tests done - does not identify patients who have never been tested. |
| What activities were triggered by reaching the surveillance system’s threshold? | - Public health activites_ ___ - Entomology activites_____ - Other activites______ | **Describe all activities that were carried out when the system was triggered to activate:**  - public health: case interviews, case finding activities, contact tracing, education etc.  -entomology activities: mosquito trapping and evaluation/ testing, mitigation to eliminate or control vectors  - Other.? |
| Did the author evaluate the surveillance program? |  Yes, specify: ____   No | **Specify** results of evaluation briefly |
| If Epidemiology is selected | | |
| What population does this study represent? | **[text]** | Specify country/city/state as specified by author and note any peculiar population attributes (if anything stands out that is not specific to general population). For example, the population studied was all males. |
| What is the **burden** of CHIKV in humans?  *(Only answer if the data is a population sample)* |  The sample represents [ date/ region/ population] = ____________   Prevalence , specify: __________   Incidence , specify:______   Case-fatality rate , specify:____   Long-term sequelae, specify: ____ | **Sample:** describe what the sample represents – date, region, population sampled.  **Prevalence:** It is the number of cases of CHIKV in a defined population at a specific point in time. Record both numerator and denominator if provided [# of total CHIKV cases at a point in time, # of exposed individuals)  **Incidence:** It is the number of new cases of CHIKV arising within a given time period in a specified population. Record both numerator and denominator if provided [# of new CHIKV cases in a given time period, # of exposed individuals)  **Case-fatality rate:** Proportion of cases that die from all CHIKV cases  **Long-term sequelae:** Proportion of cases that develop chronic symptoms |
| Were any human transmission characteristics reported?  *(Only answer if primary data is available)* |  Intrinsic incubation period (IIP), please specify in days: ___  Viremic period, please specify in days: ___  Other, specify: ____ | **The intrinsic incubation period (IIP)** is the time between a human being infected and the onset of symptoms due to the infection.  **Viraemic period** is the time period in which humans are infectious with CHIKV. |
| What is the burden of CHIKV in mosquito vectors? *(Only answer if the data is a population sample)* |  The sample represents [ date/ region/ population] = ____________   Prevalence, specify: ___   Co-infection, specify: ____   Other measure of burden of infection, specify: ____ | **Sample:** describe what the sample represents – date, region, population sampled.  For the **prevalence,** include the species name, number captured and the number of positive CHIKV mosquitoes as [species, #positive, # captured]  For **co-infection**, specify the name of the co-infection. Exclude studies on Wolbachia and only capture any human illness causing virus (e.g., Dengue). |
| What is the burden of CHIKV in non-human animal hosts? *(Only answer if the data is a population sample)* |  The sample represents [ date/ region/ population] = ____________   Prevalence, specify: ___   Co-infection, specify: ____   Other measure of burden of infection, specify: ____ | **Sample:** describe what the sample represents – date, region, population sampled.  For the **prevalence,** include the species name, number tested and the number of positive CHIKV mosquitoes as [species, # tested,#positive]  For **co-infection**, specify the name of the co-infection |
| Describe the human CHIKV **outbreak**  *(Only answer if this is an outbreak report)* |  Outbreak cases; total number reported:____   Outbreak start date (yyyy/mm/dd): __---   Outbreak finish date (yyyy/mm/dd): ____   Number of confirmed cases:____   Number of probable cases:____   Number of hospitalizations: ___   Number of fatalities: ____   Number of cases reporting chronic symptoms: _____   Mother-to-child transmission   Number of pregnant cases, specify:_____   Number of newborn cases, specify:_____ | **Outbreak start date** is when the primary case exhibited symptoms  **Confirmed cases** include all CHIKV cases that are laboratory confirmed.  **Probable cases** are cases that are clinically diagnosed without laboratory confirmation |
| Describe the CHIKV sporadic cases.  *(Only answer if this is a sporadic case report)* |  Sporadic cases; total number reported:____   Number of confirmed cases:____   Number of probable cases:____   Number of hospitalizations: ___   Number of fatalities: ____   Number of cases reporting chronic symptoms: _____  Mother-to-child transmission   Number of pregnant cases,  specify:_____   Number of newborn cases,  specify:_____ | **Sporadic cases** = When you see cases here and there. There is nothing linking one case to another.  Note: A travel-acquired case can be a sporadic case |
| Does this study report on any travel-related cases? |  Yes; specify visiting/travel country_________   Symptoms developed while case was in visiting country   Symptoms developed after returning from the visiting country   No, not reported |  |
| What risk factors were investigated for **developing CHIKV infection** investigated (ie: sampled and tested, not just mentioned)  (Only applies to epidemiology studies: surveys, cross sectional, case control, cohort. Not outbreak investigations)  *(Please check all that apply)* | **Geographic**   Coastal area   - Statistically significant risk factor, protective - Statistically significant risk factor, increased risk - Not statistically significant    Inland   - Statistically significant risk factor, protective - Statistically significant risk factor, increased risk - Not statistically significant   Urban   - Statistically significant risk factor, protective - Statistically significant risk factor, increased risk - Not statistically significant   Suburban or peri-urban   - Statistically significant risk factor, protective - Statistically significant risk factor, increased risk - Not statistically significant   Rural   - Statistically significant risk factor, protective - Statistically significant risk factor, increased risk - Not statistically significant    Private dwelling   - Statistically significant risk factor, protective - Statistically significant risk factor, increased risk - Not statistically significant    Private garden   - Statistically significant risk factor, protective - Statistically significant risk factor, increased risk - Not statistically significant    Park   - Statistically significant risk factor, protective - Statistically significant risk factor, increased risk - Not statistically significant   Cemetery   - Statistically significant risk factor, protective - Statistically significant risk factor, increased risk - Not statistically significant    Swimming pool   - Statistically significant risk factor, protective - Statistically significant risk factor, increased risk - Not statistically significant   Salt-water beach   - Statistically significant risk factor, protective - Statistically significant risk factor, increased risk - Not statistically significant   Fresh-water beach   - Statistically significant risk factor, protective - Statistically significant risk factor, increased risk - Not statistically significant    Farm/agricultural landscape   - Statistically significant risk factor, protective - Statistically significant risk factor, increased risk - Not statistically significant    Forest   - Statistically significant risk factor, protective - Statistically significant risk factor, increased risk - Not statistically significant    Transportation of goods by land: ___   - Statistically significant risk factor, protective - Statistically significant risk factor, increased risk - Not statistically significant   Transportation of goods by water: ___   - Statistically significant risk factor, protective - Statistically significant risk factor, increased risk - Not statistically significant   Transportation of goods by air: ___   - Statistically significant risk factor, protective - Statistically significant risk factor, increased risk - Not statistically significant    Latitude/Longitude   - Statistically significant risk factor, protective - Statistically significant risk factor, increased risk - Not statistically significant    Elevation/altitude   - Statistically significant risk factor, protective - Statistically significant risk factor, increased risk - Not statistically significant    Other geographic   - Statistically significant risk factor, protective - Statistically significant risk factor, increased risk - Not statistically significant   **Climate**   Precipitation, specify: ___   - Statistically significant risk factor, protective - Statistically significant risk factor, increased risk - Not statistically significant    Season, specify:   - Statistically significant risk factor, protective - Statistically significant risk factor, increased risk - Not statistically significant    Relative humidity, specify: ____   - Statistically significant risk factor, protective - Statistically significant risk factor, increased risk - Not statistically significant    Atmospheric moisture, specify:____   - Statistically significant risk factor, protective - Statistically significant risk factor, increased risk - Not statistically significant    Minimum humidity, specify: ____   - Statistically significant risk factor, protective - Statistically significant risk factor, increased risk - Not statistically significant    Maximum humidity, specify: ____   - Statistically significant risk factor, protective - Statistically significant risk factor, increased risk - Not statistically significant    Temperature, specify: ____   - Statistically significant risk factor, protective - Statistically significant risk factor, increased risk - Not statistically significant    Minimum temperature, specify: ____   - Statistically significant risk factor, protective - Statistically significant risk factor, increased risk - Not statistically significant    Maximum temperature, specify: ___   - Statistically significant risk factor, protective - Statistically significant risk factor, increased risk - Not statistically significant    Mean temperature, specify: ___   - Statistically significant risk factor, protective - Statistically significant risk factor, increased risk - Not statistically significant    Maximum temperature of warmest month   - Statistically significant risk factor, protective - Statistically significant risk factor, increased risk - Not statistically significant    Minimum temperature of coldest month   - Statistically significant risk factor, protective - Statistically significant risk factor, increased risk - Not statistically significant    Other climatic risk factor, specify: ____   - Statistically significant risk factor, protective - Statistically significant risk factor, increased risk - Not statistically significant   **Human behaviours**   Occupational risk, specify__   - Statistically significant risk factor, protective - Statistically significant risk factor, increased risk - Not statistically significant    Outdoor recreational activities (e.g. picnics, camping), specify__   - Statistically significant risk factor, protective - Statistically significant risk factor, increased risk - Not statistically significant    Walking or jogging   - Statistically significant risk factor, protective - Statistically significant risk factor, increased risk - Not statistically significant    Gardening or yard work   - Statistically significant risk factor, protective - Statistically significant risk factor, increased risk - Not statistically significant    Storage of unused tires   - Statistically significant risk factor, protective - Statistically significant risk factor, increased risk - Not statistically significant    Emptying containers and pots   - Statistically significant risk factor, protective - Statistically significant risk factor, increased risk - Not statistically significant    Maintenance of standing water on property, specify____   - Statistically significant risk factor, protective - Statistically significant risk factor, increased risk - Not statistically significant    Travel related   - Statistically significant risk factor, protective - Statistically significant risk factor, increased risk - Not statistically significant    Blood transfusions   - Statistically significant risk factor, protective - Statistically significant risk factor, increased risk - Not statistically significant    Other, specify ____   - Statistically significant risk factor, protective - Statistically significant risk factor, increased risk - Not statistically significant | **Coastal area**: Area where land meets the sea/ocean  **Urban:** Location characterized by high human density and that has been significantly developed (density of human structures such as houses, commercial buildings, roads, bridges, etc… )  **Suburban:** An area located on the outskirts of a city/developed area. Usually it is a residential area. Peri-urban is an area immediately adjoining an urban area. It’s an area where you have a mixture of urban and rural activities. The term peri-urban is commonly used in Europe, India and Africa.  **Rural:** Area where you have very few homes and very low population density.  **Swimming/wading pool** is man-made. It is a container that is filled with water. Examples include public pools and ocean pools.  **Beach** is a landform/stip along coast of ocean or sea (salt-water) or edge of a lake or river (fresh-water)  **Transportation of goods by land** includes trucks carrying loads of cargo such as bamboo sticks. **Transportation of goods by water** include carrying cargo in river boats, ships, etc. **Air transport** includes airplanes carrying goods/passengers.  Specify whether annual, daily, weekly, etc.. precipitation/humidity/ temperature was measured  **Other climatic risk factor** includes degree-days.  Examples of **standing water** include puddles, water barrels and bird feeders. |
| If signs/symptoms is selected | | |
| Reported signs and symptoms of acute CHIKV infection.  (Format: +ve/N/time units and comments)  *(Check all that apply)* |  Fever, specify: ________   Joint pain/joint swelling/stiffness/ arthralgia/arthritis, specify : ________  ________   Rash, specify : ________   Muscle pain/myalgia, specify:________   Headache, specify: ________   Backache, specify: ________   Fatigue, specify: ________   Nausea/vomiting /diarrhea/abdominal pain: specify:________   Inflammation of eyes/conjunctivitis, specify: ________  Retro-orbital pain   Photophobia, specify : ______   Paralysis, specify: ________   Encephalopathy, specify : ________  Hemorrhagic  manifestations, specify: ____   Hyperpigmentation, specify: ____  Neurological symptoms, specify: ___   Cardiovascular/vascular symptoms, specify: : ____   Other, specify: ________ | When specifying, state the number of cases with symptom(s), number of total cases, duration of time with units and further comments. Order should be (#/#/time + units/comments)  If duration of illness in many patients is provided, state the range and mention that “individual patient data is available”. |
| How was CHIKV diagnosed in humans included in this study?  *(check all that apply)* |  Based on clinical symptoms, specify____  Virus isolation  RT-PCR (reverse transcription PCR)  rRT-PCR (real-time reverse transcription PCR)  Serology   Non-specified sero-test   IgG   IgM   Enzyme-linked immunosorbant assay (ELISA)   IgG   IgM   Direct/Indirect immunofluorescent-antibody assays (IFA)   IgG   IgM   Hemagglutinin/ inhibition assay (HA/HI)   Microneutralization tests (MNT)   Immunochromatographic tests (ICT)   Rapid detection tests (RDT), specify: ____   Plaque/Focus reduction neutralization tests (F/PRNT)   Loop-mediated isothermal amplification (LAMP)   Other, specify; ____  Molecular characterization   - Phylogeny - Mutations - Other    Other, specify: ______ | **Clinical symptoms:** Please list the symptoms that were used to diagnose one as being CHIKV infected in an alphabetical order. Example: [arthralgia, fever].  **RT-PCR:** Used to qualitatively detect gene expression through creation of complementary DNA transcripts from RNA  **rRT-PCR:** Used to quantitatively measure the amplification of DNA in real-time using fluorescent probes  **ELISA** is a laboratory technique used to measure concentration of antibodies/antigens in samples.  **Direct/Indirect Immunofluorescence (IF):** Use of antibodies to label and detect a specific target antigen with a fluorescent dye.  **Hemagglutinin or hemagglutinin inhibition assay:** Is a method for quantitating the relative concentration of viruses, or antibodies based on agglutination with red blood cells (RBCs)  **Microneutralization tests (MNT):** Assay used to determine whether a serum sample contains antibodies that block virus infection  **Immunochromatographic tests:** Also known as lateral flow tests or simply strip tests, are devices intended to detect presence/absence of antibodies/antigens in sample without use of heavy equipment  **Rapid diagnostic test:** Used to detect antigens present in blood of infected patients. Often used in areas where good quality microscopy services is not provided  **Plaque/Focus reduction neutralization tests** measure neutralizing antibodies for CHIKV  **Phylogeny:** Looking at relationships between different CHIKV genotypes. Dendogram is always provided.  **Mutations:** Authors specifically use mutation to identify CHIKV. Example alanine to valine mutation in the E1 envelope glycoprotein (E1-A226V genotype). |
| Persistent morbidity post-CHIK infection  (Format: +ve/N/time units and comments)  *(Check all that apply)* |  Arthritis   - Rhematoid, specify: ____ - Psoriatic, specify: _____ - Edematous polyarthralgia, specify: _____    Tendinitis, specify: _____   Synovitis, specify: _____   Fasciitis, specify: _____  Mechanical disbalance in joints/balance disorders, specify: _____   Tunnel syndromes, specify: _____   Guillaine-Barré syndrome, specify:________   Other, specify:____ | When specifying, state the number of cases with symptom(s), number of total cases, duration of time with units and further comments. Order should be (#/#/time + units/comments)  **Tendinitis** is inflammation of tendon.  **Synovitis** is inflammation of synovial membrane (membrane joins joints which possess cavities)  **Fasciitis** is inflammation of fascia (connective tissue surrounding muscles, blood vessels and nerves) of a muscle or organ. |
| If treatment is selected | | |
| What treatment options were used to treat CHIKV infections? |  Plant-based inhibitors, specify: _____   Non-steriodal anti-inflammatory drugs, specify: ______   Corticosteroids, specify: _________   Analgesics /anti-pyretic, specify: _________   Anti-viral drugs, specify: _________  Anti-malarials, specify: _________  Physical therapy or acupuncture, specify: _________  Traditional medicine, specify: ____   Other, specify: __________ | Specify name of drug. If the treatment option has only been studied in-vitro, specify “in-vitro treatment” in text box.  **Analgesics** is a group of drugs that is used to relieve pain. Examples include Tylenol, Parcetamol and Advil.  **Anti-pyretic drugs** are used to prevent/reduce fever.  **Anti-viral drugs** is a group of drugs used to treat viral infections by inhibiting development of virus.  **Traditional medicine** includes Chinese medicine, homeopathy and ayurvedic medicine. |
| What treatment options were evaluated for efficacy? |  Plant-based inhibitors, specify: _____   Non-steriodal anti-inflammatory drugs, specify: ____________   Corticosteroids, specify: _________   Analgesics /anti-pyretics, specify: _________   Anti-viral drugs, specify: _________  Anti-malarials, specify: _________  Physical therapy or acupuncture, specify: _________  Traditional medicine, specify: ____   Other, specify: __________ | Specify name of drug, and whether it was effective or non-effective as evaluated by author.  This question will be directed towards experimental studies. |
| If accuracy of diagnostic tests is selected | | |
| What tests were examined for their accuracy in the diagnosis of human cases and/or detection of CHIKV in non-human hosts?  *(Please check all that apply)* | - Clinical diagnosis (by signs and symptoms) - Virus culture and identification - Serological Tests    Enzyme-linked immunosorbant assay (ELISA)   IgG   IgM   Direct/Indirect immunofluorescent antibody assays (IFA)   IgG   IgM   Hemagglutinin/ inhibition assay (HI)   Microneutralization tests (MNT)   Immunochromatographic tests (ICT)   Rapid detection tests (RDT) specify: ___   Plaque/Focus reduction neautralization tests (F/PRNT)   Other serological test, specify: ___   - Molecular Tests    Virus detection   RT-PCR (reverse transcription PCR)   rRT-PCR (real-time reverse transcription PCR)   Nested PCR   Loop-mediated isothermal amplification (LAMP)   Other molecular tests, specify: __   - Other, specify:___ | **ELISA** is a laboratory technique used to measure concentration of antibodies/antigens in samples.  **Direct/Indirect Immunofluorescence (IF):** use of antibodies to label and detect a specific target antigen with a fluorescent dye.  **Hemagglutinin or hemagglutinin inhibition assay:** Is a method for quantitating the relative concentration of viruses, or antibodies based on agglutination with red blood cells (RBCs)  **Microneutralization tests (MNT):** Assay used to determine whether a serum sample contains antibodies that block virus infection  **Immunochromatographic tests:** Also known as lateral flow tests or simply strip tests, are devices intended to detect presence/absence of antibodies/antigens in sample without use of heavy equipment  **Rapid diagnostic test:** used to detect antigens present in blood of infected patients. Often used in areas where good quality microscopy services is not provided  **Plaque/Focus reduction neutralization tests** measure neutralizing antibodies for CHIKV  **RT-PCR:** Used to qualitatively detect gene expression through creation of complementary DNA transcripts from RNA  **rRT-PCR:** Used to quantitatively measure the amplification of DNA in real-time using fluorescent probes  **Nested PCR**: A modification of PCR intended to reduce non-specific binding in products due to the amplification of unexpected primer binding sites  **LAMP:** Amplification is carried out at a constant temperature, and does not require a thermal cycler unlike in PCR |
| Is information about sensitivity(SN), specificity (SP), and/or raw data provided? | - Yes   Information about diagnostic test (1-5)   - - Specify test:_____ - Specificity is provided - Sensitivity is provided - Raw data is available (for 2x 2 table) - Data on the agreement of this test compared to others is provided - Detection limits of test, specify: ____ - When should this test be used (e.g. x days after symptoms appear) :specify: ____ - Test is validated and ready to be used - Test is validated but not ready to be used, further research is recommended - Test is an experimental test that has not be validated - No, not provided | **Sensitivity** (also called the **true positive rate**) measures the proportion of positives that are correctly identified as such.  **Specificity** (also called the **true negative rate**) measures the proportion of negatives that are correctly identified as such.Eg., if 100 people known to have a disease were tested and 43 tested positive, the test has 43% sensitivity. If 100 people with no disease are tested and 96 return a negative result, then the test has **96%** specificity.  **Detection limits** – examples are cut off values for detecting positive or negative results for each test  This question is repeated seven times for seven separate diagnostic tests |
| Additional comments about diagnostic tests in this paper | **[text]** | Please capture any information you think is pertinent and has not been captured in the form. |
| If mitigation strategies is selected | | |
| What prevention/control strategies were investigated ?  *(Please check all that apply)* |  Vaccination, specify: ____   Vaccine is validated and ready to be used   Vaccine is validated but not ready to be used, further research is recommended   Vaccine tested in-vitro and has not been validated  Vaccination trial in: ___   Humans   Non-human primates   Mice   Other; specify___   Behavioural protective measures:   - - Wearing long pants and/or lightly-coloured clothing   - Tucking pants into socks   - Using repellents, specify ___   - Wearing clothing treated with permethrin insecticide   - Using mosquito/bed nets   Having window/door screens   - - Emptying standing water from containers such as flowerpots or buckets and cleaning them   - Removing/destroying vector habitats (e.g. containers/tires)   - Other behavioural measure, specify: _____   Use of **Insecticides**   - **Ovicide, specify: ______** - **Larvicide, specify: ______** - **Pupicide, specify: ______** - **Adulticide, specify: ________** - **Lethal ovitraps** - **Category not specified, but chemical name provided, ____**    Other chemical control measure_____   Biologic control of mosquitoes   - Sterile insect technique (SIT - Incompatible insect technique (IIT)/Cytoplasmic incompatability - Infection with an endosymbiotic bacterium - *Wolbachia,*  specify type: ___ - Other bacterium, specify: ___ - Release of Insects with Dominant Lethal [RIDL] mosquitoes - Use of larvivorous fish/copepods species; specify_____ - Other biological control of mosquito, specify: _____    Public education to decrease risk of CHIK disease, specify___   Other, specify ______ | **Specify** name and description in text box in the order, [name, description]  In the text box, include [commercial name, description]  **Ovicide** includes insecticides targeted at eggs. Dessicants would be placed here.  **Larvae** are hatched.  **Pupae** don’t have wings but can eat.  **Adults** are fully developed (have a head with two large compound eyes, a thorax, a pair of scaled wings, and six jointed legs). Adult mosquitoes mate within the first few days after emerging.  **Lethal ovitraps** or oviposition traps incorporate an insecticide on the oviposition substrate. These traps collect the eggs laid by mosquitoes.  Different types of **larvivorous fish** feed on immature stages of mosquitoes (e.g. *Gambusia affinis, Poecilia reticulate*), thereby controlling vector population. **Copepods** are small crustaceans found in both saltwater and freshwater environments. Predatory copepods consume mosquitoes.  **Sterile insect technique** involves releasing sterile mosquitoes into the environment to mate with native mosquito vectors. Insects are usually sterilized with irradiation.  **Incompatabile insect technique/ Cytoplasmic incompatability** results in egg and sperm being unable to form viable offspring. This is caused by changes in gametes due to intracellular parasites.  **Wolbachia** is a bacterium inducing male-killing, feminization, and cytoplasmic incompatibility  RIDL uses modern molecular biology techniques to insert lethal genes into insects. Sterile transgenic insects can then be used to control mosquitoes. |
| Did the authors describe the impact of the mitigation strategy? | Successes/positive impact, specify_____  Limitation/negative impact, specify _____ | Was the prevention/control method successful?  Insecticide resistance is a limitation. Discuss insecticide efficiency and resistance information here. |
| If social impact papers is selected | | |
| What public knowledge and attitudes and/or risk perceptions towards CHIK disease and potential prevention and control strategies did the paper investigate?  *(please check all that apply)* | □ Concerns about toxic or environmental effects of control measures (e.g. acaricides, DEET)  □ Perceptions about the severity of CHIK disease or vulnerabilities  □ Perceived efficacy of protective measures  □ Knowledge on behavioural mitigation practices  □ Knowledge on CHIK disease  □ Knowledge on CHIKV-harbouring vectors  □ Public attitudes towards paying for protection from CHIK disease (willingness to pay)  □ Other; specify: __________ |  |
| What specific populations were investigated for contextual information?  *(please check all that apply)* | □ General public  □ Physicians  □ Other medical or public health professionals, specify:________  □ Veterinarians  □ Government personnel, specify ____  □ NGO personnel, specify ___  □ Other, specify _____ | What populations did the researchers speak to? Gather information from? |
| How were the contextual data collected?  *(please check all that apply)* | □ Quantitative questionnaire or survey:  □ In-person, specify details _____  □ Phone, specify details _____  □ Postal questionnaire, specify details _____  □ Web-based questionnaire, specify details _____  □ Qualitative interviews:  □ In-person, specify details _____  □ Phone, specify details _____  □ Postal questionnaire, specify details _____  □ Web-based questionnaire, specify details _____  □ Focus groups  □ Analysis of documents, specify details ____  □ Other, please specify ______  □ Not specified |  |
| Was the contextual data collection informed by one or more theories of human behaviour? |  Yes, Health Belief Model   Yes, Stages of Change Theory   Yes, Theory of Planned Behaviour   Other, specify _________   No | **Health Belief Model**: Authors report that the intervention changed the participants' self-efficacy and/or perceived barriers/threats to changing their behaviour  **Stages of Change Theory**, Authors report how the intervention changed the participants’ reported/observed “stages of change” classification.  **Theory of Planned Behaviour**: Did the study identify a preconceived attitude, subjective norm, and/or perceived behaviour control among participants that was addressed by the intervention, which in turn altered their behaviour? |
| If economic burden is selected | | |
| Does the article report on the economic burden of CHIK disease or cost-benefit of control measures?  *(Check all that apply)* |  Yes, economic burden   Descriptive, specify: ____ _______   Yes, specific cost estimates/numbers, please copy details _____   Yes, cost-benefit of control measures   Descriptive, specify type _______   Yes, specific cost estimates/numbers: please copy details _________   No |  |
| If CHIK vector is selected | | |
| What method(s) were used to trap and/or observe the vectors?  *(Check all that apply)* | Traps   - Ovitrap - Ovitrap with odour-based attractant, please specify: __ - Ovitrap with other attractant, please specify: ___ - Ovitrap without bait - Other trap, specify: ___ - Trap with odour-based attractant, please specify: ____ - Trap with other attractant, please specify: ___ - Trap without bait   Baits only   - Odour-based attractant, please specify: ___ - Other attractant, please specify: ___ - Human bait    Other: _____  Trapping and/or baiting occurred, but method not specified/reported   Laboratory-reared |  |
| What test was used to identify CHIKV in mosquitoes?  *(Check all that apply)* | - Loop-mediated isothermal amplification (LAMP) - Virus culture/identification/quantification - Mice inoculation/titration/observation - Serological tests - ELISA - EIA - Neutralization tests - Complement fixation - IFA - Plaque assay - HI - Unspecified - Molecular tests - RT-PCR - Rrt-pcr - Nested pcr - Sequence Analysis - Other, specify:___ |  |
| Did the study examine vector competence, biology and/or transmission?  (Only answer this question if there is some data/ range / measurement for the outcome specified.)  *(Check all that apply)* |  Yes, measured in species:   - Aedes aegypti - Aedes albopictus - Other CHIK vector, specify: ____   Yes, competence of CHIKV vectors   - Adult longevity/lifespan in days, please specify:______ - Temperature, specify: ___ - Humidity, specify: ___ - Precipitation, specify: ___ - Mosquito density per human, please specify: _____ - Temperature, specify: ____ - Humidity, specify: ____ - Precipitation, specify: ____ - Range of mosquito habitats, specify: ______ - Temperature, specify: ___ - Humidity, specify: ___ - Precipitation, specify: ___ - Egg diapause, please specify: _____ - Temperature, specify: ___ - Humidity, specify: ___ - Precipitation, specify: ___ - Female fecundity rate, please specify:____ - Temperature, specify: ___ - Humidity, specify: ___ - Precipitation, specify: ____ - Egg hatching rate, please specify: ____ - Temperature, specify: __ - Humidity, specify: ____ - Precipitation, specify: ____ - Extrinsic incubation period, please specify in days: _____ - Temperature, specify: ____ - Humidity, specify: ____ - Precipitation, specify: ___ - Proportion of mosquitoes surviving the EIP - Temperature, specify: ____ - Humidity, specify: ____ - Precipitation, specify: ____ - Time for emergence to next stage - Larvae, specify:___ - Temperature, specify: ___ - Humidity, specify: ___ - Precipitation, specify: ___ - Pupae, specify: ___ - Temperature, specify: ___ - Humidity, specify: ___ - Precipitation, specify: ___ - Adults, specify: ____ - Temperature, specify: __ - Humidity, specify: ___ - Precipitation, specify: ___ - Infection rate _____ - Transmission rate ____ - Dissemination rate _____    Yes, vector behaviour   - Feeding behaviour, specify ____ - Host biting - Time of biting activity (night or day): ___ - Biting rate _____ - Indices - Breteau index: ____ - Container index: ____ - House index: ____ - Other: _____ - Other; specify____    Yes, transmission of virus  Human/host to mosquito transmissibility   - Transmission rate: ____   Mosquito-to-human/host transmissibility   - Transmission rate: ____   Vertical transmission: ____  Sexual/venereal transmission: ____  Other: ____ | **Competence of CHIKV vectors:** Characteristics of vector that allow it to transmit CHIKV. This includes mosquito lifespan, female fecundity rate and extrinsic incubation period. If provided, specify the temperature, humidity and precipitation range for the different competence factors.  Specify **lifespan** in days. Eg. 4 days  **Mosquito density per human** is the number of mosquitoes per human in the population being modelled.  Specify density. Eg. 100 mosquito/human  Specify **range**. Eg. 10 km  **Egg diapause** is a physiological state of dormancy in which development is delayed. It is a mechanism used by eggs to survive unfavourable environmental conditions. Specify duration of diapause. Eg. 1 month  Specify female **fecundity rate** (number of eggs laid per female mosquito)  Specify **hatching rate:** The time needed for eggs to hatch  **Extrinsic incubation period** is the time required for a pathogen to spread from the mosquito’s gut where the virus is first present to the salivary glands where the virus can be subsequently transmitted.  Infection/dissemination/transmission rates are defined differently by authors. Please provide definition as outlined by author. These parameters are often provided in experimental studies where mosquitoes are inoculated with CHIKV.  **Feeding behaviour** can include information on where the mosquito prefers to bite (e.g. ankle)., if the species prefers humans to animals or if they have multiple blood feeds per feeding session.  **Time of biting activity** is whether day or nocturnal biting pattern  **Biting Rate** is the number of bites on a human, per mosquito, within a given time period.  **Human-to-mosquito transmissibility** is the probability of a mosquito acquiring CHIKV from an infectious human/host during a single blood meal.  **Mosquito to human/host transmissibility** is the probability of a human/host acquiring CHIKV from an infected mosquito during a single blood meal |
| Did the researchers study the phylogeny of Aedes mosquitoes? | - Yes, phylogeny of *Aedes aegypti* studied - Yes, phylogeny of *Aedes albopictus* studied - No | Phylogeny: Evolutionary history of Aedes mosquitoes. Dendrogram will be provided. |
| What **mosquito exposure/abundance** risk factors were investigated (ie: sampled and tested, not just mentioned)?  (Only applies to epidemiology studies: surveys, cross sectional, case control, cohort. Not outbreak investigations)  *(Please check all that apply)* | **Geographic**   Coastal area   - Statistically significant risk factor, protective - Statistically significant risk factor, increased risk - Not statistically significant    Inland   - Statistically significant risk factor, protective - Statistically significant risk factor, increased risk - Not statistically significant   Urban   - Statistically significant risk factor, protective - Statistically significant risk factor, increased risk - Not statistically significant   Suburban or peri-urban   - Statistically significant risk factor, protective - Statistically significant risk factor, increased risk - Not statistically significant   Rural   - Statistically significant risk factor, protective - Statistically significant risk factor, increased risk - Not statistically significant    Private dwelling   - Statistically significant risk factor, protective - Statistically significant risk factor, increased risk - Not statistically significant    Private garden   - Statistically significant risk factor, protective - Statistically significant risk factor, increased risk - Not statistically significant    Park   - Statistically significant risk factor, protective - Statistically significant risk factor, increased risk - Not statistically significant   Cemetery   - Statistically significant risk factor, protective - Statistically significant risk factor, increased risk - Not statistically significant    Swimming pool   - Statistically significant risk factor, protective - Statistically significant risk factor, increased risk - Not statistically significant   Salt-water beach   - Statistically significant risk factor, protective - Statistically significant risk factor, increased risk - Not statistically significant   Fresh-water beach   - Statistically significant risk factor, protective - Statistically significant risk factor, increased risk - Not statistically significant    Farm/agricultural landscape   - Statistically significant risk factor, protective - Statistically significant risk factor, increased risk - Not statistically significant    Forest   - Statistically significant risk factor, protective - Statistically significant risk factor, increased risk - Not statistically significant    Transportation of goods by land: ___   - Statistically significant risk factor, protective - Statistically significant risk factor, increased risk - Not statistically significant   Transportation of goods by water: ___   - Statistically significant risk factor, protective - Statistically significant risk factor, increased risk - Not statistically significant   Transportation of goods by air: ___   - Statistically significant risk factor, protective - Statistically significant risk factor, increased risk - Not statistically significant   Other geogprahic: ___   - Statistically significant risk factor, protective - Statistically significant risk factor, increased risk - Not statistically significant    Latitude/Longitude   - Statistically significant risk factor, protective - Statistically significant risk factor, increased risk - Not statistically significant    Elevation/altitude   - Statistically significant risk factor, protective - Statistically significant risk factor, increased risk - Not statistically significant   **Climate**   Precipitation, specify: ___   - Statistically significant risk factor, protective - Statistically significant risk factor, increased risk - Not statistically significant    Season, specify:   - Statistically significant risk factor, protective - Statistically significant risk factor, increased risk - Not statistically significant    Relative humidity, specify: ____   - Statistically significant risk factor, protective - Statistically significant risk factor, increased risk - Not statistically significant    Atmospheric moisture, specify:____   - Statistically significant risk factor, protective - Statistically significant risk factor, increased risk - Not statistically significant    Minimum humidity, specify: ____   - Statistically significant risk factor, protective - Statistically significant risk factor, increased risk - Not statistically significant    Maximum humidity, specify: ____   - Statistically significant risk factor, protective - Statistically significant risk factor, increased risk - Not statistically significant    Temperature, specify: ____   - Statistically significant risk factor, protective - Statistically significant risk factor, increased risk - Not statistically significant    Minimum temperature, specify: ____   - Statistically significant risk factor, protective - Statistically significant risk factor, increased risk - Not statistically significant    Maximum temperature, specify: ___   - Statistically significant risk factor, protective - Statistically significant risk factor, increased risk - Not statistically significant    Mean temperature, specify: ___   - Statistically significant risk factor, protective - Statistically significant risk factor, increased risk - Not statistically significant    Maximum temperature of warmest month   - Statistically significant risk factor, protective - Statistically significant risk factor, increased risk - Not statistically significant    Minimum temperature of coldest month   - Statistically significant risk factor, protective - Statistically significant risk factor, increased risk - Not statistically significant    Other climatic risk factor, specify: ____   - Statistically significant risk factor, protective - Statistically significant risk factor, increased risk - Not statistically significant   **Human behaviours**   Occupational risk; please specify__   - Statistically significant risk factor, protective - Statistically significant risk factor, increased risk - Not statistically significant    Outdoor recreational activities (e.g. picnics, camping) ; please specify__   - Statistically significant risk factor, protective - Statistically significant risk factor, increased risk - Not statistically significant    Walking or jogging   - Statistically significant risk factor, protective - Statistically significant risk factor, increased risk - Not statistically significant    Swimming   - Statistically significant risk factor, protective - Statistically significant risk factor, increased risk - Not statistically significant    Gardening or yard work   - Statistically significant risk factor, protective - Statistically significant risk factor, increased risk - Not statistically significant    Storage of unused tires   - Statistically significant risk factor, protective - Statistically significant risk factor, increased risk - Not statistically significant    Emptying containers and pots   - Statistically significant risk factor, protective - Statistically significant risk factor, increased risk - Not statistically significant    Maintenance of standing water on property, please state____   - Statistically significant risk factor, protective - Statistically significant risk factor, increased risk - Not statistically significant    Travel related   - Statistically significant risk factor, protective - Statistically significant risk factor, increased risk - Not statistically significant    Other please specify ____   - Statistically significant risk factor, protective - Statistically significant risk factor, increased risk - Not statistically significant | **Coastal area**: Area where land meets the sea/ocean  **Urban:** Location characterized by high human density and significant development (high density of human structures such as houses, commercial buildings, roads, bridges, etc.)  **Suburban:** An area located on the outskirts of a city/developed area. It is usually a residential area. Peri-urban is an area immediately adjoining an urban area. It’s an area where you have a mixture of urban and rural activities. The term peri-urban is commonly used in Europe, India and Africa.  **Rural:** Area where you have very few homes and very low population density.  **Swimming/wading pool** is man-made. It can be a container that is filled with water. Examples include public pools and ocean pools.  **Beach** is a landform/strip along the coast of the ocean or sea (salt-water) or edge of a lake or river (fresh-water)  **Transportation of goods by land** includes trucks carrying loads of cargo such as bamboo sticks. **Transportation of goods by water** include carrying cargo in river boats, ships, etc. **Air transport** includes airplanes carrying goods/passengers.  **Other land use** includes industrial area and warehouses.  Specify whether annual, daily, weekly, etc.. precipitation/humidity/ temperature was measured  **Other climatic risk factor** includes degree-days. |
| If Predictive Models is selected | | |
| Please indicate species modelled  *(check all that apply)* | - Humans - Non-human mammals, specify: _____ - Birds, specify: _____ - Reptiles, specify:_____ - Arthropods, specify:_____ - No species |  |
| Please indicate how the model incorporates (or not) time and space | - Both time and space are implemented in the model (spatially distributed model) - Only time is implemented (not spatially distributed model) - Only space is implemented - No time dimension (not a dynamic model over time) - Other, specify:_____ | **Both time and space are implemented in the model (Spatially distributed model):** A system for which the simulated dependent variable is a function of time and space. It implies that at least some of the parameters/state variables vary over space in addition to time during a simulation.  **Only time is implemented (not spatially distributed model):** the dependent variable is only function of time and initial value of state variables.  **No time dimension** explicit, the model is not a “dynamic” model over time (ex.: some network models |
| Please describe the class of model used/presented | - Stock and flow (system dynamics) - Agent based or cellular automata - Discrete event model - Network model - Generalized linear model (e.g. linear regression, ANOVA, logistic) - To be determined | **Stock and flow:** the dynamic behaviour of the model is driven by flows that accumulate in stocks. (e.g. SI, SIR, SEIR, which are example of state transition models). System dynamics model are usually simulated using a constant time step (dt) and a seldom spatially distributed.  **Agent based model** (also referred to as microsimulation): simulate the actions and interactions of autonomous agents with their neighbours and their environment based on sets of rules. There may be more than one type of agents. (note: similarly to Stock and flow, agent based can use the state transmission approach). This kind of model may or may not be spatially distributed. Cellular automata show similarities with agent based model but exist within a discrete space (a grid for example)  **Discrete event model:** This is a simulation system where the state variable changes according to specific events instead of over regular time steps or continuous time flow (ie. there is no fixed time-step at which point the state of variables are updated). (Note: they are usually used when the element of interest is related to specific event and as such are more often used by engineer to simulate industrial assembly for example).  **Network model:** A network database consists of a collection of records connected to one another through links. Note: I am pretty confident that if a network model is used, the word “network” will be explicit in the abstract, or even in the title. |
| Please indicate to what country the model applies to  *Specify country* | - North America (Canada, USA, Mexico), specify:_____ - Central America/South America/Caribbean, specify:_____ - Africa (includes Indian Ocean Islands), specify:_____ - Europe (except Russia), specify: _____ - Russia - Asia (includes Middle East, Turkey, Japan), specify:_____ - Australasia and New Zealand, specify:_____ - Oceania (includes Fiji, Micronesia, Polynesia, and other Pacific island countries), specify: ­­­­­_____ - Reunion Island or unknown continent, specify:_____ - Not reported or “generic transmission model” |  |
| Please describe if the model is used to:  *Check all that apply* | - Predict and outcome such as the number of cases (sick or infected), and incidence rate, or a prevalence rate - Economic assessments (cost-benefits, costs, etc.) - New knowledge/understanding of disease transmission/epidemic spread (according to the authors) - Evaluation of mitigation measures or programs - Other, specify:_____ |  |
| Please indicate if calibration was performed | - Yes - No - Not reported - To be determined | **Calibration** refers to:   - **Empirical calibration:** parameter values based on empirical data belonging to the system being simulated - “**Literature” calibration:** the parameter values obtained from literature |
| Please indicate if a validation is performed | - Yes - No - Not reported - To be determined | **Validation performed:** the validity of the model is assessed by the authors (by one of the means below):   - **Empirical validation:** the output of the model is assessed against empirical data. - **Logical validation**: the authors verify that the model behaviour is coherent with the assumptions made or the knowledge of the system (sometimes included in the “verification” step) |
| Please indicate if the model is stochastic or deterministic | - Stochastic model - Deterministic model - Not reported - To be determined | **Stochastic model:** the model contains inherent randomness and the model output is different for each run resulting in an ensemble of different outputs across multiple runs  **Deterministic model:** the model output is determined by the parameters and the initial conditions set in the model and the outputs are identical across multiple runs |
| Please indicate which of the following elements are presented in the article or in the appendix/supplementary material  *Check all that apply* | - Model presentation (text or schematic) - Simulation results - Sensitivity analysis - Assessment/simulation of mitigation methods or management scenarios - To be determined | **Sensitivity analysis:** different ways by which “the study of how uncertainty in the output of a model can be attributed to different sources of uncertainty in the model input”. (Saltelli et al 2008). |
| Please indicate if the simulation was performed in a commercially available or open source software  *Check all that apply* | - Spreadsheet - Specialized software, specify: ______ - Not reported |  |
| Please indicate if the model’s equations are presented explicitly in the article or in the appendix  *Check all that apply* | - Yes, in the main article - Yes, in the appendix - No |  |
| Additional comments | **[text]** | Include any information here that was not captured earlier on in the form |

## Appendix 4: Search Strategy Implemented

### Databases

|  | Number of Hits | |
| --- | --- | --- |
| Database | 1^st^ search: May 27 2015 | 2^nd^ search: Jan 6 2017 |
| Pubmed | 2939 | 968 |
| Scopus | 3133 | 1222 |
| CAB | 2662 | 771 |
| Agricola | 191 | 75 |
| CINAHL | 152 | 177 |
| Cochrane | 13 | 7 |
| Embase | 133 | 1715 |
| LILACS | 33 | 60 |
| Total | 12411 | 4995 |

### Grey Literature

Searches conducted on: October 19 -23, 2015 and January 6, 2017.

Grey Literature Searched:

- WHO Library (SEARO)
- IMSEAR
- IMEMR
- The World Bank
- CDC (MMWR, FastStats)
- Arbonet
- PHAC
- PHO
- National Institutes of Health Canada - Santé Canada
- Australia’s National Notifiable Disease Surveillance System (NNDSS)
- European Centre for Disease Prevention and Control (ECDC)
- ENHanCEd Infectious Diseases (EID2 database)
- Communicable Disease Intelligence – Australian government Department of Health
- PAHO
- Eurosurveillance
- Global Health Database
- Promed-mail
- Infochangeindia.org
- Asia Development Bank
- IndMED
- MedCarib
- Caribbean Public Health Agency (www.carpha.org)

# of articles included: 22

### Search Verification

Search conducted on: October 14, 2015

Articles searched to hand pick references for search verification:

1. Mowatt, L., & Jackson, S.T. (2014). Chikungunya in the Caribbean: An Epidemic in the Making. Infect Dis Ther, 3:63-68.
2. Roques, P., & Gras, G. (2010). Chikungunya Fever: Focus on Peripheral Markers of Pathogenesis. The Journal of Infectious Diseases, 203:141-143.
3. Higgs, S., & Ziegler, S.A. (2010). A nonhuman primate model of Chikungunya disease. J Clin Invest, 120(3): 657-660.
4. Ogden, N.H., Lindsay, L.R., & Coulthart, M. (2015). Is there a risk of Chikungunya transmission in Canada? CCDR, 41-1.
5. Straetemans, M. (2008). Vector-related risk mapping of the introduction and establishment of Aedes Albopictus in Europe. Eurosurveillance, 13(1-3).
6. Weitzel, B.S., Rega, P.P., & Bork, C.E. (2012). Chikungunya virus: An emerging condition in the industrialized world. JAAPA, 25(1):E1-E5.
7. Parida, M.M., Santosh, S.R., Dash, P.K., & Rao, P.V.L. (2008). Rapid and real-time assays for detection and quantification of Chikungunya virus. Future Medicine, 3(2): 179-192.
8. Kar, P.K., Nagpal, B.N., Dua, V.K., Ghosh, S.K., Raghavendra, K., Bhatt, R.M., Anvikar, A., & Das, A. (2009). Molecular characterization of chikungunya virus from Andhra Pradesh, India. Indian J Med Res, 129: 335-337.
9. Cassadou, S., Boucau, S., Petit-Sinturel, M., Huc, P., Leparc-Goffart, I., & Ledrans, M. (2014). Emergence of chikungunya fever on the French side of Saint Martin island, October to December 2013. Euro Surveill. Available online: <http://www.eurosurveillance.org/ViewArticle.aspx?ArticleId=20752>
10. Kaur, P., & Chu, J.H. (2013). Chikungunya virus: an update on antiviral development and challenges. Drug Discovery Today, 18 (19/20):969-983.

# of articles included: 8 from searching above list

Total number of articles added:

Eight from hand searches using reference lists of 10 articles

22 articles grey literature
