# Supplementary material for: A scoping review of published literature on chikungunya virus
Source: PLoS One. 2018 Nov 29;13(11):e0207554. doi: 10.1371/journal.pone.0207554 (PMC6264817; doi:10.1371/journal.pone.0207554)
Supplement: S1 Expert Questionnaire — (DOCX) [file pone.0207554.s003.docx]

*S1 Expert Questionnaire: Scoping review of the global knowledge on Chikungunya virus: prevalence, spread, prevention and control strategies, risk factors and diagnostics*

# *What is the purpose of this study?*

The Laboratory for Foodborne Zoonoses at the Public Health Agency of Canada has prioritized to conduct a scoping review on Chikungunya in 2015-2016

More than 1.2 million suspected chikungunya (CHIK) cases have been reported from the Americas alone within the last two years (Ref PAHO), highlighting the rapid emergence of this pathogen in previously uninhabited areas. Due to the increasing threat of geographical expansion, the chikungunya virus (CHIKV) is an increasingly important public health concern and requires constant monitoring and vigilance by public health authorities to decrease risk to susceptible populations. While there is a WHO global strategy for 2012-2020 to prevent and control dengue, there has not been a similar strategy identified for CHIK to date (Ref – WHO). In addition, there is ambiguity surrounding best practices for surveillance, detection, prevention and control, and other mitigation strategies for CHIKV.

Conducting a scoping review of the available literature on CHIKV infections will enable us to compile and analyse relevant information pertinent to prevention and control strategies for mitigating the introduction and development of this disease in Canada. Effective and efficient strategies can be identified and implemented in a timely manner with this collated and readily available information. There is a need to systematically summarize the current state of knowledge regarding CHIKV surveillance and potential prevention and control measures to support evidence-informed decision-making that ensures enhanced surveillance and effective education activities to address this emerging public health issue. A comprehensive review of current knowledge can also highlight areas where future field and experimental work is needed to fill existing knowledge gaps associated with CHIK disease.

## Planned Study Outputs

1. Primary scoping study of the relevant published and grey literature on the topic
2. A summary of findings fact sheet.
3. A repository and dataset of all relevant literature captured in this study.

## *What are the research questions of interest?*

## Study Question

*What is the current state of research knowledge on chikungunya infections in humans, mosquito vectors and vertebrate reservoirs?*

*A summary of surveillance methods, prevention and control strategies, transmission, detection and burden of illness, risk factors, and knowledge, societal attitudes and perceptions.*

## Study Sub- Questions

1. Surveillance methods for CHIKV harbouring mosquitoes and CHIKV infections [vectors, animal reservoirs, humans]
2. Available diagnostic methods for humans; feasibility, validity and reliability
3. Short and long term sequelae for humans and treatment efficacy
4. Burden of illness; incidence/ prevalence
5. The effectiveness of prevention/intervention/control/education programs or strategies [vectors, animal reservoirs, humans]
6. Risk factors for CHIKV in animals/vectors and CHIK infections in humans
7. Societal attitudes and perceptions towards CHIK infection prevention and control strategies
8. Transmission of CHIKV

# *How will the review be conducted?*

The review will follow standard scoping review methodology consisting of the following steps: a comprehensive search strategy, screening of identified abstracts for relevance, extracting key characteristics and information from relevant articles, analyzing, summarizing and reporting the results to key knowledge users. PRISMA (Preferred Reporting Items for Systematic Reviews and Meta-analyses) guidelines will be adhered to for reporting.

***Data Analysis***

This will likely be a descriptive tabulation of all pertinent information regarding CHIKV; including risk factors, surveillance, diagnostics, prevention and control, and perceptions and attitudes. Findings and recommendations, methods, and study limitations will also be captured. Study findings will highlight areas with considerable research and where knowledge gaps and areas for future research exist.

# *What can I contribute to this study as a member of the stakeholder committee?*

At this stage of the review, we are asking for your comments and feedback on the components of the review (with detailed questions in the sections below):

1. Review scope and eligibility criteria
2. Search strategy
3. Key characteristics for data extraction

The review scope and eligibility criteria are listed below:

|  | **Inclusion criteria** | **Exclusion criteria** |
| --- | --- | --- |
| *Topic* | - CHIKV; current state of research knowledge - Articles from all countries will be included | - Exclude if focus is not on CHIK infections or CHIKV |
| *Population* | - Vectors and hosts from all species, including humans, with exposure or at risk of CHIKV infection - Confirmed and potential/probable cases - Vulnerable/susceptible populations | - Hosts used in experimental studies |
| *Research Domains* | - **Surveillance methods** for CHIKV in vectors, animal or human hosts; i.e., disease surveillance systems that detect and/or monitor trends of the presence or absence of a disease, vector surveillance systems that detect and/or monitor the presence of existing or potential mosquitoes species (not limited to Aedes aegypti and Aedes albopictus) which are involved in the transmission of CHIKV - **Diagnostic tests**; efficiency in terms of sensitivity and specificity, validity and reliability for humans - Prevention, control, or education strategies associated with mitigating CHIKV (interventions) - Epidemic preparedness, response and control - **Risk factors** for CHIKV acquisition in vectors, animals or humans - Societal **attitudes or perceptions** towards CHIKV infections - Studies describing short and long term **sequelae** (humans only) resulting from CHIK infections and treatment - **Burden of illness** studies - **Economic** impact from CHIK infections - **Transmission** of CHIKV | - Exclude if the study is on the morphology of CHIKV - Exclude if the study utilizes animal models or *in vitro* systems to decipher a molecular, immune system or other pathway not within the scope of the review question. |
| *Pathogen* | - CHIKV, all CHIKV genotypes will be captured, including West African, East/Central/South African (ECSA) and Asian genotypes | - Non CHIKV pathogens |
| *Outcome* | - Primary scoping study of the relevant published and grey literature on the topic - A summary of findings fact sheet with significant outcomes related to the review question - A repository and dataset of all relevant literature captured in this study | - N/A |
| *Study design* | - All study designs; descriptive, observational and experimental. Randomized and non-randomized controlled trials, quasi-experimental, before and after studies, prospective and retrospective cohort studies, case-control studies, analytical cross-sectional studies, descriptive cross-sectional studies, case series, individual case reports, and spatial, GIS and risk modelling will all be included. | - Experimental animal studies, *in-vitro* models or challenge studies to identify molecular pathways or mechanisms in CHIKV, vectors or hosts |
| *Publication language* | - English or French | - Any language other than English or French |
| *Publication date* | - No time limits on literature capture | - N/A |
| *Publication type* | - All primary research including non-peer-reviewed credible sources* (e.g. journal articles, government and research reports*, program evaluations, dissertations and theses*, and conference abstracts and papers*) | - None |

### Inclusion / Exclusion criteria

1. Time frame – no time frame?
2. Country – All?
3. Language – English and French only
4. Document type: All - any peer review primary articles, reviews, commentaries, PhD/MSc Theses

**Questions:**

**Do you have any comments or feedback on the review scope and eligibility criteria as noted above?**

***Search strategy:***

A comprehensive search strategy will be developed to guide the review with the primary aim of finding all studies and grey literature that are published within the scope of the review question. Keywords and index terms will be utilized to search various databases and websites that are outlined below.

We are planning to search the following bibliographic databases:

- Scopus, PubMed/MEDLINE, Embase, CINAHL (Cumulative Index to Nursing & Allied Health), ProQuest Public Health, CAB, LILACS (South American)
- Cochrane

Possible sources of grey literature

- WHO library (including SEARO, IMSEAR, IMEMR)
- The World Bank (*databank.worldbank.org/data/databases/infectious-diseases*
- CDC – MMWR, FastStats – Infectious Disease ( [www.cdc.gov/nchs/faststats/infectious -disease.htm](http://www.cdc.gov/nchs/faststats/infectious%20-disease.htm), ArboNET
- PHAC
- National Institutes of Health (NIH)
- Australia’s **National Notifiable Disease Surveillance System (NNDSS)**
- European Centre for Disease Prevention and Control (ECDC)
- **ENHanCEd Infectious Diseases (EID2 database); www.zoonosis.ac.uk/eid2**
- **Communicable Diseases Intelligence – Australian government’s Department of Health**
- PAHO
- Eurosurveillance
- Global Health Database (<http://www.lshtm.ac.uk/library/resources/databases/info_globalhealthovid.html>)
- ProMED-mail
- Infochangeindia.org
- Asia Development Bank
- IndMED
- MedCarib
- Conferences (We will be looking at the most recent meetings from 2013 onwards to collect new, likely unpublished research
  - ESCAIDE
  - CDC EIS Conference
  - ICEID
  - TEPHINET

Due to the nature of the scope of this study, we are hoping to capture all data relating to CHIKV that is relevant to the review question. The proposed search terms are therefore broad enough to be all-encompassing.

| **Category** | **Terms** |
| --- | --- |
| Topic | Chikungunya OR CHIK OR CHIKV |
| Publication date | No time limits. All articles will be screened. |
| Proposed final search string (Yields 2654 hits on April 15, 2015) | Search (Chikungunya OR CHIK OR CHIKV) |

NOTE: All search terms and synonyms are combined using the ‘OR’ operator

**Questions:**

- **Are you aware of any other sources, repositories or databases we should search to identify relevant articles or studies?**
- **Are you aware of any other important search terms we should consider including in the search algorithm?**
- **Do you have any suggested organizations or websites we should search for “grey literature”?**
- **Do you have any other comments or suggestions on the search strategy?**

***Key characteristics for data extraction:***

The characteristics and data we plan to extract from each relevant article are summarized in the table below:

| **Domain** | **Variables for extraction** |
| --- | --- |
| *Article characteristics* | - *Publication date* - *Document type (e.g.* journal article, conference proceedings, research report, other) - *Article language (e.g.* English, French) |
| *Study characteristics* | - *Location of study (*continent and country) - *Study design* (examples provided above) - *Surveillance type (active, passive or both); disease and vector surveillance, syndromic surveillance* - *Epidemic preparedness, response and control* - *Location of cases with infections (e.g*. hospitals, nursing homes, family homes, etc.) - *CHIKV genotype* - *Detection method used with sensitivity and specificity of test* - *Transmission details(e.g., travel related or autochthonous)* - *Vehicle of transmission (e.g., Aedes or other mosquito species)* - *Sylvatic/domestic hosts* - *Mortality and morbidity; numbers and details* - *Vulnerable populations* - *Demographic information*    - Will include extraction of detailed demographic information about the populations (*e.g*. age, sex/gender, ethnicity, socio-economic status, education level, rural/urban living, targeted behaviour) - Educational tools and their effectiveness |
| *Outcomes* | - Available literature pertinent to review question scope Categorised into sub-question categories and evaluated by geography, geno-type, timeframe of research and other study attributes (e.g. test, risk factor, intervention.) - Other available outcomes (e.g. utility and cost-benefit) - Study limitations - Highlighting gaps for future research - Recommendations from authors |

**Questions:**

- **Are there specific characteristics or information we should make sure is extracted because it will help us properly group studies and/or understand differences between studies ?**
- **Do you have any other comments or feedback on the proposed characteristics for data extraction as noted in the table above?**
