# Supplementary material for: A scoping review of published literature on chikungunya virus
Source: PLoS One. 2018 Nov 29;13(11):e0207554. doi: 10.1371/journal.pone.0207554 (PMC6264817; doi:10.1371/journal.pone.0207554)
Supplement: S1 Table — (DOCX) [file pone.0207554.s004.docx]

| **S1 Table: List of CHIKV genetic variants associated with evolution and fitness** | | | | | | | | | | | | | | | | | | | | | |  |
| --- | --- | --- | --- | --- | --- | --- | --- | --- | --- | --- | --- | --- | --- | --- | --- | --- | --- | --- | --- | --- | --- | --- |
|  |  | |  |  |  | |  | |  | |  | |  |  |  | |  |  |  |  | |  |
| *Structural proteins* | | | | | | | | | | | | | | | | | | | | | |  |
|  |  | |  |  |  | |  | |  | |  | |  |  |  | |  |  |  |  | |  |
| E1 | | | | **E2** | | | | | **E3** | | | | | **Capsid** |  | |  | **6K** | | | |  |
| *Nucleotide* | *Amino acid* | | *Refid* | *Nucleotide* | *Amino acid* | | *Refid* | | *Nucleotide* | | *Amino acid* | | *Refid* | *Nucleotide* | *Amino acid* | | *Refid* | *Nucleotide* | *Amino acid* | | *Refid* | |
|  | **A226V / V266A** | |  |  |  | |  | |  | |  | |  |  |  | |  |  |  | ([1](#_ENREF_1)); ([2](#_ENREF_2)); ([3](#_ENREF_3)); ([4](#_ENREF_4)); ([5](#_ENREF_5)); ([6](#_ENREF_6)); ([7](#_ENREF_7)); ([8](#_ENREF_8)); ([9](#_ENREF_9)); ([10](#_ENREF_10)); ([11](#_ENREF_11)); ([12](#_ENREF_12)); ([13](#_ENREF_13)); ([14](#_ENREF_14)); ([15](#_ENREF_15)); ([16](#_ENREF_16)); ([17](#_ENREF_17)); ([18](#_ENREF_18)); ([19](#_ENREF_19)); ([20](#_ENREF_20)); ([21](#_ENREF_21)); ([22](#_ENREF_22)); ([23](#_ENREF_23)); ([24](#_ENREF_24)); ([25](#_ENREF_25)); ([26](#_ENREF_26)); ([27](#_ENREF_27)); ([28](#_ENREF_28)); ([29](#_ENREF_29)); ([30](#_ENREF_30)); ([31](#_ENREF_31)); ([32](#_ENREF_32)); ([33](#_ENREF_33)); ([34](#_ENREF_34)); ([35](#_ENREF_35)); ([36](#_ENREF_36)); ([37](#_ENREF_37)); ([38](#_ENREF_38)); ([39](#_ENREF_39)); ([40](#_ENREF_40)); {12723}; ([41](#_ENREF_41)); ([42](#_ENREF_42)); ([43](#_ENREF_43)); ([44](#_ENREF_44)); ([45](#_ENREF_45)); ([46](#_ENREF_46)); ([47](#_ENREF_47)); ([48](#_ENREF_48)); ([49](#_ENREF_49)); ([50](#_ENREF_50)); ([51](#_ENREF_51)); ([52](#_ENREF_52)); ([53](#_ENREF_53)); ([54](#_ENREF_54)); ([55](#_ENREF_55)); ([56](#_ENREF_56)); ([57](#_ENREF_57)); ([58](#_ENREF_58)); ([59](#_ENREF_59)); ([60](#_ENREF_60)); ([61](#_ENREF_61)); ([62](#_ENREF_62)); ([63](#_ENREF_63)); ([64](#_ENREF_64)); ([65](#_ENREF_65)); ([66](#_ENREF_66)); ([67](#_ENREF_67)); ([68](#_ENREF_68)); ([69](#_ENREF_69)); ([70](#_ENREF_70)); ([71](#_ENREF_71)); ([72](#_ENREF_72)); ([73](#_ENREF_73)); ([74](#_ENREF_74)); ([75](#_ENREF_75)); ([76](#_ENREF_76)); ([77](#_ENREF_77)); ([78](#_ENREF_78)); ([79](#_ENREF_79)); ([80](#_ENREF_80)); ([81](#_ENREF_81)); ([82](#_ENREF_82)); ([83](#_ENREF_83)); ([84](#_ENREF_84)); ([85](#_ENREF_85)); ([86](#_ENREF_86)); ([87](#_ENREF_87)); ([88](#_ENREF_88)); ([89](#_ENREF_89)); ([90](#_ENREF_90)); ([91](#_ENREF_91)); ([92](#_ENREF_92)); ([93](#_ENREF_93)); ([94](#_ENREF_94)); ([95](#_ENREF_95)); ([96](#_ENREF_96)); ([97](#_ENREF_97)); ([98](#_ENREF_98)); ([99](#_ENREF_99)); ([100](#_ENREF_100)); ([101](#_ENREF_101)); ([102](#_ENREF_102)); ([103](#_ENREF_103)); ([104](#_ENREF_104)); ([105](#_ENREF_105)); ([106](#_ENREF_106)); ([107](#_ENREF_107)); ([108](#_ENREF_108)); ([109](#_ENREF_109)); ([110](#_ENREF_110)); ([111](#_ENREF_111)); ([112](#_ENREF_112)); ([113](#_ENREF_113)); ([114](#_ENREF_114)); ([115](#_ENREF_115)); ([116](#_ENREF_116)); ([117](#_ENREF_117)); ([118](#_ENREF_118)); ([119](#_ENREF_119)); ([120](#_ENREF_120)); ([121](#_ENREF_121)); ([122](#_ENREF_122)); ([123](#_ENREF_123)); ([124](#_ENREF_124)); ([125](#_ENREF_125)); ([126](#_ENREF_126)); ([127](#_ENREF_127)); ([128](#_ENREF_128)); ([129](#_ENREF_129)); ([130](#_ENREF_130)); ([131](#_ENREF_131)); ([132](#_ENREF_132)); ([133](#_ENREF_133)) | |  |
|  | **M269V / V269M** | | ([134](#_ENREF_134)); ([72](#_ENREF_72));  ([125](#_ENREF_125)); ([135](#_ENREF_135));  ([50](#_ENREF_50));  ([55](#_ENREF_55))  ([84](#_ENREF_84));  ([136](#_ENREF_136));  ([123](#_ENREF_123));  ([125](#_ENREF_125)); ([137](#_ENREF_137)); ([62](#_ENREF_62));  ([29](#_ENREF_29));  ([100](#_ENREF_100));  ([106](#_ENREF_106)); ([138](#_ENREF_138)) |  | **S118G** | | ([139](#_ENREF_139)) | |  | | **E3 protein 113 position 604** | | ([140](#_ENREF_140)) |  | **Capsid P23S** | | ([24](#_ENREF_24)); ([72](#_ENREF_72)); ([135](#_ENREF_135)); ([88](#_ENREF_88)); ([84](#_ENREF_84)); ([106](#_ENREF_106)) |  | **L20M (unique to American sequences in database)** | ([115](#_ENREF_115)); ([141](#_ENREF_141)) | |  |
|  |  | |  |  | **K252Q** | | ([25](#_ENREF_25));  ([29](#_ENREF_29));  ([62](#_ENREF_62));  ([86](#_ENREF_86));  ([88](#_ENREF_88));  ([90](#_ENREF_90));  ([129](#_ENREF_129));  ([72](#_ENREF_72));  ([80](#_ENREF_80)) | |  | | **E3 protein 279 position 638** | | ([140](#_ENREF_140)) |  | **Capsid S23P** | | ([25](#_ENREF_25)) |  | **T58A** | ([142](#_ENREF_142)) | |  |
|  | **D284E** | | ([134](#_ENREF_134)); ([72](#_ENREF_72)); ([125](#_ENREF_125)); ([135](#_ENREF_135)); ([50](#_ENREF_50)); ([55](#_ENREF_55)); ([88](#_ENREF_88)); ([25](#_ENREF_25)); ([100](#_ENREF_100)); ([106](#_ENREF_106)); ([107](#_ENREF_107)); ([136](#_ENREF_136)); ([123](#_ENREF_123)); ([125](#_ENREF_125)); ([137](#_ENREF_137)); ([138](#_ENREF_138)) |  | **L210Q** | | ([55](#_ENREF_55)); ([29](#_ENREF_29)); ([143](#_ENREF_143)); ([60](#_ENREF_60)); ([73](#_ENREF_73)); ([144](#_ENREF_144)) | |  | | **E3 protein 313 position 693** | | ([140](#_ENREF_140)) |  | **Capsid V27I** | | ([24](#_ENREF_24)); ([72](#_ENREF_72)); ([135](#_ENREF_135)); ([88](#_ENREF_88)); ([84](#_ENREF_84)); ([106](#_ENREF_106)) |  | **V8I** | ([135](#_ENREF_135)); ([88](#_ENREF_88)); ([72](#_ENREF_72)) | |  |
|  | **D284Q** | | ([134](#_ENREF_134)); ([50](#_ENREF_50)) |  | **R198Q** | | ([29](#_ENREF_29)) | |  | | **E3 protein 368 position 731** | | ([140](#_ENREF_140)) |  | **Capsid C-S23 (unique in all India 06 and Italy 07 isolates)** | | ([142](#_ENREF_142)) |  | **8I** | ([145](#_ENREF_145)) | |  |
|  | **V1022I** | | ([106](#_ENREF_106)) |  | **G82R** | | ([118](#_ENREF_118)); ([129](#_ENREF_129)) | |  | | **E3 protein 406 position 738** | | ([140](#_ENREF_140)) |  | **Capsid A264V** | | ([39](#_ENREF_39)) |  | **18V** | ([25](#_ENREF_25)) | |  |
|  | **284E** | | ([145](#_ENREF_145)) |  | **G55R** | | ([139](#_ENREF_139)); ([142](#_ENREF_142)); ([146](#_ENREF_146)) | |  | |  | |  |  | **Capsid K63R** | | ([72](#_ENREF_72)); ([106](#_ENREF_106)) |  | **L39V** | ([25](#_ENREF_25)) | |  |
|  | **V14A** | | ([102](#_ENREF_102)) |  | **M311T** | | ([25](#_ENREF_25)) | |  | |  | |  |  | **Capsid N80D** | | ([25](#_ENREF_25)); ([106](#_ENREF_106)) |  | **154V** | ([72](#_ENREF_72)) | |  |
|  | **D1093E** | | ([106](#_ENREF_106)) |  | **V318M** | | ([25](#_ENREF_25)) | |  | | **S18F** | | ([29](#_ENREF_29)); ([84](#_ENREF_84)) |  | **Capsid T24I** | | ([140](#_ENREF_140)) |  | **V756I** | ([106](#_ENREF_106)) | |  |
|  | **K211E / E211K** | | ([134](#_ENREF_134)); ([147](#_ENREF_147)); ([61](#_ENREF_61)); ([148](#_ENREF_148)); ([115](#_ENREF_115)); ([116](#_ENREF_116)); ([136](#_ENREF_136)); ([129](#_ENREF_129)); ([137](#_ENREF_137)); ([149](#_ENREF_149)); ([63](#_ENREF_63)); ([145](#_ENREF_145)); ([135](#_ENREF_135)); ([123](#_ENREF_123)) |  | **T375S** | | ([25](#_ENREF_25)) | |  | | **A53V** | | ([142](#_ENREF_142)) |  | **Capsid 128T (unique)** | | ([140](#_ENREF_140)); ([88](#_ENREF_88)) |  | **A47S** | ([115](#_ENREF_115)) | |  |
|  | **211E** | | ([63](#_ENREF_63)); ([145](#_ENREF_145)) |  | **1377V** | | ([25](#_ENREF_25)) | |  | | **V421** | | ([25](#_ENREF_25)) |  | **Capsid R78Q** | | ([140](#_ENREF_140)); ([135](#_ENREF_135)) |  | **F48L** | ([115](#_ENREF_115)) | |  |
|  | **K211N** | | ([62](#_ENREF_62)); ([25](#_ENREF_25)); ([29](#_ENREF_29)); ([84](#_ENREF_84)); ([91](#_ENREF_91)) |  | **386V** | | ([25](#_ENREF_25)) | |  | | **P59S** | | ([25](#_ENREF_25)) |  | **Capsid R54C** | | ([97](#_ENREF_97)) |  | **M20L** | ([140](#_ENREF_140)) | |  |
|  | **V291I (non-synonymous mutation)** | | ([73](#_ENREF_73)); ([128](#_ENREF_128)); ([129](#_ENREF_129)) |  | **337I** | | ([118](#_ENREF_118)) | |  | | **V303I** | | ([25](#_ENREF_25)) |  | **Capsid D132N** | | ([97](#_ENREF_97)) |  | **M20 (unique signature in western hemisphere isolates)** | ([140](#_ENREF_140)) | |  |
|  | **V4A** | | ([25](#_ENREF_25)); ([90](#_ENREF_90)); ([88](#_ENREF_88)) |  | **Y69H** | | ([25](#_ENREF_25)) | |  | | **P320S** | | ([25](#_ENREF_25)) |  | **Capsid K37Q** | | ([135](#_ENREF_135)) |  | **M45T** | ([135](#_ENREF_135)) | |  |
|  | **V18I** | | ([25](#_ENREF_25)) |  | **V162A** | | ([25](#_ENREF_25)) | |  | | **K30R (unique)** | | ([72](#_ENREF_72)) |  | **Capsid V48A** | | ([135](#_ENREF_135)) |  | **T47A** | ([135](#_ENREF_135)) | |  |
|  | **H107Q** | | ([25](#_ENREF_25)) |  | **H142Y** | | ([25](#_ENREF_25)) | |  | | **I24T** | | ([72](#_ENREF_72)) |  | **Capsid K73R** | | ([135](#_ENREF_135)) |  | **L52M** | ([135](#_ENREF_135)) | |  |
|  | **R246C** | | ([25](#_ENREF_25)) |  | **G60D** | | ([25](#_ENREF_25)); ([74](#_ENREF_74)) | |  | | **I284T** | | ([106](#_ENREF_106)) |  | **Capsid M81T** | | ([135](#_ENREF_135)) |  | **S60G (unique)** | ([88](#_ENREF_88)) | |  |
|  | **V213I** | | ([25](#_ENREF_25)) |  | **I211T** | | ([25](#_ENREF_25)); ([72](#_ENREF_72)); ([55](#_ENREF_55)); ([35](#_ENREF_35)); ([143](#_ENREF_143)); ([74](#_ENREF_74)) | |  | | **R19Q** | | ([140](#_ENREF_140)) |  | **Capsid V93A** | | ([135](#_ENREF_135)) |  | **6K protein 20 position 864** | ([140](#_ENREF_140)) | |  |
|  | **A145V (non synonymous mutation)** | | ([128](#_ENREF_128)) |  | **211T** | | ([45](#_ENREF_45)); ([114](#_ENREF_114)) | |  | | **A75V** | | ([140](#_ENREF_140)) |  | **Capsid I27V** | | ([62](#_ENREF_62)) |  |  |  | |  |
|  | **I162V (non synonymous mutation)** | | ([128](#_ENREF_128)) |  | **V2221** | | ([29](#_ENREF_29)); ([84](#_ENREF_84)) | |  | | **I101V** | | ([140](#_ENREF_140)) |  | **Capsid G199D (S3 Peptidase unique)** | | ([88](#_ENREF_88)) |  |  |  | |  |
|  | **98A** | | ([20](#_ENREF_20)) |  | **V264A** | | ([146](#_ENREF_146)); ([147](#_ENREF_147)); ([116](#_ENREF_116)); ([129](#_ENREF_129)) | |  | | **Q19R** | | ([150](#_ENREF_150)) |  | **Capsid 127V** | | ([62](#_ENREF_62)) |  |  |  | |  |
|  | **98T (found in endemic Asian strains)** | | ([20](#_ENREF_20)); ([45](#_ENREF_45)); ([128](#_ENREF_128)) |  | **S194G** | | ([72](#_ENREF_72)); ([151](#_ENREF_151)) | |  | | **K33E** | | ([135](#_ENREF_135)) |  | **Capsid (position 27)** | | ([62](#_ENREF_62)) |  |  |  | |  |
|  | **A98T** | | ([63](#_ENREF_63)); ([128](#_ENREF_128)) |  | **V368A (unique to American sequences in dataset)** | | ([115](#_ENREF_115)); ([141](#_ENREF_141));  ([115](#_ENREF_115)) | |  | | **S44R** | | ([135](#_ENREF_135)) |  | **Capsid protein 24 position 28** | | ([140](#_ENREF_140)) |  |  |  | |  |
|  | **S77N** | | ([139](#_ENREF_139)) |  | **D117G** | | ([139](#_ENREF_139)) | |  | | **R60H** | | ([135](#_ENREF_135)) |  | **Capsid protein 28 position 78** | | ([140](#_ENREF_140)) |  |  |  | |  |
|  | **V163A** | | ([139](#_ENREF_139)) |  | **S1G** | | ([139](#_ENREF_139)) | |  | | **R62Q** | | ([135](#_ENREF_135)) |  | **Capsid protein 78 position 280** | | ([140](#_ENREF_140)) |  |  |  | |  |
|  | **1278V** | | ([139](#_ENREF_139)) |  | **V113A** | | ([139](#_ENREF_139)) | |  | | **S15F** | | ([62](#_ENREF_62)) |  |  | |  |  |  |  | |  |
|  | **V367A** | | ([139](#_ENREF_139)) |  | **S118I** | | ([139](#_ENREF_139)) | |  | | **R44G (unique)** | | ([88](#_ENREF_88)) |  |  | |  |  |  |  | |  |
|  | **L211G** | | ([134](#_ENREF_134)) |  | **D59N** | | ([152](#_ENREF_152)) | |  | | **Q52R** | | ([153](#_ENREF_153)) |  |  | |  |  |  |  | |  |
|  | **V322A** | | ([134](#_ENREF_134)); ([154](#_ENREF_154)); ([125](#_ENREF_125)); ([155](#_ENREF_155)); ([50](#_ENREF_50)); ([72](#_ENREF_72)); ([125](#_ENREF_125)) |  | **E79K** | | ([146](#_ENREF_146)) | |  | | **Position 15** | | ([62](#_ENREF_62)) |  |  | |  |  |  |  | |  |
|  | **E209V** | | ([31](#_ENREF_31)) |  | **S159R** | | ([146](#_ENREF_146)) | |  | | **E3 protein 19 position 336** | | ([140](#_ENREF_140)) |  |  | |  |  |  |  | |  |
|  | **A169V** | | ([138](#_ENREF_138)) |  | **E168K** | | ([146](#_ENREF_146)) | |  | | **E3 protein 75 position 362** | | ([140](#_ENREF_140)) |  |  | |  |  |  |  | |  |
|  | **K61T** | | ([152](#_ENREF_152)) |  | **D166E** | | ([146](#_ENREF_146)) | |  | | **E3 protein 101 position 438** | | ([140](#_ENREF_140)) |  |  | |  |  |  |  | |  |
|  | **V80I:A129V** | | ([32](#_ENREF_32)) |  | **H99Y** | | ([146](#_ENREF_146)) | |  | |  | |  |  |  | |  |  |  |  | |  |
|  | **P304L (unique)** | | ([88](#_ENREF_88)); ([35](#_ENREF_35)) |  | **H131Y** | | ([35](#_ENREF_35)) | |  | |  | |  |  |  | |  |  |  |  | |  |
|  | **S250P** | | ([39](#_ENREF_39)) |  | **E247A** | | ([35](#_ENREF_35)) | |  | |  | |  |  |  | |  |  |  |  | |  |
|  | **K132Q** | | ([59](#_ENREF_59)); ([137](#_ENREF_137)) |  | **R82G** | | ([143](#_ENREF_143)) | |  | |  | |  |  |  | |  |  |  |  | |  |
|  | **S168L** | | ([156](#_ENREF_156)) |  | **V229I** | | ([143](#_ENREF_143)) | |  | |  | |  |  |  | |  |  |  |  | |  |
|  | **D183V** | | ([156](#_ENREF_156)) |  | **S375T** | | ([72](#_ENREF_72)); ([88](#_ENREF_88)) | |  | |  | |  |  |  | |  |  |  |  | |  |
|  | **A377T** | | ([25](#_ENREF_25)) |  | **H313Y** | | ([39](#_ENREF_39)) | |  | |  | |  |  |  | |  |  |  |  | |  |
|  | **C555T** | | ([156](#_ENREF_156)) |  | **312M** | | ([145](#_ENREF_145)) | |  | |  | |  |  |  | |  |  |  |  | |  |
|  | **T552C** | | ([72](#_ENREF_72)) |  | **386A** | | ([145](#_ENREF_145)) | |  | |  | |  |  |  | |  |  |  |  | |  |
|  | **N349D** | | ([90](#_ENREF_90)) |  | **F84L (unique)** | | ([72](#_ENREF_72)) | |  | |  | |  |  |  | |  |  |  |  | |  |
| C300T (synonymous mutation) |  | | ([91](#_ENREF_91)) |  | **Q307R (unique)** | | ([72](#_ENREF_72)) | |  | |  | |  |  |  | |  |  |  |  | |  |
| A363G (synonymous mutation) |  | | ([91](#_ENREF_91)) |  | **G57K** | | ([72](#_ENREF_72)) | |  | |  | |  |  |  | |  |  |  |  | |  |
| A105G (synonymous mutation) |  | | ([91](#_ENREF_91)) |  | **I74M** | | ([72](#_ENREF_72)) | |  | |  | |  |  |  | |  |  |  |  | |  |
| C1308T (synonymous mutation) |  | | ([91](#_ENREF_91)) |  | **G79E** | | ([72](#_ENREF_72)) | |  | |  | |  |  |  | |  |  |  |  | |  |
|  | **V14A** | | ([102](#_ENREF_102)) |  | **N160T** | | ([72](#_ENREF_72)) | |  | |  | |  |  |  | |  |  |  |  | |  |
|  | **M1078V** | | ([106](#_ENREF_106)) |  | **A164T** | | ([72](#_ENREF_72)) | |  | |  | |  |  |  | |  |  |  |  | |  |
|  | **K1020N** | | ([106](#_ENREF_106)); ([157](#_ENREF_157)); ([106](#_ENREF_106)) |  | **L181M** | | ([72](#_ENREF_72)) | |  | |  | |  |  |  | |  |  |  |  | |  |
|  | **M1078V** | | ([106](#_ENREF_106)) |  | **K52Q** | | ([72](#_ENREF_72)) | |  | |  | |  |  |  | |  |  |  |  | |  |
|  | **D1086V** | | ([157](#_ENREF_157)) |  | **M267R** | | ([72](#_ENREF_72)) | |  | |  | |  |  |  | |  |  |  |  | |  |
|  | **L133F** | | ([115](#_ENREF_115)) |  | **S299M** | | ([72](#_ENREF_72)) | |  | |  | |  |  |  | |  |  |  |  | |  |
|  | **V813A** | | ([122](#_ENREF_122)) |  | **T312M** | | ([72](#_ENREF_72)) | |  | |  | |  |  |  | |  |  |  |  | |  |
|  | **E839V** | | ([122](#_ENREF_122)) |  | **A344T** | | ([72](#_ENREF_72)) | |  | |  | |  |  |  | |  |  |  |  | |  |
|  | **N949I** | | ([122](#_ENREF_122)) |  | **V386A** | | ([72](#_ENREF_72)); ([88](#_ENREF_88)) | |  | |  | |  |  |  | |  |  |  |  | |  |
|  | **S977L** | | ([122](#_ENREF_122)) |  | **S375T** | | ([143](#_ENREF_143)); ([72](#_ENREF_72)) | |  | |  | |  |  |  | |  |  |  |  | |  |
|  | **D992V** | | ([122](#_ENREF_122)) | **A9114G** |  | | ([73](#_ENREF_73)) | |  | |  | |  |  |  | |  |  |  |  | |  |
|  | **K1020N** | | ([122](#_ENREF_122)) |  | **R178H** | | ([84](#_ENREF_84)) | |  | |  | |  |  |  | |  |  |  |  | |  |
|  | **D1021G** | | ([122](#_ENREF_122)) |  | **I536T** | | ([106](#_ENREF_106)) | |  | |  | |  |  |  | |  |  |  |  | |  |
|  | **F1049S/L** | | ([122](#_ENREF_122)) |  | **T637M** | | ([106](#_ENREF_106)) | |  | |  | |  |  |  | |  |  |  |  | |  |
|  | **G1057V** | | ([122](#_ENREF_122)) |  | **S700T** | | ([106](#_ENREF_106)) | |  | |  | |  |  |  | |  |  |  |  | |  |
|  | **N1079D** | | ([122](#_ENREF_122)) |  | **V711A** | | ([106](#_ENREF_106)) | |  | |  | |  |  |  | |  |  |  |  | |  |
|  | **D1101G** | | ([122](#_ENREF_122)) |  | **T411I** | | ([155](#_ENREF_155)) | |  | |  | |  |  |  | |  |  |  |  | |  |
|  | **T1106A** | | ([122](#_ENREF_122)) |  | **A489T** | | ([106](#_ENREF_106)) | |  | |  | |  |  |  | |  |  |  |  | |  |
|  | **A1138V** | | ([122](#_ENREF_122)) |  | **N7S / S7N** | | ([108](#_ENREF_108)) | |  | |  | |  |  |  | |  |  |  |  | |  |
|  | **M1142T** | | ([122](#_ENREF_122)) |  | **N60D /D60N** | | ([108](#_ENREF_108)); ([45](#_ENREF_45)) | |  | |  | |  |  |  | |  |  |  |  | |  |
|  | **E1152G** | | ([122](#_ENREF_122)) |  | **W64R** | | ([108](#_ENREF_108)) | |  | |  | |  |  |  | |  |  |  |  | |  |
|  | **T1167M** | | ([122](#_ENREF_122)) |  | **E208K /208R** | | ([108](#_ENREF_108)) | |  | |  | |  |  |  | |  |  |  |  | |  |
|  | **S1180F** | | ([122](#_ENREF_122)) |  | **I2T** | | ([151](#_ENREF_151)) | |  | |  | |  |  |  | |  |  |  |  | |  |
|  | **4VA** | | ([122](#_ENREF_122)); ([129](#_ENREF_129)) |  | **H5N** | | ([151](#_ENREF_151)) | |  | |  | |  |  |  | |  |  |  |  | |  |
|  | **G248V (unique)** | | ([88](#_ENREF_88)); ([122](#_ENREF_122)) |  | **G118S** | | ([151](#_ENREF_151)) | |  | |  | |  |  |  | |  |  |  |  | |  |
|  | **P294L** | | ([135](#_ENREF_135)); ([50](#_ENREF_50)); ([123](#_ENREF_123)) |  | **L248F** | | ([115](#_ENREF_115)) | |  | |  | |  |  |  | |  |  |  |  | |  |
|  | **S295F** | | ([135](#_ENREF_135)); ([50](#_ENREF_50)); ([123](#_ENREF_123)) |  | **H351R** | | ([115](#_ENREF_115)) | |  | |  | |  |  |  | |  |  |  |  | |  |
|  | **A66V** | | ([129](#_ENREF_129)) |  | **V371L** | | ([115](#_ENREF_115)) | |  | |  | |  |  |  | |  |  |  |  | |  |
|  | **L33 (silent mutation)** | | ([128](#_ENREF_128)) |  | **337V** | | ([118](#_ENREF_118)) | |  | |  | |  |  |  | |  |  |  |  | |  |
|  | **T228 (silent mutation)** | | ([128](#_ENREF_128)) |  | **1217V** | | ([118](#_ENREF_118)) | |  | |  | |  |  |  | |  |  |  |  | |  |
|  | **F365 (silent mutation)** | | ([128](#_ENREF_128)) |  | **G249R** | | ([118](#_ENREF_118)) | |  | |  | |  |  |  | |  |  |  |  | |  |
|  | **P383 (silent mutation)** | | ([128](#_ENREF_128)) |  | **K546R** | | ([119](#_ENREF_119)) | |  | |  | |  |  |  | |  |  |  |  | |  |
|  | **S72N** | | ([135](#_ENREF_135)) |  | **V113A** | | ([140](#_ENREF_140)) | |  | |  | |  |  |  | |  |  |  |  | |  |
|  | **T98A** | | ([135](#_ENREF_135)) |  | **G279E** | | ([140](#_ENREF_140)) | |  | |  | |  |  |  | |  |  |  |  | |  |
|  | **A145T** | | ([135](#_ENREF_135)) |  | **H313L** | | ([140](#_ENREF_140)) | |  | |  | |  |  |  | |  |  |  |  | |  |
|  | **S225A** | | ([135](#_ENREF_135)) |  | **A368V** | | ([140](#_ENREF_140)) | |  | |  | |  |  |  | |  |  |  |  | |  |
|  | **S304P** | | ([135](#_ENREF_135)) |  | **A406T** | | ([140](#_ENREF_140)) | |  | |  | |  |  |  | |  |  |  |  | |  |
|  | **P397L** | | ([135](#_ENREF_135)); ([150](#_ENREF_150)) |  | **A368 (unique signature in western hemisphere isolates)** | | ([140](#_ENREF_140)) | |  | |  | |  |  |  | |  |  |  |  | |  |
|  | **A316V** | | ([50](#_ENREF_50)) |  | **N72S** | | ([129](#_ENREF_129)) | |  | |  | |  |  |  | |  |  |  |  | |  |
|  | **C328W** | | ([50](#_ENREF_50)) |  | **L357Q** | | ([129](#_ENREF_129)) | |  | |  | |  |  |  | |  |  |  |  | |  |
|  | **V04A (unique)** | | ([88](#_ENREF_88)) |  | **I377T** | | ([129](#_ENREF_129)) | |  | |  | |  |  |  | |  |  |  |  | |  |
|  | **E127G (unique)** | | ([88](#_ENREF_88)) |  | **V251A** | | ([158](#_ENREF_158)) | |  | |  | |  |  |  | |  |  |  |  | |  |
|  | **D184G (unique)** | | ([88](#_ENREF_88)) |  | **H313Y** | | ([39](#_ENREF_39)) | |  | |  | |  |  |  | |  |  |  |  | |  |
|  | **Y195H (unique)** | | ([88](#_ENREF_88)) |  | **252Q** | | ([62](#_ENREF_62)) | |  | |  | |  |  |  | |  |  |  |  | |  |
|  | **M197V (unique)** | | ([88](#_ENREF_88)) |  | **N72S (unique)** | | ([88](#_ENREF_88)) | |  | |  | |  |  |  | |  |  |  |  | |  |
|  | **G248E (unique)** | | ([88](#_ENREF_88)) |  | **V229I (unique)** | | ([88](#_ENREF_88)) | |  | |  | |  |  |  | |  |  |  |  | |  |
|  | **T396A (unique)** | | ([88](#_ENREF_88)) |  | **K252H** | | ([129](#_ENREF_129)) | |  | |  | |  |  |  | |  |  |  |  | |  |
|  | **K411R (unique)** | | ([88](#_ENREF_88)) |  | **E361G (unique)** | | ([88](#_ENREF_88)) | |  | |  | |  |  |  | |  |  |  |  | |  |
|  | **C433R (unique)** | | ([88](#_ENREF_88)) |  | **C416G (unique)** | | ([88](#_ENREF_88)) | |  | |  | |  |  |  | |  |  |  |  | |  |
|  | **F118L** | | ([153](#_ENREF_153)) | **syn>I (at nt position 8892)** |  | | ([153](#_ENREF_153)) | |  | |  | |  |  |  | |  |  |  |  | |  |
| syn>S (at nt position 11046) |  | | ([153](#_ENREF_153)) | **syn>G (at nt position 9690)** |  | | ([153](#_ENREF_153)) | |  | |  | |  |  |  | |  |  |  |  | |  |
| syn>T (at nt position 10104) |  | | ([153](#_ENREF_153)) | **syn>F at nt position 8874** |  | | ([153](#_ENREF_153)) | |  | |  | |  |  |  | |  |  |  |  | |  |
|  | **G274V ( 95% of polulation in mammalian cells)** | | ([153](#_ENREF_153)) |  | **V251A** | | ([158](#_ENREF_158)) | |  | |  | |  |  |  | |  |  |  |  | |  |
|  | **G832R** | | ([155](#_ENREF_155)) |  | **T419I** | | ([155](#_ENREF_155)) | |  | |  | |  |  |  | |  |  |  |  | |  |
|  | **V7M (non- synonymous mutation)** | | ([159](#_ENREF_159)) | **A9114G** |  | | ([73](#_ENREF_73)) | |  | |  | |  |  |  | |  |  |  |  | |  |
|  | **E1 protein 55** | | ([140](#_ENREF_140)) |  |  | |  | |  | |  | |  |  |  | |  |  |  |  | |  |
| Two synonymous mutations at nucleotide positions 300 (C300T); and 363 A363G of the E1 gene. 2 synonymous mutations at nucleotide positions 105 (A105G); 1308 (C1308T) |  | | ([91](#_ENREF_91)) |  |  | |  | |  | |  | |  |  |  | |  |  |  |  | |  |
| Two mutations viz. T/C at (1059); 3 and A/G at 10624 were found in the 933 bp of the E1 gene |  | | ([148](#_ENREF_148)) |  |  | |  | |  | |  | |  |  |  | |  |  |  |  | |  |
| A306G (synonymous mutation) |  | | ([156](#_ENREF_156)) |  |  | |  | |  | |  | |  |  |  | |  |  |  |  | |  |
| C384T (synonymous mutation) |  | | ([156](#_ENREF_156)) |  |  | |  | |  | |  | |  |  |  | |  |  |  |  | |  |
| T519C (synonymous mutation) |  | | ([156](#_ENREF_156)) |  |  | |  | |  | |  | |  |  |  | |  |  |  |  | |  |
| C531T (synonymous mutation) |  | | ([156](#_ENREF_156)) |  |  | |  | |  | |  | |  |  |  | |  |  |  |  | |  |
| T552C (synonymous mutation) |  | | ([156](#_ENREF_156)) |  |  | |  | |  | |  | |  |  |  | |  |  |  |  | |  |
| C555T (synonymous mutation) |  | | ([156](#_ENREF_156)) |  |  | |  | |  | |  | |  |  |  | |  |  |  |  | |  |
|  |  | |  |  |  | |  | |  | |  | |  |  |  | |  |  |  |  | |  |
| *Non-structural proteins* | | | | | | | | | | | | | | | | | | |  |  | |  |
|  |  | |  |  |  | |  | | |  |  | |  |  |  | |  |  |  |  | |  |
| nsP1 | | | | **nsP2** | | | | | | **nsP3** | | | | **nsP4** | | | |  |  |  | |  |
| *Nucleotide* | | *Amino acid* | *Refid* | *Nucleotide* | | *Amino acid* | | *Refid* | | *Nucleotide* | | *Amino acid* | *Refid* | *Nucleotide* | | *Amino acid* | *Refid* |  |  |  | |  |
|  | | **R171Q** | ([142](#_ENREF_142)); ([129](#_ENREF_129)) |  | | **L539S** | | ([80](#_ENREF_80)); ([135](#_ENREF_135)); ([62](#_ENREF_62)); ([88](#_ENREF_88)); ([29](#_ENREF_29)); ([84](#_ENREF_84)); ([129](#_ENREF_129)); ([73](#_ENREF_73)) | |  | | **Y38H** | ([29](#_ENREF_29)); ([84](#_ENREF_84)); ([129](#_ENREF_129)); ([62](#_ENREF_62)) |  | | **R82S** | ([72](#_ENREF_72)); ([80](#_ENREF_80)); ([84](#_ENREF_84)) |  |  |  | |  |
|  | | **A101V** | ([106](#_ENREF_106)); ([138](#_ENREF_138)) |  | | **M703L** | | ([142](#_ENREF_142)) | |  | | **I394M** | ([62](#_ENREF_62)) |  | | **Y87H** | ([72](#_ENREF_72)) |  |  |  | |  |
|  | | **W456R** | ([142](#_ENREF_142)) |  | | **T599I** | | ([39](#_ENREF_39)) | |  | | **T444M** | ([29](#_ENREF_29)); ([84](#_ENREF_84)); ([62](#_ENREF_62)) |  | | **N595K** | ([72](#_ENREF_72)) |  |  |  | |  |
|  | | **R501L** | ([142](#_ENREF_142)) |  | | **P79S** | | ([72](#_ENREF_72)) | |  | | **T4365I** | ([160](#_ENREF_160)) |  | | **R600I** | ([72](#_ENREF_72)) |  |  |  | |  |
|  | | **M376 (unique in all India 06 and Italy o7 isolates)** | ([142](#_ENREF_142)) |  | | **T1210M** | | ([106](#_ENREF_106)) | |  | | **G117R** | ([108](#_ENREF_108)); ([118](#_ENREF_118)); ([135](#_ENREF_135)) |  | | **L606M** | ([161](#_ENREF_161)) |  |  |  | |  |
| R (A or G); at nucleotide position 764 from start of genome | |  | ([160](#_ENREF_160)) |  | | **A57T / A57V** | | ([118](#_ENREF_118)) | |  | | **S472N** | ([24](#_ENREF_24)) |  | | **P93S** | ([118](#_ENREF_118)) |  |  |  | |  |
|  | | **T351A** | ([39](#_ENREF_39)) |  | | **318T** | | ([118](#_ENREF_118)) | |  | | **silent mutation at position 4540** | ([160](#_ENREF_160)) |  | | **I563T** | ([84](#_ENREF_84)) |  |  |  | |  |
|  | | **P29S** | ([72](#_ENREF_72)) |  | | **K510T** | | ([118](#_ENREF_118)) | |  | | **I5302M** | ([160](#_ENREF_160)) |  | | **164F** | ([118](#_ENREF_118)) |  |  |  | |  |
|  | | **N186D** | ([72](#_ENREF_72)) |  | | **1685D** | | ([118](#_ENREF_118)) | |  | | **E460 deletion (unique to CSF isolate)** | ([107](#_ENREF_107)) |  | | **353F** | ([118](#_ENREF_118)) |  |  |  | |  |
|  | | **Q488** | ([84](#_ENREF_84)) |  | | **V153A** | | ([140](#_ENREF_140)) | | **nsP3 -a C–T change at nucleotide position 4365 from start of genome** | |  | ([160](#_ENREF_160)) |  | | **C483Y** | ([118](#_ENREF_118)) |  |  |  | |  |
|  | | **T60A** | ([84](#_ENREF_84)) |  | | **L670M** | | ([140](#_ENREF_140)) | | **nsP3 - a T–C change at nucleotide position 4540 from start of genome** | |  | ([160](#_ENREF_160)) |  | | **R99Q** | ([140](#_ENREF_140)) |  |  |  | |  |
|  | | **M184T** | ([102](#_ENREF_102)) |  | | **N667Y** | | ([158](#_ENREF_158)) | | **nsP3 - R (A or G) at nucleotide position 5302 from start of genome** | |  | ([160](#_ENREF_160)) |  | | **A459V** | ([140](#_ENREF_140)) |  |  |  | |  |
|  | | **G764R** | ([160](#_ENREF_160)) |  | | **Y642N (unique to CSF sample)** | | ([107](#_ENREF_107)) | |  | | **P355L** | ([39](#_ENREF_39)) |  | | **A177S** | ([129](#_ENREF_129)) |  |  |  | |  |
|  | | **T128K** | ([24](#_ENREF_24)); ([106](#_ENREF_106)); ([135](#_ENREF_135)); ([88](#_ENREF_88)); ([24](#_ENREF_24)); ([84](#_ENREF_84)) |  | | **C586S** | | ([97](#_ENREF_97)) | |  | | **S381Y** | ([39](#_ENREF_39)) |  | | **L42A** | ([135](#_ENREF_135)) |  |  |  | |  |
|  | | **T376M** | ([106](#_ENREF_106)); ([135](#_ENREF_135)); ([88](#_ENREF_88)); ([24](#_ENREF_24)); ([84](#_ENREF_84)) |  | | **L16P** | | ([135](#_ENREF_135)) | |  | | **T338M** | ([72](#_ENREF_72)) |  | | **T58M** | ([135](#_ENREF_135)) |  |  |  | |  |
|  | | **G230R** | ([108](#_ENREF_108)) |  | | **S54N** | | ([135](#_ENREF_135)); ([88](#_ENREF_88)) | |  | | **V1664** | ([84](#_ENREF_84)) |  | | **T75A** | ([135](#_ENREF_135)); ([88](#_ENREF_88)) |  |  |  | |  |
|  | | **M314V** | ([108](#_ENREF_108)) |  | | **S218T** | | ([135](#_ENREF_135)) | |  | | **M394I** | ([84](#_ENREF_84)) |  | | **K85R** | ([135](#_ENREF_135)) |  |  |  | |  |
|  | | **162L** | ([118](#_ENREF_118)) |  | | **L273Q** | | ([135](#_ENREF_135)) | |  | | **M59T** | ([129](#_ENREF_129)) |  | | **A90s** | ([135](#_ENREF_135)) |  |  |  | |  |
|  | | **T301I unique to CSF sample in (**[**107**](#_ENREF_107)**)** | ([107](#_ENREF_107)); ([118](#_ENREF_118)) |  | | **M338K** | | ([135](#_ENREF_135)) | |  | | **T1674M** | ([106](#_ENREF_106)) |  | | **V101T** | ([135](#_ENREF_135)) |  |  |  | |  |
|  | | **T314M** | ([118](#_ENREF_118)) |  | | **H374Y** | | ([135](#_ENREF_135)) | |  | | **G117R** | ([108](#_ENREF_108)) |  | | **R235Q** | ([135](#_ENREF_135)) |  |  |  | |  |
|  | | **R1307I** | ([119](#_ENREF_119)) |  | | **V466M** | | ([135](#_ENREF_135)) | |  | | **M424I** | ([108](#_ENREF_108)) |  | | **T254A** | ([135](#_ENREF_135)); ([88](#_ENREF_88)) |  |  |  | |  |
|  | | **M84V** | ([140](#_ENREF_140)) |  | | **V486I** | | ([135](#_ENREF_135)) | |  | | **unique amino acid insertion positions 376 to 451. This insertion consists of a duplicated sequence of part of the N-terminal domain of nsP3** | ([162](#_ENREF_162)) |  | | **R271K** | ([135](#_ENREF_135)) |  |  |  | |  |
|  | | **A101T** | ([140](#_ENREF_140)) |  | | **1756V** | | ([135](#_ENREF_135)) | |  | | **274T** | ([118](#_ENREF_118)) |  | | **D280E** | ([135](#_ENREF_135)) |  |  |  | |  |
|  | | **V147A** | ([140](#_ENREF_140)) |  | | **S768N** | | ([135](#_ENREF_135)) | |  | | **R1806Q** | ([119](#_ENREF_119)) |  | | **A366T** | ([135](#_ENREF_135)) |  |  |  | |  |
|  | | **K224N** | ([140](#_ENREF_140)) |  | | **A793V** | | ([135](#_ENREF_135)); ([88](#_ENREF_88)) | |  | | **S48C** | ([140](#_ENREF_140)) |  | | **Q500L** | ([135](#_ENREF_135)) |  |  |  | |  |
|  | | **K224R** | ([140](#_ENREF_140)) |  | | **V48A (unique)** | | ([88](#_ENREF_88)) | |  | | **T97I** | ([140](#_ENREF_140)) |  | | **A582V** | ([135](#_ENREF_135)) |  |  |  | |  |
|  | | **S329P** | ([140](#_ENREF_140)) |  | | **V181A (helicase)** | | ([88](#_ENREF_88)) | |  | | **I285V** | ([140](#_ENREF_140)) |  | | **S116P (unique)** | ([88](#_ENREF_88)) |  |  |  | |  |
|  | | **P476T** | ([140](#_ENREF_140)) |  | | **L237R (unique)** | | ([88](#_ENREF_88)) | |  | | **(XXXX); 378 (LPTT)** | ([140](#_ENREF_140)) |  | | **N252D (unique -RNA dependent RNA polymerase (RdRP)** | ([88](#_ENREF_88)) |  |  |  | |  |
|  | | **S3P** | ([135](#_ENREF_135)) |  | | **L238P** | | ([88](#_ENREF_88)) | |  | | **S238N** | ([129](#_ENREF_129)) |  | | **V289A (unique)** | ([88](#_ENREF_88)) |  |  |  | |  |
|  | | **S34P** | ([135](#_ENREF_135)) |  | | **K329E (unique)** | | ([88](#_ENREF_88)) | |  | | **N409T** | ([129](#_ENREF_129)) |  | | **M479V (unique)** | ([88](#_ENREF_88)) |  |  |  | |  |
|  | | **V153I** | ([135](#_ENREF_135)) |  | | **N411D (unique)** | | ([88](#_ENREF_88)) | |  | | **D483N** | ([150](#_ENREF_150)); ([135](#_ENREF_135)) |  | | **M489K (unique)** | ([88](#_ENREF_88)) |  |  |  | |  |
|  | | **M253K** | ([135](#_ENREF_135)) |  | | **L708P** | | ([88](#_ENREF_88)) | |  | | **T77S** | ([135](#_ENREF_135)) |  | | **nsP4 S116P** | ([153](#_ENREF_153)) |  |  |  | |  |
|  | | **G454S** | ([135](#_ENREF_135)) | **syn>H (at nt position 2008)** | |  | | ([153](#_ENREF_153)) | |  | | **V175I** | ([135](#_ENREF_135)) |  | | **nsP4 I141T** | ([153](#_ENREF_153)) |  |  |  | |  |
|  | | **R473S** | ([135](#_ENREF_135)) | **syn>S (at nt position 2038)** | |  | | ([153](#_ENREF_153)) | |  | | **I176V** | ([135](#_ENREF_135)) |  | | **nsP4 L345P** | ([153](#_ENREF_153)) |  |  |  | |  |
|  | | **A478T** | ([135](#_ENREF_135)) |  | | **I320F** | | ([153](#_ENREF_153)) | |  | | **V213M** | ([135](#_ENREF_135)) |  | | **nsP4 I372L** | ([153](#_ENREF_153)) |  |  |  | |  |
|  | | **N486D** | ([135](#_ENREF_135)) |  | | **R331C** | | ([153](#_ENREF_153)) | |  | | **N283S** | ([135](#_ENREF_135)) | **nsP4 syn>A (at nt position 6805)** | |  | ([153](#_ENREF_153)) |  |  |  | |  |
|  | | **Q488R** | ([135](#_ENREF_135)); ([88](#_ENREF_88)) | **syn>T (at nt position 2716 - novel synonymous change)** | |  | | ([153](#_ENREF_153)) | |  | | **V303T** | ([135](#_ENREF_135)) |  | | **nsP4 I404T** | ([153](#_ENREF_153)) |  |  |  | |  |
|  | | **Q491R** | ([135](#_ENREF_135)) | **syn>I (at nt position 2788)** | |  | | ([153](#_ENREF_153)) | |  | | **V331A** | ([135](#_ENREF_135)) |  | | **nsP4 L441Stop** | ([153](#_ENREF_153)) |  |  |  | |  |
|  | | **H507R** | ([135](#_ENREF_135)) | **syn>S (at nt position 2962)** | |  | | ([153](#_ENREF_153)) | |  | | **R332Q** | ([135](#_ENREF_135)) |  | | **nsP4 S475A** | ([153](#_ENREF_153)) |  |  |  | |  |
|  | | **G105R (unique)** | ([88](#_ENREF_88)) | **syn>A (at nt position 3196)** | |  | | ([153](#_ENREF_153)) | |  | | **V334A** | ([135](#_ENREF_135)) | **nsP4 syn>A (at nt position 7159)** | |  | ([153](#_ENREF_153)) |  |  |  | |  |
|  | | **W258R (unique)** | ([88](#_ENREF_88)) |  | | **I461S** | | ([153](#_ENREF_153)) | |  | | **M336T** | ([135](#_ENREF_135)) |  | | **nsP4 L455M (95% of population in insect cells)** | ([153](#_ENREF_153)) |  |  |  | |  |
|  | | **M314L** | ([88](#_ENREF_88)); ([97](#_ENREF_97)) |  | | **V613E** | | ([153](#_ENREF_153)) | |  | | **T337I** | ([135](#_ENREF_135)) |  | | **L385H** | ([158](#_ENREF_158)) |  |  |  | |  |
|  | | **D536G (unique)** | ([88](#_ENREF_88)) |  | | **L637H** | | ([153](#_ENREF_153)) | |  | | **A349V** | ([135](#_ENREF_135)) |  | | **nsP4 protein 99 position 2318** | ([140](#_ENREF_140)) |  |  |  | |  |
|  | | **C134S** | ([153](#_ENREF_153)) |  | | **G772D** | | ([153](#_ENREF_153)) | |  | | **T353I** | ([135](#_ENREF_135)) |  | |  |  |  |  |  | |  |
| syn>L (at nt postiton 479) | |  | ([153](#_ENREF_153)) |  | | **G460S** | | ([153](#_ENREF_153)) | |  | | **del376-382THTLPST** | ([135](#_ENREF_135)) |  | |  |  |  |  |  | |  |
| syn>T at nt position 667 | |  | ([153](#_ENREF_153)) |  | | **I447M** | | ([158](#_ENREF_158)) | |  | | **I383T** | ([135](#_ENREF_135)) |  | |  |  |  |  |  | |  |
| syn>Y (at nt position 1525) | |  | ([153](#_ENREF_153)) | **novel synonymous substitution (T3296C)** | |  | | ([73](#_ENREF_73)) | |  | | **I413T** | ([135](#_ENREF_135)) |  | |  |  |  |  |  | |  |
|  | | **N186T** | ([153](#_ENREF_153)) |  | | **N667Y** | | ([158](#_ENREF_158)) | |  | | **Q434L** | ([135](#_ENREF_135)) |  | |  |  |  |  |  | |  |
|  | | **G220R** | ([153](#_ENREF_153)) |  | | **nsP2 protein 57 position 688** | | ([140](#_ENREF_140)) | |  | | **A437V** | ([135](#_ENREF_135)) |  | |  |  |  |  |  | |  |
|  | | **S245A** | ([153](#_ENREF_153)) |  | | **nsP2 protein 153 position 1205** | | ([140](#_ENREF_140)) | |  | | **I449M** | ([135](#_ENREF_135)) |  | |  |  |  |  |  | |  |
|  | | **V263E** | ([153](#_ENREF_153)) |  | | **nsP2 protein 670 position 1381** | | ([140](#_ENREF_140)) | |  | | **R452Q** | ([135](#_ENREF_135)) |  | |  |  |  |  |  | |  |
|  | | **L346F** | ([153](#_ENREF_153)) | **position 2716 A>G** | |  | | ([153](#_ENREF_153)) | |  | | **I457T** | ([135](#_ENREF_135)) |  | |  |  |  |  |  | |  |
|  | | **S484N** | ([153](#_ENREF_153)) |  | |  | |  | |  | | **T458A** | ([135](#_ENREF_135)) |  | |  |  |  |  |  | |  |
|  | | **E499K** | ([153](#_ENREF_153)) |  | |  | |  | |  | | **V459T** | ([135](#_ENREF_135)) |  | |  |  |  |  |  | |  |
|  | | **protein 84 position 101** | ([140](#_ENREF_140)) |  | |  | |  | |  | | **L461P** | ([135](#_ENREF_135)); ([88](#_ENREF_88)) |  | |  |  |  |  |  | |  |
|  | | **protein 101 position 147** | ([140](#_ENREF_140)) |  | |  | |  | |  | | **S462N** | ([135](#_ENREF_135)) |  | |  |  |  |  |  | |  |
|  | | **protein 147 position 224** | ([140](#_ENREF_140)) |  | |  | |  | |  | | **P471S** | ([135](#_ENREF_135)); ([88](#_ENREF_88)) |  | |  |  |  |  |  | |  |
|  | | **protein 224 position 329** | ([140](#_ENREF_140)) |  | |  | |  | |  | | **D484E** | ([135](#_ENREF_135)) |  | |  |  |  |  |  | |  |
|  | | **protein 329 position 476** | ([140](#_ENREF_140)) |  | |  | |  | |  | | **P26S (macrodomain unique)** | ([88](#_ENREF_88)) |  | |  |  |  |  |  | |  |
|  | | **protein 476 position 592** | ([140](#_ENREF_140)); |  | |  | |  | |  | | **K94Q (unique)** | ([88](#_ENREF_88)) |  | |  |  |  |  |  | |  |
|  | | **E499K** | ([153](#_ENREF_153)); |  | |  | |  | |  | | **L201R (unique)** | ([88](#_ENREF_88)) |  | |  |  |  |  |  | |  |
|  | |  |  |  | |  | |  | |  | | **D250E (unique)** | ([88](#_ENREF_88)) |  | |  |  |  |  |  | |  |
|  | |  |  |  | |  | |  | |  | | **I376T** | ([88](#_ENREF_88)) |  | |  |  |  |  |  | |  |
|  | |  |  |  | |  | |  | |  | | **E440G (unique)** | ([88](#_ENREF_88)) |  | |  |  |  |  |  | |  |
|  | |  |  |  | |  | |  | |  | | **S48R** | ([153](#_ENREF_153)) |  | |  |  |  |  |  | |  |
|  | |  |  |  | |  | |  | |  | | **A49V** | ([153](#_ENREF_153)) |  | |  |  |  |  |  | |  |
|  | |  |  |  | |  | |  | |  | | **A88T** | ([153](#_ENREF_153)) |  | |  |  |  |  |  | |  |
|  | |  |  |  | |  | |  | |  | | **G113R** | ([153](#_ENREF_153)) |  | |  |  |  |  |  | |  |
|  | |  |  |  | |  | |  | |  | | **T135M** | ([153](#_ENREF_153)) |  | |  |  |  |  |  | |  |
|  | |  |  |  | |  | |  | | **syn>R (at nt position 4507 - novel synonymous change)** | |  | ([153](#_ENREF_153)) |  | |  |  |  |  |  | |  |
|  | |  |  |  | |  | |  | | **syn>K (at nt position 4513 - novel synonymous change)** | |  | ([153](#_ENREF_153)) |  | |  |  |  |  |  | |  |
|  | |  |  |  | |  | |  | |  | | **R178Q** | ([153](#_ENREF_153)) |  | |  |  |  |  |  | |  |
|  | |  |  |  | |  | |  | |  | | **S255F** | ([153](#_ENREF_153)) |  | |  |  |  |  |  | |  |
|  | |  |  |  | |  | |  | | **syn>L (at nt position 4864)** | |  | ([153](#_ENREF_153)) |  | |  |  |  |  |  | |  |
|  | |  |  |  | |  | |  | |  | | **S340P** | ([153](#_ENREF_153)) |  | |  |  |  |  |  | |  |
|  | |  |  |  | |  | |  | |  | | **D359A** | ([153](#_ENREF_153)) |  | |  |  |  |  |  | |  |
|  | |  |  |  | |  | |  | |  | | **E368D** | ([153](#_ENREF_153)) |  | |  |  |  |  |  | |  |
|  | |  |  |  | |  | |  | | **nsP3 syn>L (at nt postion 5221)** | |  | ([153](#_ENREF_153)) |  | |  |  |  |  |  | |  |
|  | |  |  |  | |  | |  | |  | | **V408E** | ([153](#_ENREF_153)) |  | |  |  |  |  |  | |  |
|  | |  |  |  | |  | |  | |  | | **C410Stop** | ([153](#_ENREF_153)) |  | |  |  |  |  |  | |  |
|  | |  |  |  | |  | |  | |  | | **E415K** | ([153](#_ENREF_153)) |  | |  |  |  |  |  | |  |
|  | |  |  |  | |  | |  | |  | | **1417K** | ([153](#_ENREF_153)) |  | |  |  |  |  |  | |  |
|  | |  |  |  | |  | |  | |  | | **M420K** | ([153](#_ENREF_153)) |  | |  |  |  |  |  | |  |
|  | |  |  |  | |  | |  | |  | | **V435A** | ([153](#_ENREF_153)) |  | |  |  |  |  |  | |  |
|  | |  |  |  | |  | |  | | **nsP3 syn>L (at nt position 5564)** | |  | ([153](#_ENREF_153)) |  | |  |  |  |  |  | |  |
|  | |  |  |  | |  | |  | |  | | **nsP3 - 5 amino acid deletions at positions 368 to 372** | ([158](#_ENREF_158)) |  | |  |  |  |  |  | |  |
|  | |  |  |  | |  | |  | |  | | **7 amino acid deletion at positions 376–382 in Asian strains** | ([135](#_ENREF_135)) |  | |  |  |  |  |  | |  |
|  | |  |  |  | |  | |  | |  | | **nsP3 (positions 38 394 and 444)** | ([62](#_ENREF_62)) |  | |  |  |  |  |  | |  |
|  | |  |  |  | |  | |  | | **unique amino acid insertion in the nsP3 gene was observed in positions 376 to 451** | |  | ([162](#_ENREF_162)) |  | |  |  |  |  |  | |  |
|  | |  |  |  | |  | |  | |  | | **nsP3 protein 48 position 1430** | ([140](#_ENREF_140)) |  | |  |  |  |  |  | |  |
|  | |  |  |  | |  | |  | |  | | **nsP3 protein 97 position 1618** | ([140](#_ENREF_140)) |  | |  |  |  |  |  | |  |
|  | |  |  |  | |  | |  | |  | | **nsP3 protein 285 position 1711** | ([140](#_ENREF_140)) |  | |  |  |  |  |  | |  |
|  | |  |  |  | |  | |  | |  | | **nsP3 protein 378 position 1958** | ([140](#_ENREF_140)) |  | |  |  |  |  |  | |  |
|  | |  |  |  | |  | |  | | **nsP3 position 4507 C>A; position 4513 A>G; and the 3’UTR position 11952 C>T; position 11953 G>A** | |  | ([153](#_ENREF_153)) |  | |  |  |  |  |  | |  |
|  | |  |  |  | |  | |  | |  | |  |  |  | |  |  |  |  |  | |  |
| 3'UTR region (novel 3’UTR structure increases viral replication in mosquito cells) | | | | | | | | | | | | | |  | |  |  |  |  |  | |  |
| C>T at position 11302 |  | |  | ([153](#_ENREF_153)) |  | |  | |  | |  | |  |  | |  |  |  |  |  | |  |
| T>C at position 11311 |  | |  | ([153](#_ENREF_153)) |  | |  | |  | |  | |  |  | |  |  |  |  |  | |  |
| T>A at postion 11416 |  | |  | ([153](#_ENREF_153)) |  | |  | |  | |  | |  |  | |  |  |  |  |  | |  |
| C>T at position 11525 |  | |  | ([153](#_ENREF_153)) |  | |  | |  | |  | |  |  | |  |  |  |  |  | |  |
| C>T at position 11775 |  | |  | ([153](#_ENREF_153)) |  | |  | |  | |  | |  |  | |  |  |  |  |  | |  |
| C>T at positon 11776 |  | |  | ([153](#_ENREF_153)) |  | |  | |  | |  | |  |  | |  |  |  |  |  | |  |
| C>T at postiion 11791 |  | |  | ([153](#_ENREF_153)) |  | |  | |  | |  | |  |  | |  |  |  |  |  | |  |
|  |  | |  |  |  | |  | |  | |  | |  |  | |  |  |  |  |  | |  |
| 5' UTR region | | | | | | | | | | | | | |  | |  |  |  |  |  | |  |
| A>T at position53 |  | |  |  |  | |  | |  | |  | |  |  | |  |  |  |  |  | |  |
|  |  | |  |  |  | |  | |  | |  | |  |  | |  |  |  |  |  | |  |
| Genomic region unspecified | | | | | | | | | | | | | |  | |  |  |  |  |  | |  |
| *Nucleotide* | *Amino acid* | | *Refid* |  |  | |  | |  | |  | |  |  | |  |  |  |  |  | |  |
| T1381G and five unique nucleotide variations - T3297C; T3397C; C5014T; A6076G; C7450T. |  | | ([88](#_ENREF_88)) |  |  | |  | |  | |  | |  |  | |  |  |  |  |  | |  |
| A1028→V and D1086→E specific to Reunion and Mayotte strains |  | | ([157](#_ENREF_157)) |  |  | |  | |  | |  | |  |  | |  |  |  |  |  | |  |
|  | **nonsynonymous: P156S in the methyl-transferase domain** | | ([119](#_ENREF_119)) |  |  | |  | |  | |  | |  |  | |  |  |  |  |  | |  |
|  | **L233Q** | | ([155](#_ENREF_155)) |  |  | |  | |  | |  | |  |  | |  |  |  |  |  | |  |
|  | **A478V** | | ([155](#_ENREF_155)) |  |  | |  | |  | |  | |  |  | |  |  |  |  |  | |  |
|  | **R569C** | | ([155](#_ENREF_155)) |  |  | |  | |  | |  | |  |  | |  |  |  |  |  | |  |
|  | **V1163I** | | ([155](#_ENREF_155)) |  |  | |  | |  | |  | |  |  | |  |  |  |  |  | |  |

**References:**

1. Delisle E, Rousseau C, Broche B, Leparc-Goffart I, L’Ambert G, Cochet A, Prat C, Foulongne V, Ferre JB, Catelinois O, Flusin O, Tchernonog E, Moussion IE, Wiegandt

A, Septfons A, Mendy A, Moyano MB, Laporte L, Maurel J, Jourdain F, Reynes J, Paty MC, Golliot F.Chikungunya outbreak in Montpellier, France, September to October 2014. Euro Surveill.2015;20(17).

2. Wintachai P, Thuaud F, Basmadjian C, Roytrakul S, Ubol S, Desaubry L, et al. Assessment of flavaglines as potential chikungunya virus entry inhibitors. Microbiology and immunology. 2015;59(3):129-41.

3. Robinson M, Conan A, Duong V, Ly S, Ngan C, Buchy P, et al. A model for a chikungunya outbreak in a rural Cambodian setting: implications for disease control in uninfected areas. PLoS neglected tropical diseases. 2014;8(9):e3120.

4. Kumar A, Mamidi P, Das I, Nayak TK, Kumar S, Chhatai J, et al. A novel 2006 Indian outbreak strain of Chikungunya virus exhibits different pattern of infection as compared to prototype strain. PloS one. 2014;9(1):e85714.

5. Kosasih H, de Mast Q, Widjaja S, Sudjana P, Antonjaya U, Ma'roef C, et al. Evidence for endemic chikungunya virus infections in Bandung, Indonesia. PLoS neglected tropical diseases. 2013;7(10):e2483.

6. Horwood P, Bande G, Dagina R, Guillaumot L, Aaskov J, Pavlin B. The threat of chikungunya in Oceania. Western Pacific surveillance and response journal : WPSAR. 2013;4(2):8-10.

7. van den Hurk AF, Hall-Mendelin S, Pyke AT, Frentiu FD, McElroy K, Day A, et al. Impact of Wolbachia on infection with chikungunya and yellow fever viruses in the mosquito vector Aedes aegypti. PLoS neglected tropical diseases. 2012;6(11):e1892.

8. Lo Presti A, Ciccozzi M, Cella E, Lai A, Simonetti FR, Galli M, et al. Origin, evolution, and phylogeography of recent epidemic CHIKV strains. Infection, genetics and evolution : journal of molecular epidemiology and evolutionary genetics in infectious diseases. 2012;12(2):392-8.

9. Vijayakumar KP, Nair Anish TS, George B, Lawrence T, Muthukkutty SC, Ramachandran R. Clinical Profile of Chikungunya Patients during the Epidemic of 2007 in Kerala, India. Journal of global infectious diseases. 2011;3(3):221-6.

10. Vijayakumar K ATS, Sreekala K.N., Ramachandran Reshmi, Philip Rekha Rachel. Environmental factors of households in five districts of Kerala affected by the epidemic of chikungunya fever in 2007. THE NATIONAL MEDICAL JOURNAL OF INDIA. 2010;23(2).

11. Andrew F. van den Hurk SH-M, Alyssa T. Pyke,, Greg A. Smith aJSM. Vector Competence of Australian Mosquitoes for Chikungunya Virus. VECTOR-BORNE AND ZOONOTIC DISEASES. 2010;10(5).

12. Tsetsarkin KA, Vanlandingham DL, McGee CE, Higgs S. A single mutation in chikungunya virus affects vector specificity and epidemic potential. PLoS pathogens. 2007;3(12):e201.

13. Chen TH, Jian SW, Wang CY, Lin C, Wang PF, Su CL, et al. Susceptibility of Aedes albopictus and Aedes aegypti to three imported Chikungunya virus strains, including the E1/226V variant in Taiwan. Journal of the Formosan Medical Association = Taiwan yi zhi. 2015;114(6):546-52.

14. Yoshikawa Minako Jen, Tang Choon Siang, Nishibuchi Mitsuaki. Incidence of Chikungunya Fever in Singapore: Implications of Public Health Measures and Transnational Movements of People Tropical medicine and health. 2010;38(1):39-45.

15. Li XF, Jiang T, Deng YQ, Zhao H, Yu XD, Ye Q, et al. Complete genome sequence of a chikungunya virus isolated in Guangdong, China. J Virol. 2012;86(16):8904-5.

16. Malik MR, Mnzava A, Mohareb E, Zayed A, Al Kohlani A, Thabet AA, et al. Chikungunya outbreak in Al-Hudaydah, Yemen, 2011: epidemiological characterization and key lessons learned for early detection and control. Journal of epidemiology and global health. 2014;4(3):203-11.

17. Moyen N, Thiberville SD, Pastorino B, Nougairede A, Thirion L, Mombouli JV, et al. First reported chikungunya fever outbreak in the republic of Congo, 2011. PloS one. 2014;9(12):e115938.

18. Narong Nitatpattana KK, Sutee Yoksan, Wichai Satimai, Narong Vongba,, Sasiporn Langdatsuwan KN, Supot Ratchakum1, Nadia Wauquier, Marc Souris,, Gonzalez PAaJ-P. Long-term persistence of Chikungunya virus neutralizing antibodies in human populations of North Eastern Thailand. Virology journal. 2014;11(183).

19. Nkoghe D, Kassa RF, Caron M, Grard G, Mombo I, Bikie B, et al. Clinical forms of chikungunya in Gabon, 2010. PLoS neglected tropical diseases. 2012;6(2):e1517.

20. Anubis Vega-Rúa KZ, Romain Girod, Anna-Bella Failloux, Ricardo Lourenço-de-Oliveira. High Level of Vector Competence of Aedes aegypti and Aedes albopictus from Ten American Countries as a Crucial Factor in the Spread of Chikungunya Virus. Journal of Virology. 2014;88(11):6294-306.

21. Thiberville SD, Boisson V, Gaudart J, Simon F, Flahault A, de Lamballerie X. Chikungunya fever: a clinical and virological investigation of outpatients on Reunion Island, South-West Indian Ocean. PLoS neglected tropical diseases. 2013;7(1):e2004.

22. Reller ME, Akoroda U, Nagahawatte A, Devasiri V, Kodikaarachchi W, Strouse JJ, et al. Chikungunya as a cause of acute febrile illness in southern Sri Lanka. PloS one. 2013;8(12):e82259.

23. Pfeffer M HI, Löscher T, Homeier T, Dobler G. Chikungunya fever in two German tourists returning from the Maldives, September, 2009. Euro Surveill. 2010;15(13).

24. Pfeffer M, Zoller G, Essbauer S, Tomaso H, Behrens-Riha N, Loscher T, et al. Clinical and virological characterization of imported cases of Chikungunya fever. Wiener klinische Wochenschrift. 2008;120(19-20 Suppl 4):95-100.

25. Taraphdar D, Chatterjee S. Molecular characterization of chikungunya virus circulating in urban and rural areas of West Bengal, India after its re-emergence in 2006. Transactions of the Royal Society of Tropical Medicine and Hygiene. 2015;109(3):197-202.

26. Kam YW, Pok KY, Eng KE, Tan LK, Kaur S, Lee WW, et al. Sero-prevalence and cross-reactivity of chikungunya virus specific anti-E2EP3 antibodies in arbovirus-infected patients. PLoS neglected tropical diseases. 2015;9(1):e3445.

27. Masri Sembiring Maha NKS, Nur Ika Hariastuti S. Chikungunya Virus Mutation, Indonesia, 2011. Emerging Infectious Diseases. 2015;21(2).

28. Kumar P, Pok KY, Tan LK, Angela C, Leo YS, Ng LC. Development and evaluation of baculovirus-expressed Chikungunya virus E1 envelope proteins for serodiagnosis of Chikungunya infection. Journal of virological methods. 2014;206:67-75.

29. Tsetsarkin KA, Chen R, Yun R, Rossi SL, Plante KS, Guerbois M, et al. Multi-peaked adaptive landscape for chikungunya virus evolution predicts continued fitness optimization in Aedes albopictus mosquitoes. Nature communications. 2014;5:4084.

30. Nasamon Wanlapakorn, Thanunrat Thongmee, Piyada Linsuwanon, Paiboon Chattakul, Sompong Vongpunsawad, Sunchai Payungporn, et al. Chikungunya Outbreak in Bueng Kan Province, Thailand, 2013. Emerging Infectious Diseases. 2014;20(8).

31. Tun MM, Thant KZ, Inoue S, Nabeshima T, Aoki K, Kyaw AK, et al. Detection of east/central/south African genotype of chikungunya virus in Myanmar, 2010. Emerg Infect Dis. 2014;20(8):1378-81.

32. Stapleford KA, Coffey LL, Lay S, Borderia AV, Duong V, Isakov O, et al. Emergence and transmission of arbovirus evolutionary intermediates with epidemic potential. Cell host & microbe. 2014;15(6):706-16.

33. Chusri S, Siripaitoon P, Silpapojakul K, Hortiwakul T, Charernmak B, Chinnawirotpisan P, et al. Kinetics of chikungunya infections during an outbreak in Southern Thailand, 2008-2009. The American journal of tropical medicine and hygiene. 2014;90(3):410-7.

34. Christofferson RC CD, Wearing HJ, Mores CN Chikungunya Viral Fitness Measures within the Vector and Subsequent Transmission Potential. PLoS One. 2014;9(10).

35. Sasayama M, Benjathummarak S, Kawashita N, et al. Chikungunya virus was isolated in Thailand, 2010. *Virus Genes*. 2014;49(3):485-489.

36. Ciccozzi M, Lo Presti A, Cella E, Giovanetti M, Lai A, El-Sawaf G, et al. Phylogeny of Dengue and Chikungunya viruses in Al Hudayda governorate, Yemen. Infection, genetics and evolution : Journal of molecular epidemiology and evolutionary genetics in infectious diseases. 2014;27:395-401.

37. Jyoti S. Kumar MP, P.V. Lakshmana Rao. Development & evaluation of biotinylated DNA probe for clinical diagnosis of chikungunya infection in patients’ acute phase serum & CSF samples. The Indian journal of medical research. 2013;138:117-24.

38. Horwood PF, Reimer LJ, Dagina R, Susapu M, Bande G, Katusele M, et al. Outbreak of chikungunya virus infection, Vanimo, Papua New Guinea. Emerg Infect Dis. 2013;19(9):1535-8.

39. Wu D, Zhang Y, Zhouhui Q, Kou J, Liang W, Zhang H, et al. Chikungunya virus with E1-A226V mutation causing two outbreaks in 2010, Guangdong, China. Virology journal. 2013;10:174.

40. Chen KC, Kam YW, Lin RT, Ng MM, Ng LF, Chu JJ. Comparative analysis of the genome sequences and replication profiles of chikungunya virus isolates within the East, Central and South African (ECSA) lineage. Virology journal. 2013;10:169.

41. Blagrove MS, Arias-Goeta C, Di Genua C, Failloux AB, Sinkins SP. A Wolbachia wMel transinfection in Aedes albopictus is not detrimental to host fitness and inhibits Chikungunya virus. PLoS neglected tropical diseases. 2013;7(3):e2152.

42. Arias-Goeta C, Mousson L, Rougeon F, Failloux AB. Dissemination and transmission of the E1-226V variant of chikungunya virus in Aedes albopictus are controlled at the midgut barrier level. PloS one. 2013;8(2):e57548.

43. Vazeille M, Yebakima A, Lourenco-de-Oliveira R, Andriamahefazafy B, Correira A, Rodrigues JM, et al. Oral receptivity of Aedes aegypti from Cape Verde for yellow fever, dengue, and chikungunya viruses. Vector borne and zoonotic diseases. 2013;13(1):37-40.

44. Parveen Kaur MT, Regina Ching Hua Lee, Huixin Chen, Karen Caiyun Chen, Mah Lee Ng,, Chua JJH. Inhibition of Chikungunya Virus Replication by Harringtonine, a Novel Antiviral That Suppresses Viral Protein Expression. Antimicrobial agents and chemotherapy. 2013;57(1):155-67.

45. Dupont-Rouzeyrol M, Caro V, Guillaumot L, Vazeille M, D'Ortenzio E, Thiberge JM, et al. Chikungunya virus and the mosquito vector Aedes aegypti in New Caledonia (South Pacific Region). Vector borne and zoonotic diseases. 2012;12(12):1036-41.

46. Lu X, Li X, Mo Z, Jin F, Wang B, Zhao H, et al. Rapid identification of Chikungunya and Dengue virus by a real-time reverse transcription-loop-mediated isothermal amplification method. The American journal of tropical medicine and hygiene. 2012;87(5):947-53.

47. Duong V, Andries AC, Ngan C, Sok T, Richner B, Asgari-Jirhandeh N, et al. Reemergence of Chikungunya virus in Cambodia. Emerg Infect Dis. 2012;18(12):2066-9.

48. Nabil Haddad LM, Marie Vazeille, Soulaima Chamat, Joelle Tayeh, Mike Abboud Osta, Failloux aA-B. Aedes albopictus in Lebanon, a potential risk of arboviruses outbreak. BMC Infectious Diseases. 2012;12(300).

49. Stephane Tchankouo-Nguetcheu EB, Pascal Lenormand, Jean-Claude Rousselle,, Choumet ANaV. Infection by chikungunya virus modulates the expression of several proteins in Aedes aegypti salivary glands. Parasites & vectors. 2012;5(264).

50. Singh RK, Tiwari S, Mishra VK, Tiwari R, Dhole TN. Molecular epidemiology of Chikungunya virus: mutation in E1 gene region. Journal of virological methods. 2012;185(2):213-20.

51. Kumar NP, Sabesan S, Krishnamoorthy K, Jambulingam P. Detection of Chikungunya virus in wild populations of Aedes albopictus in Kerala State, India. Vector borne and zoonotic diseases. 2012;12(10):907-11.

52. Kumar M, Sudeep AB, Arankalle VA. Evaluation of recombinant E2 protein-based and whole-virus inactivated candidate vaccines against chikungunya virus. Vaccine. 2012;30(43):6142-9.

53. Khan M, Dhanwani R, Rao PV, Parida M. Subunit vaccine formulations based on recombinant envelope proteins of Chikungunya virus elicit balanced Th1/Th2 response and virus-neutralizing antibodies in mice. Virus research. 2012;167(2):236-46.

54. Vilain P, Larrieu S, Renault P, Baville M, Filleul L. How to explain the re-emergence of chikungunya infection in Reunion Island in 2010? Acta tropica. 2012;123(2):85-90.

55. Das B, Sahu A, Das M, Patra A, Dwibedi B, Kar SK, et al. Molecular investigations of chikungunya virus during outbreaks in Orissa, Eastern India in 2010. Infection, genetics and evolution : journal of molecular epidemiology and evolutionary genetics in infectious diseases. 2012;12(5):1094-101.

56. Zouache K, Michelland RJ, Failloux AB, Grundmann GL, Mavingui P. Chikungunya virus impacts the diversity of symbiotic bacteria in mosquito vector. Molecular ecology. 012;21(9):2297-309.

57. Raharimalala FN, Ravaomanarivo LH, Ravelonandro P, Rafarasoa LS, Zouache K, Tran-Van V, et al. Biogeography of the two major arbovirus mosquito vectors, Aedes aegypti and Aedes albopictus (Diptera, Culicidae), in Madagascar. Parasites & vectors. 2012;5:56.

58. Bellini R, Medici A, Calzolari M, Bonilauri P, Cavrini F, Sambri V, et al. Impact of Chikungunya virus on Aedes albopictus females and possibility of vertical transmission using the actors of the 2007 outbreak in Italy. PloS one. 2012;7(2):e28360.

59. Ray P, Ratagiri VH, Kabra SK, Lodha R, Sharma S, Sharma BS, et al. Chikungunya infection in India: results of a prospective hospital based multi-centric study. PloS one. 2012;7(2):e30025.

60. Tsetsarkin KA, Weaver SC. Sequential adaptive mutations enhance efficient vector switching by Chikungunya virus and its epidemic emergence. PLoS pathogens. 2011;7(12):e1002412.

61. Sumathy K, Ella KM. Genetic diversity of Chikungunya virus, India 2006-2010: evolutionary dynamics and serotype analyses. Journal of medical virology. 2012;84(3):462-70.

62. Suwannakarn K, Theamboonlers A, Poovorawan Y. Molecular genome tracking of East, Central and South African genotype of Chikungunya virus in South–east Asia between 2006 and 2009. Asian Pacific Journal of Tropical Medicine. 2011;4(7):535-40.

63. Konstantin A. Tsetsarkin RC, Grace Leal, Naomi Forrester, Stephen Higgs, Jing Huang, and Scott C. Weaver. Chikungunya virus emergence is constrained in Asia by lineage-specific adaptive landscapes. PNAS. 2011;108(19):7872-7.

64. Sudeep AB, Hundekar SL, Jacob PG, Balasubramanian R, Arankalle VA, Mishra AC. Investigation of a Chikungunya-like illness in Tirunelveli district, Tamil Nadu, India 2009-2010. Tropical medicine & international health : TM & IH. 2011;16(5):585-8.

65. Tan CH, Wong PS, Li MZ, Tan SY, Lee TK, Pang SC, et al. Entomological investigation and control of a chikungunya cluster in Singapore. Vector borne and zoonotic diseases. 2011;11(4):383-90.

66. Ditsuwan T, Liabsuetrakul T, Chongsuvivatwong V, Thammapalo S, McNeil E. Assessing the spreading patterns of dengue infection and chikungunya fever outbreaks in lower southern Thailand using a geographic information system. Annals of epidemiology. 2011;21(4):253-61.

67. Ho K, Ang LW, Tan BH, Tang CS, Ooi PL, James L, et al. Epidemiology and control of chikungunya fever in Singapore. The Journal of infection. 2011;62(4):263-70.

68. Eric D’Ortenzio, Marc Grandadam, Elsa Balleydier M-C, Jaffar-Bandjee AM, Elise Brottet MB, Filleul aL. A226V Strains of Chikungunya Virus, Réunion Island, 2010. Emerging Infectious Diseases. 2011;17(2).

69. Girod R, Gaborit P, Marrama L, Etienne M, Ramdini C, Rakotoarivony I, et al. High susceptibility to Chikungunya virus of Aedes aegypti from the French West Indies and French Guiana. Tropical medicine & international health : TM & IH. 2011;16(1):134-9.

70. Chang SF, Su CL, Shu PY, Yang CF, Liao TL, Cheng CH, et al. Concurrent isolation of chikungunya virus and dengue virus from a patient with coinfection resulting from a trip to Singapore. Journal of clinical microbiology. 2010;48(12):4586-9.

71. Rianthavorn P, Prianantathavorn K, Wuttirattanakowit N, Theamboonlers A, Poovorawan Y. An outbreak of chikungunya in southern Thailand from 2008 to 2009 caused by African strains with A226V mutation. International journal of infectious diseases : IJID : official publication of the International Society for Infectious Diseases. 2010;14 Suppl 3:e161-5.

72. Pongsiri P, Auksornkitti, V., Theamboonlers, A., Luplertlop, N., Rianthavorn, P.,, Poovorawan Y. Entire genome characterization of Chikungunya virus from the 2008-2009 outbreaks in Thailand. Tropical Biomedicine. 2010;27(2):167–76.

73. Niyas KP, Abraham R, Unnikrishnan RN, Mathew T, Nair S, Manakkadan A, et al. Molecular characterization of Chikungunya virus isolates from clinical samples and adult Aedes albopictus mosquitoes emerged from larvae from Kerala, South India. Virology journal. 2010;7:189.

74. Tsetsarkin KA, McGee CE, Volk SM, Vanlandingham DL, Weaver SC, Higgs S. Epistatic roles of E2 glycoprotein mutations in adaption of chikungunya virus to Aedes albopictus and Ae. aegypti mosquitoes. PloS one. 2009;4(8):e6835.

75. Dubrulle M, Mousson L, Moutailler S, Vazeille M, Failloux AB. Chikungunya virus and Aedes mosquitoes: saliva is infectious as soon as two days after oral infection. PloS one. 2009;4(6):e5895.

76. Yap G, Pok KY, Lai YL, Hapuarachchi HC, Chow A, Leo YS, et al. Evaluation of Chikungunya diagnostic assays: differences in sensitivity of serology assays in two independent outbreaks. PLoS Neglected Tropical Diseases. 2010;4(7):e753.

77. Vazeille M, Moutailler S, Coudrier D, Rousseaux C, Khun H, Huerre M, et al. Two Chikungunya isolates from the outbreak of La Reunion (Indian Ocean) exhibit different patterns of infection in the mosquito, Aedes albopictus. PloS one. 2007;2(11):e1168.

78. Pages F, Peyrefitte CN, Mve MT, Jarjaval F, Brisse S, Iteman I, et al. Aedes albopictus mosquito: the main vector of the 2007 Chikungunya outbreak in Gabon. PloS one. 2009;4(3):e4691.

79. Vazeille M, Mousson L, Martin E, Failloux AB. Orally co-Infected Aedes albopictus from La Reunion Island, Indian Ocean, can deliver both dengue and chikungunya infectious viral particles in their saliva. PLoS neglected tropical diseases. 2010;4(6):e706.

80. Auksornkitti V, Pongsiri, P., Theamboonlers, A., Rianthavorn, P., Poovorawan Y. Whole-genome characterisation of Chikungunya virus from Aedes albopictus collected in Thailand. Annals of Tropical Medicine & Parasitology. 2010;104(3):265-9.

81. Christophe Paupy BO, Basile Kamgang, Sara Moutailler, Dominique Rousset,, Maurice Demanou J-PH, Eric Leroy, and Frédéric Simard. Comparative Role of Aedes albopictus and Aedes aegypti in the Emergence of Dengue and Chikungunya in Central Africa. Vector-borne and Zoonotic Diseases. 2010;10(3).

82. Catherine J. Westbrook MHR, Kendra N. Pesko,, Krystle E. Greene LPL. Larval Environmental Temperature and the Susceptibility of Aedes albopictus Skuse (Diptera: Culicidae) to Chikungunya Virus. Vector-borne and Zoonotic Diseases. 2010;10(3):241-7.

83. Mousson L, Martin E, Zouache K, Madec Y, Mavingui P, Failloux AB. Wolbachia modulates Chikungunya replication in Aedes albopictus. Molecular ecology. 2010;19(9):1953-64.

84. Hapuarachchi HC, Bandara KB, Sumanadasa SD, Hapugoda MD, Lai YL, Lee KS, et al. Re-emergence of Chikungunya virus in South-east Asia: virological evidence from Sri Lanka and Singapore. The Journal of general virology. 2010;91(Pt 4):1067-76.

85. Martin E, Moutailler S, Madec Y, Failloux AB. Differential responses of the mosquito Aedes albopictus from the Indian Ocean region to two chikungunya isolates. BMC ecology. 2010;10:8.

86. Zheng K, Li J, Zhang Q, Liang M, Li C, Lin M, et al. Genetic analysis of chikungunya viruses imported to mainland China in 2008. Virology journal. 2010;7:8.

87. Talbalaghi A, Moutailler, S., Vazeille, M., Failloux, A.B. Are Aedes albopictus or other mosquito species from northern Italy competent to sustain new arboviral outbreaks? Medical and veterinary entomology. 2010;24:83-7.

88. Sreekumar E, Issac A, Nair S, Hariharan R, Janki MB, Arathy DS, et al. Genetic characterization of 2006-2008 isolates of Chikungunya virus from Kerala, South India, by whole genome sequence analysis. Virus genes. 2010;40(1):14-27.

89. Moreira LA, Iturbe-Ormaetxe I, Jeffery JA, Lu G, Pyke AT, Hedges LM, et al. A Wolbachia symbiont in Aedes aegypti limits infection with dengue, Chikungunya, and Plasmodium. Cell. 2009;139(7):1268-78.

90. Jyh-Hsiung Huang, Cheng-Fen Yang C-LS, Shu-Fen Chang C-HC, Sheng-Kai Yu C-CL, Shu aP-Y. Imported Chikungunya Virus Strains, Taiwan, 2006–2009. Emerging Infectious Diseases. 2009;15(11).

91. Ng LC, Tan LK, Tan CH, Tan SS, Hapuarachchi HC, Pok KY, et al. Entomologic and virologic investigation of Chikungunya, Singapore. Emerg Infect Dis. 2009;15(8):1243-9.

92. Leroy EM, Nkoghe D, Ollomo B, Nze-Nkogue C, Becquart P, Grard G, et al. Concurrent chikungunya and dengue virus infections during simultaneous outbreaks, Gabon, 2007. Emerg Infect Dis. 2009;15(4):591-3.

93. Santhosh SR, Dash PK, Parida M, Khan M, Rao PV. Appearance of E1: A226V mutant Chikungunya virus in Coastal Karnataka, India during 2008 outbreak. Virology journal. 2009;6:172.

94. Vazeille M, Mousson, Laurence., Failloux Anna-Bella. Failure to demonstrate experimental vertical transmission of the epidemic strain of Chikungunya virus in Aedes albopictus from

La Réunion Island, Indian Ocean. Mem Inst Oswaldo Cruz, Rio de Janeiro. 2009;104(4):632-5.

95. Sam, I-Ching; Chan, Yoke Fun; Chan, Shie Yien; Loong, Shih Keng; Chin, Hock Khim; Hooi, Poh Sim; Ganeswrie, Rajasekaram; Abubakar, Sazaly. Chikungunya virus of Asian and Central/East African genotypes in Malaysia. Journal of Clinical Virology. 2009;46:180–3.

96. Moutailler S, Barre H, Vazeille M, Failloux AB. Recently introduced Aedes albopictus in Corsica is competent to Chikungunya virus and in a lesser extent to dengue virus. Tropical medicine & international health : TM & IH. 2009;14(9):1105-9.

97. Kowalzik S, Xuan NV, Weissbrich B, Scheiner B, Schied T, Drosten C, et al. Characterisation of a chikungunya virus from a German patient returning from Mauritius and development of a serological test. Medical microbiology and immunology. 2008;197(4):381-6.

98. Vazeille M, Moutailler S, Pages F, Jarjaval F, Failloux AB. Introduction of Aedes albopictus in Gabon: what consequences for dengue and chikungunya transmission? Tropical medicine & international health : TM & IH. 2008;13(9):1176-9.

99. Kumar NP, Joseph R, Kamaraj T, Jambulingam P. A226V mutation in virus during the 2007 chikungunya outbreak in Kerala, India. The Journal of general virology. 2008;89(Pt 8):1945-8.

100. Bordi L, Carletti F, Castilletti C, Chiappini R, Sambri V, Cavrini F, et al. Presence of the A226V mutation in autochthonous and imported Italian chikungunya virus strains. Clinical infectious diseases : an official publication of the Infectious Diseases Society of America. 2008;47(3):428-9.

101. Ratsitorahina M, Harisoa J, Ratovonjato J, Biacabe S, Reynes JM, Zeller H, et al. Outbreak of dengue and Chikungunya fevers, Toamasina, Madagascar, 2006. Emerg Infect Dis. 2008;14(7):1135-7.

102. Santhosh SR, Dash PK, Parida MM, Khan M, Tiwari M, Lakshmana Rao PV. Comparative full genome analysis revealed E1: A226V shift in 2007 Indian Chikungunya virus isolates. Virus research. 2008;135(1):36-41.

103. Panning M, Grywna, Klaus., van Esbroeck, Marjan., Emmerich, Petra., Drosten, Christian. Chikungunya Fever in Travelers Returning to Europe from the Indian Ocean Region, 2006. Emerging Infectious Diseases. 2008;14(3):416-22.

104. Vazeille M, Jeannin C, Martin E, Schaffner F, Failloux AB. Chikungunya: a risk for Mediterranean countries? Acta tropica. 2008;105(2):200-2.

105. Rezza G, Nicoletti L, Angelini R, Romi R, Finarelli AC, Panning M, et al. Infection with chikungunya virus in Italy: an outbreak in a temperate region. The Lancet. 2007;370(9602):1840-6.

106. Arankalle VA, Shrivastava S, Cherian S, Gunjikar RS, Walimbe AM, Jadhav SM, et al. Genetic divergence of Chikungunya viruses in India (1963-2006) with special reference to the 2005-2006 explosive epidemic. The Journal of general virology. 2007;88(Pt 7):1967-76.

107. Schuffenecker I, Iteman I, Michault A, Murri S, Frangeul L, Vaney MC, et al. Genome microevolution of chikungunya viruses causing the Indian Ocean outbreak. PLoS medicine. 2006;3(7):e263.

108. Arias-Goeta C, Moutailler S, Mousson L, Zouache K, Thiberge JM, Caro V, et al. Chikungunya virus adaptation to a mosquito vector correlates with only few point mutations in the viral envelope glycoprotein. Infection, genetics and evolution : journal of molecular epidemiology and evolutionary genetics in infectious diseases. 2014;24:116-26.

109. Scagnolari C CB, Rezza G, Antonelli G. Antiviral Activity of the Combination of Interferon and Ribavirin Against Chikungunya Virus: Are the Results Conclusive? J Infect Dis. 2017;215(3):492-3.

110. Mathieu JP Poirier DMM, Karla R Feeser,Thomas G Streit, Gwong-Jen J Chang, Matthew Whitney, Brandy J Russell, Barbara W Johnson, Alison J Basile, Christin H Goodman, Amanda K Barry, Lammie PJ. Measuring Haitian children's exposure to chikungunya, dengue and malaria. Bulletin of the World Health Organization. 2016;94:817-25.

111. Wang YM, Lu JW, Lin CC, Chin YF, Wu TY, Lin LI, et al. Antiviral activities of niclosamide and nitazoxanide against chikungunya virus entry and transmission. Antiviral research. 2016;135:81-90.

112. Yang CF, Su CL, Hsu TC, Chang SF, Lin CC, Huang JC, et al. Imported Chikungunya Virus Strains, Taiwan, 2006-2014. Emerg Infect Dis. 2016;22(11):1981-4.

113. Hugo LE, Prow NA, Tang B, Devine G, Suhrbier A. Chikungunya virus transmission between Aedes albopictus and laboratory mice. Parasites & vectors. 2016;9(1):555.

114. Chen R, Puri V, Fedorova N, Lin D, Hari KL, Jain R, et al. Comprehensive Genome Scale Phylogenetic Study Provides New Insights on the Global Expansion of Chikungunya Virus. J Virol. 2016;90(23):10600-11.

115. Rodas JD, Kautz T, Camacho E, Paternina L, Guzman H, Diaz FJ, et al. Genetic Characterization of Northwestern Colombian Chikungunya Virus Strains from the 2014-2015 Epidemic. The American journal of tropical medicine and hygiene. 2016;95(3):639-46.

116. Agarwal A, Sharma AK, Sukumaran D, Parida M, Dash PK. Two novel epistatic mutations (E1:K211E and E2:V264A) in structural proteins of Chikungunya virus enhance fitness in Aedes aegypti. Virology. 2016;497:59-68.

117. Saraswat S, Athmaram TN, Parida M, Agarwal A, Saha A, Dash PK. Expression and Characterization of Yeast Derived Chikungunya Virus Like Particles (CHIK-VLPs) and Its Evaluation as a Potential Vaccine Candidate. PLoS neglected tropical diseases. 2016;10(7):e0004782.

118. Vazeille M, Zouache K, Vega-Rua A, Thiberge JM, Caro V, Yebakima A, et al. Importance of mosquito "quasispecies" in selecting an epidemic arthropod-borne virus. Scientific reports. 2016;6:29564.

119. Conteville LC, Zanella L, Marin MA, Filippis AM, Nogueira RM, Vicente AC, et al. Phylogenetic analyses of chikungunya virus among travelers in Rio de Janeiro, Brazil, 2014-2015. Memorias do Instituto Oswaldo Cruz. 2016;111(5):347-8.

120. Chompoosri J, Thavara U, Tawatsin A, Boonserm R, Phumee A, Sangkitporn S, et al. Vertical transmission of Indian Ocean Lineage of chikungunya virus in Aedes aegypti and Aedes albopictus mosquitoes. Parasites & vectors. 2016;9:227.

121. Sy AK, Saito-Obata M, Medado IA, Tohma K, Dapat C, Segubre-Mercado E, et al. Molecular Characterization of Chikungunya Virus, Philippines, 2011-2013. Emerg Infect Dis. 2016;22(5):887-90.

122. Naresh Kumar CVM, Sivaprasad Y, Sai Gopal DVR. Genetic diversity of 2006–2009 Chikungunya virus outbreaks in Andhra Pradesh, India, reveals complete absence of E1:A226V mutation. Acta virologica. 2016;60(01):114-7.

123. Nyari N, Maan HS, Sharma S, Pandey SN, Dhole TN. Identification and genetic characterization of chikungunya virus from Aedes mosquito vector collected in the Lucknow district, North India. Acta tropica. 2016;158:117-24.

124. Gokhale MD, Paingankar MS, Sudeep AB, Parashar D. Chikungunya virus susceptibility & variation in populations of Aedes aegypti (Diptera: Culicidae) mosquito from India. The Indian journal of medical research. 2015;142 Suppl:S33-43.

125. Parashar D, Amdekar S, More A, Patil P, More R, Babu VR. Chikungunya fever outbreak in Guntur, Andhra Pradesh, India. The Indian journal of medical research. 2015;142 Suppl:S111-5.

126. Galatas B, Ly S, Duong V, Baisley K, Nguon K, Chan S, et al. Long-Lasting Immune Protection and Other Epidemiological Findings after Chikungunya Emergence in a Cambodian Rural Community, April 2012. PLoS neglected tropical diseases. 2016;10(1):e0004281.

127. Maurice D, Alain SM, Christophe V, Rene N, Irene KT, Marthe IN, et al. Molecular characterization of chikungunya virus from three regions of Cameroon. Virologica Sinica. 2015;30(6):470-3.

128. Wang C, Saborio S, Gresh L, Eswarappa M, Wu D, Fire A, et al. Chikungunya Virus Sequences Across the First Epidemic in Nicaragua, 2014-2015. The American journal of tropical medicine and hygiene. 2016;94(2):400-3.

129. Abraham R, Manakkadan A, Mudaliar P, Joseph I, Sivakumar KC, Nair RR, et al. Correlation of phylogenetic clade diversification and in vitro infectivity differences among Cosmopolitan genotype strains of Chikungunya virus. Infection, genetics and evolution : journal of molecular epidemiology and evolutionary genetics in infectious diseases. 2016;37:174-84.

130. Wintachai P, Kaur P, Lee RC, Ramphan S, Kuadkitkan A, Wikan N, et al. Activity of andrographolide against chikungunya virus infection. Scientific reports. 2015;5:14179.

131. Hinson JM, Dave S, McMenamy SS, Dave K, Turell MJ. Immuno-Chromatographic Wicking Assay for the Rapid Detection of Chikungunya Viral Antigens in Mosquitoes (Diptera: Culicidae). J Med Entomol. 2015;52(4):699-704.

132. Lani R, Hassandarvish P, Chiam CW, Moghaddam E, Chu JJ, Rausalu K, et al. Antiviral activity of silymarin against chikungunya virus. Scientific reports. 2015;5:11421.

133. Ahmadi A, Hassandarvish P, Lani R, Yadollahi P, Jokar A, Bakar SA, et al. Inhibition of chikungunya virus replication by hesperetin and naringenin. RSC Advances. 2016;6(73):69421-30.

134. Afreen N, Deeba F, Khan WH, Haider SH, Kazim SN, Ishrat R, et al. Molecular characterization of dengue and chikungunya virus strains circulating in New Delhi, India. Microbiology and immunology. 2014;58(12):688-96.

135. Sam, I.-C., Loong, S.-K., Michael, J. C., Chua, C.-L., Wan Sulaiman, W. Y., Vythilingam, I., … Chan, Y.-F. (2012). Genotypic and Phenotypic Characterization of Chikungunya Virus of Different Genotypes from Malaysia. *PLoS ONE*, *7*(11), e50476.

136. Mudurangaplar B, Peerapur BV. Molecular Characterisation of Clinical Isolates of Chikungunya Virus: A Study from Tertiary Care Hospitals in Southern India. Journal of clinical and diagnostic research : JCDR. 2016;10(3):DC14-7.

137. Raghavendhar BS, Ray P, Ratagiri VH, Sharma BS, Kabra SK, Lodha R. Evaluation of chikungunya virus infection in children from India during 2009-2010: A cross sectional observational study. Journal of medical virology. 2016;88(6):923-30.

138. Saswat T, Kumar A, Kumar S, Mamidi P, Muduli S, Debata NK, et al. High rates of co-infection of Dengue and Chikungunya virus in Odisha and Maharashtra, India during 2013. Infection, genetics and evolution : journal of molecular epidemiology and evolutionary genetics in infectious diseases. 2015;35:134-41.

139. Desdouits M, Kamgang B, Berthet N, Tricou V, Ngoagouni C, Gessain A, et al. Genetic characterization of Chikungunya virus in the Central African Republic. Infection, genetics and evolution : journal of molecular epidemiology and evolutionary genetics in infectious diseases. 2015;33:25-31.

140. Lanciotti RS, Lambert AJ. Phylogenetic Analysis of Chikungunya Virus Strains Circulating in the Western Hemisphere. The American journal of tropical medicine and hygiene. 2016;94(4):800-3.

141. Diaz-Quinonez JA, Escobar-Escamilla N, Ortiz-Alcantara J, Vazquez-Pichardo M, de la Luz Torres-Rodriguez M, Nunez-Leon A, et al. Identification of Asian genotype of chikungunya virus isolated in Mexico. Virus genes. 2016;52(1):127-9.

142. Lim CK, Nishibori T, Watanabe K, Ito M, Kotaki A, Tanaka K, et al. Chikungunya virus isolated from a returnee to Japan from Sri Lanka: isolation of two sub-strains with different characteristics. The American journal of tropical medicine and hygiene. 2009;81(5):865-8.

143. Sahu A, Das B, Das M, Patra A, Biswal S, Kar SK, et al. Genetic characterization of E2 region of Chikungunya virus circulating in Odisha, Eastern India from 2010 to 2011. Infection, genetics and evolution : journal of molecular epidemiology and evolutionary genetics in infectious diseases. 2013;18:113-24.

144. Roche B, Leger L, L'Ambert G, Lacour G, Foussadier R, Besnard G, et al. The Spread of Aedes albopictus in Metropolitan France: Contribution of Environmental Drivers and Human Activities and Predictions for a Near Future. PloS one. 2015;10(5):e0125600.

145. Grandadam M, Caro V, Plumet S, Thiberge JM, Souares Y, Failloux AB, et al. Chikungunya virus, southeastern France. Emerg Infect Dis. 2011;17(5):910-3.

146. Gardner CL, Hritz J, Sun C, Vanlandingham DL, Song TY, Ghedin E, et al. Deliberate attenuation of chikungunya virus by adaptation to heparan sulfate-dependent infectivity: a model for rational arboviral vaccine design. PLoS neglected tropical diseases. 2014;8(2):e2719.

147. Shrinet J, Jain S, Sharma A, Singh SS, Mathur K, Rana V, et al. Genetic characterization of Chikungunya virus from New Delhi reveal emergence of a new molecular signature in Indian isolates. Virology journal. 2012;9:100.

148. N. Pradeep Kumar1 MMM, N. Krishnamoorthy TK, Rajan Joseph,, Jambulingam P. Genotyping of virus involved in the 2006 Chikungunya outbreak in South India (Kerala and Puducherry). Current Science. 2007;93(10).

149. Singh P, Sharma P, Kumar S, Chhabra M, Rizvi MA, Mittal V, et al. Continued persistence of ECSA genotype with replacement of K211E in E1 gene of Chikungunya virus in Delhi from 2010 to 2014. Asian Pacific Journal of Tropical Disease. 2016;6(7):564-6.

150. Tan KK, Sy AK, Tandoc AO, Khoo JJ, Sulaiman S, Chang LY, et al. Independent Emergence of the Cosmopolitan Asian Chikungunya Virus, Philippines 2012. Scientific reports. 2015;5:12279.

151. Chua CL, Sam IC, Merits A, Chan YF. Antigenic Variation of East/Central/South African and Asian Chikungunya Virus Genotypes in Neutralization by Immune Sera. PLoS neglected tropical diseases. 2016;10(8):e0004960.

152. Pankaj Pal JMF, David W. Hawman, Yan-Jang S. Huang, Ilhem Messaoudi, Craig Kreklywich, Michael Denton,, Alfred W. Legasse PPS, Syd Johnson, Michael K. Axthelm, Dana L. Vanlandingham, Daniel N. Streblow,, Stephen Higgs TEM, Michael S. Diamond. Chikungunya Viruses That Escape Monoclonal Antibody Therapy Are Clinically Attenuated, Stable, and Not Purified in Mosquitoes. Journal of Virology. 2014;88(15):8213–26.

153. Stapleford KA, Moratorio G, Henningsson R, Chen R, Matheus S, Enfissi A, et al. Whole-Genome Sequencing Analysis from the Chikungunya Virus Caribbean Outbreak Reveals Novel Evolutionary Genomic Elements. PLoS neglected tropical diseases. 2016;10(1):e0004402.

154. Rubén Bueno Marí, Ricardo Jiménez Peydró. Current status and eco-epidemiology of mosquito-borne arboviruses (Diptera: Culicidae) in Spain. Rev Esp Salud Pública. 2010;84(3):255-69.

155. Sahadeo N, Mohammed H, Allicock OM, Auguste AJ, Widen SG, Badal K, et al. Molecular Characterisation of Chikungunya Virus Infections in Trinidad and Comparison of Clinical and Laboratory Features with Dengue and Other Acute Febrile Cases. PLoS neglected tropical diseases. 2015;9(11):e0004199.

156. Naresh Kumar CVM, Sangamithra P, Rajasekhar M, Saigopal DVR. Surveillance of chikungunya virus in Andhra Pradesh, Southern India. Asian Pacific Journal of Tropical Medicine. 2010;3(11):860-5.

157. Philippe Parola XdL, Jacques Jourdan, Clarisse Rovery,, Véronique Vaillant PM, Philippe Brouqui, Antoine Flahault, Didier Raoult,, Charrel aRN. Novel Chikungunya Virus Variant in Travelers Returning from Indian Ocean Islands. Emerging Infectious Diseases. 2006;12(10).

158. Fahmy NT, Klena JD, Mohamed AS, Zayed A, Villinski JT. Complete Genome Sequence of Chikungunya Virus Isolated from an Aedes aegypti Mosquito during an Outbreak in Yemen, 2011. Genome announcements. 2015;3(4).

159. Kautz TF, Diaz-Gonzalez EE, Erasmus JH, Malo-Garcia IR, Langsjoen RM, Patterson EI, et al. Chikungunya Virus as Cause of Febrile Illness Outbreak, Chiapas, Mexico, 2014. Emerg Infect Dis. 2015;21(11):2070-3.

160. Savage HM, Ledermann JP, Yug L, Burkhalter KL, Marfel M, Hancock WT. Incrimination of Aedes (Stegomyia) hensilli Farner as an epidemic vector of Chikungunya virus on Yap Island, Federated States of Micronesia, 2013. The American journal of tropical medicine and hygiene. 2015;92(2):429-36.

161. Moro ML, Gagliotti C, Silvi G, Angelini R, Sambri V, Rezza G, et al. Knowledge, attitudes and practices survey after an outbreak of chikungunya infections. International health. 2010;2(3):223-7.

162. Ooi MK, Gan HM, Rohani A, Syed Hassan S. First Complete Genome Sequence of a Chikungunya Virus Strain Isolated from a Patient Diagnosed with Dengue Virus Infection in Malaysia. Genome announcements. 2016;4(4).
