# Supplementary material for: A scoping review of published literature on chikungunya virus
Source: PLoS One. 2018 Nov 29;13(11):e0207554. doi: 10.1371/journal.pone.0207554 (PMC6264817; doi:10.1371/journal.pone.0207554)
Supplement: S2 Table — *Multiple diagnostic tests reported in many articles. (DOCX) [file pone.0207554.s005.docx]

**S2 Table: Diagnostic tests and samples for chikungunya testing for humans***

| *Diagnostic method* | *687 studies* |
| --- | --- |
|  |  |
| Clinical symptoms | 126 |
| Virus isolation and culture | 119 |
| Molecular identification test | 347 |
| Serological test | 576 |
|  |  |
| Clinical symptoms + Virus isolation | 14 |
| Clinical symptoms + Molecular tests | 10 |
| Clinical symptoms + Serology | 67 |
|  |  |
| Virus isolation + Molecular tests | 11 |
| Virus isolation + Serology | 92 |
| Virus isolation +Molecular tests + Serology | 11 |
| Molecular tests + Serology | 141 |
|  |  |
| Clinical symptoms + Virus isolation + Molecular tests + serology | 10 |
|  |  |
| *Diagnostic Sample* |  |
|  |  |
| Blood |  |
| Cerebrospinal fluid (CSF) | 52 |
| Hepatic biopsy tissue | 3 |
| Cord blood | 4 |
| Saliva | 3 |
| Urine | 7 |
| Semen | 2 |
| Corneas | 1 |
| Aqueous humor | 3 |
| Villus biopsy | 1 |
| Amniotic fluid | 2 |
| Placentas | 4 |
| Fetal brain tissues | 2 |
| Blister fluids | 4 |
| Peritoneal fluid | 1 |
| Eye tissue | 1 |
| Skin tissue | 3 |
| Other non-specified tissues | 1 |
|  |  |

*Multiple diagnostic tests reported in many articles
