# Supplementary material for: A scoping review of published literature on chikungunya virus
Source: PLoS One. 2018 Nov 29;13(11):e0207554. doi: 10.1371/journal.pone.0207554 (PMC6264817; doi:10.1371/journal.pone.0207554)
Supplement: S3 Table — *Multiple tests evaluated in many articles. (DOCX) [file pone.0207554.s006.docx]

**S3 Table: Details from 167/1920 articles on the evaluation of CHIKV diagnostic tests***

| Diagnostic test evaluated | # articles | Sensitivity evaluated | Specificity evaluated | Comparison to other tests |
| --- | --- | --- | --- | --- |
| Clinical signs and symptoms | 16 | 4 | 4 | 3 |
| Virus culture and identification | 15 | 2 | 1 | 2 |
| Serological tests (n=90) | | | | |
| *Enzyme-linked immunosorbant assay (ELISA)* | 59 | 22 | 19 | 24 |
| *Immunofluorescent-antibody assays (IFA)* | 21 | 1 | 1 | 1 |
| *Hemagglutinin/inhibition assay (HI)* | 11 | 0 | 0 | 1 |
| *Microneutralization tests (MNT)* | 6 | 0 | 0 | 1 |
| *Immunochromatographic tests (ICT)* | 6 | 2 | 2 | 2 |
| *Rapid detection tests (RDT)* | 9 | 9 | 9 | 9 |
| *Plaque/Focus reduction neutralization tests (F/PRNT)* | 18 | 0 | 0 | 0 |
| *Western blot* | 3 | 1 | 1 | 0 |
| *Complement fixation* | 3 | 0 | 0 | 0 |
| Molecular tests (n=88) | | | | |
| *Reverse transcription PCR*  *( RT-PCR)* | 39 | 31 | 26 | 26 |
| *Quantitative RT-PCR (qRT-PCR) /*  *Real time RT-PCR (rRT/PCR)* | 26 | 24 | 20 | 19 |
| *Nested RT-PCR* | 3 | 1 | 1 | 0 |
| *Loop-mediated isothermal amplification (LAMP)* | 4 | 2 | 2 | 1 |

*Multiple tests evaluated in many articles
