# Supplementary material for: A scoping review of published literature on chikungunya virus
Source: PLoS One. 2018 Nov 29;13(11):e0207554. doi: 10.1371/journal.pone.0207554 (PMC6264817; doi:10.1371/journal.pone.0207554)
Supplement: S4 Table — (DOCX) [file pone.0207554.s007.docx]

| ***Reference*** | ***Time of illness / sampling*** | ***Publication*** | ***Country*** | ***Genotype*** | ***Hyperpigmentation*** | ***Ocular complications*** | ***Neurological complications*** |
| --- | --- | --- | --- | --- | --- | --- | --- |
| [1] | 1964 | 1969 | India |  |  |  | 2 patients had residual neurological deficits on discharge from the hospital. |
| [2] | 1964 | 1965 | India |  |  |  | One case with prominent external opthalmoplegia, another with polyneuropathy and a third with transient slurring of speech |
| [3] | July 1968 to November 1969 | 1971 | Cambodia |  |  |  | Acute encephalopathy in 1 case with hyperglycorrhachia associated with torpor in another, acute lymphocytic meningitis |
| [4] | 1988 - 1990 | 1992 | China |  |  |  | Viral encephalitis - 5/614 |
| [5] | 2005 - 2006 | 2009 | Reunion - Mayotte |  | 1/610 cases with hyperpigmentation of the nose |  | Encephalitis in 69/610,meningoencephalitis in 15/610, and myelomeningo-encephalitis in 1/610. Of 147 cases with neurological disorders, 25 [17%] had underlying neurological conditions. |
| [6] | 2005 - 2006 | 2008 | Reunion |  | Hyperpigmentation in 5% of the pregnant mothers studied |  | Encephalitis 99/834, meningo-encephalitis in pregnant mothers - 20%, convulsions 63/834 |
| [7] | 2005 - 2006 | 2008 | Reunion - Pediatric cases |  |  |  | Meningitis syndrome in approximately 11% approximated from graph presented |
| [8] | 2005 - 2006 | 2007 | Reunion |  | Patients with persistent dyschromatic patches on skin |  |  |
| [9] | 2005 - 2006 | 2007 | Reunion |  |  |  | Meningo-encephalitis in 16/123, other central nervous system problem 7/123 |
| [10] | 2005 - 2009 | 2016 | Reunion - cohort study for CHIKV associated encephalitis |  |  |  | 6/57 encephalitis, general or partial seizures-4/57; facial neurological signs-5/57 |
| [11] | 2005 to 2006 | 2008 | Reunion - Mother-to-child transmission |  |  |  | Encephalopathy - 9/19 in neonates with severe disease |
| [12] | April 2005 to May 2007 | 2009 | France - All travellers with suspected CHIKV infection |  |  |  | Nervous tunnel syndrome in 3/51 |
| [13] | August 2005 to May 2006 | 2008 | Reunion Island |  |  |  | Encephalopathy 14/33, 1/33 Guillaine Barré syndrome, neurologic signs were hyporeflexia [patient 9], hyperreflexia [patient 12], asterixis [patients 6 and 10], mydriasis [patients 5 and 7]; and nystagmus [patient 7]./ total of 33 |
| [14] | December 2005 - January 2006 | 2006 | Reunion |  |  |  | Meningoencephalitis |
| [15] | December 2005 to April 2006 | 2007 | Reunion |  | 27/31 patients had hyperpigmentation occur on the nose, face, limbs or trunk, 3/31 over the entire body | 9/15 uveitis |  |
| [16] | June 2005 to Jan 2006 | 2006 | Reunion - vertical maternal-fetal transmission |  |  |  | 4/10 neonates meningoencephalitis, 10/10 Newborns - convulsions |
| [17] | March 2005 - Jan 2006 | 2006 | Reunion |  |  |  | Meningoencephalitis in 1.7/1000 |
| [18] | March 2005 outbreak | 2006 | Reunion | ECSA [Indian Ocean Lineage] |  |  | Encephalitic forms were reported on many occasions during the active phase of the outbreak |
| [19] | March 2005 to April 2006 | 2009 | Reunion |  |  |  | Two cases of polyradiculoneuritis. Guillain-Barré 2/21; confusion syndrome: 20/21; epilepsy or seizure: 6/21; meningeal syndrome: 1/21; motor deficit: 1/21; sensorial deficit: 1/21 |
| [20] | March 2005 to April 2006 | 2007 | Reunion - 38 neonates with vertrically transmitted CHIKV infection within fist week of birth |  | Neonates - 31/38 had a brownish dyschromia of the limbs and sometimes the face |  | Neonates - Seizures 6/38 [16%] |
| [21] | March 2005 to January 2006 | 2006 | Reunion |  |  |  | 15 /10750 cases of meningoencephalitis |
| [22] | May 2005 to May 2006 | 2011 | Reunion |  |  |  | Meningoencephalitis in 5/43; |
| [23] | 2006 | 2007 | India | ECSA |  | 1/74 visual failure. | Encephalopathy was observed in 35 cases [47.2%] and was the isolated neurologic finding in 11 cases [14.9%] and the most prominent feature in 33. Altered sensorium 5 patients [14.7%] : ataxia in 4, focal rigidity in 1 and opsoclonus in 2, coma, limb paralysis |
| [24] | 2006 | 2011 | India |  |  |  | CNS symptoms 40/740 |
| [25] | 2006 | 2009 | India |  |  |  | Encephalopathy - 6/87 [6.9%] in confirmed cases and 5/318 [1.5%] in suspected cases, Guillain Barré Syndrome - 2/318 [0.4%] |
| [26] | 2006 | 2009 | Reunion - |  |  |  | Polyradiculoneuropathy 1/1, 2/2 Guillain Barré Syndrome |
| [27] | 2006 | 2009 | India | ECSA |  |  | 30 /99 [30.3%] were exclusively neurologic. Neurologic manifestations included altered sensorium [50], unconsciousness [27], seizures [16], irritability [1], neck stiffness [4], speech disturbances [5], hypotonia [4], paralysis [12], abnormal deep tendon reflexes [10] and abnormal plantar responses [14]. Encephalitis [57] and encephalopathy [42] were noted with additional myelopathy [14] or myeloneuropathy [12]. |
| [28] | Apr-2006 | 2008 | Reunion - retrospective study in hospital staff |  |  |  | Neurological signs 7 %, Concentration difficulty 84. 7 % ,Hallucinations 9. 5% |
| [29] | August to October 2006 | 2007 | India |  |  | 2/20 blindness due to retro-bulbar neuritis | 20/20 altered level of consciousness [confusion, disorientation, drowsiness and delirium], 6/20 seizures, 1/20 hemiparesis, 3/20 paraplegia, 4/20 involuntary movements/upper limbs |
| [30] | Feb - June 2006 | 2008 | Children in Mayotte |  |  |  | 46% [meningitis-like syndrome in 18%, convulsion in 16%] |
| [31] | Jan to April 2006 | 2012 | IOI |  |  |  | 4/9/encephalopathy, |
| [32] | Jan to May 2006 | 2008 | Children in Reunion |  |  |  | 12/30 [20%] encephalitis, 4/30 [13%] acute encephalopathies, seizure 16/30 [53%], 30/122 [25%] neurologic manifestations. 1/30 [3%] had persistent neurodevelopmental delay 6 months after discharge with microcephaly and strabismus. 1/30 [3%] had recurrent seizures |
| [33] | Jan to October 2006 | 2009 | India - Study in children looking for CHIKV and neurological symptoms | ECSA | Gangrene development in fingers and toes in 1 child |  | Seizures 7/11 [64%], focal neurologic signs 14% |
| [34] | Jan to Sept 2006 | 2009 | India |  | 12/115 patients showed grouped, hyperpigmented macules coalescing in some areas, distributed predominantly over the nose and cheeks. 5/115patients showed discrete, scattered hyperpigmented macules over the face and trunk. |  |  |
| [35] | July to Dec 2006 | 2010 | India |  |  |  | 4/9 encephalitis in confirmed cases, 20/37 encephalitis in suspected cases, 46/300/neurological complications |
| [36] | June - December 2006 | 2009 | India |  |  |  | 49/300 neurological symptoms, encephalitis in 27/49, myelopathy in 7/49, myelo-neuropathy in 7/49, peripheral neuropathy in 7/49, and myopathy in 1/49. |
| [37] | May to July 2006 | 2008 | India |  | Pigmentary changes in 61/145 [42%] of subjects across all ages |  |  |
| [38] | Sept to Nov 2006 | 2007 | India - retrospective patient chart analysis |  |  | Nongranulomatous anterior uveitis [10/37], Panuveitis [5/37], Granulomatous anterior uveitis [1/37], Optic neuritis [4/37], Lagophthalmos and VIth nerve palsy [3/37], Retrobulbar neuritis [3/37], Retinitis with vitreitis[2/37], Bilateral neuroretinitis[1/37], Keratitis [3/37], CRAO [1/37], Multifocal choroiditis with CME[2/37],Exudative retinal detachment [2/37] |  |
| [39] | September to October 2006 | 2007 | India -case series with Chik associated optic neuritis |  |  | 14/14 optic neuritis |  |
| [40] | 2007 | 2008 | India - Chik in neonates |  | Neonates showed diffuse deep hyperpigmentation, especially over the face, nose and also over abdomen, extremities and knuckles. |  | Occasional seizures, shock and disseminated intravascular coagulation were seen in a few cases |
| [41] | 2007 | 2009 | India |  |  |  | Encephalitis 1/35, febrile seizure in children between 6 to 12 months, and 1 to 5 years suspected cases [80%]. |
| [42] | 2007 | 2008 | Italy |  |  |  | Encephalitis 1/1, perturbation of cognition and level of consciousness, 1/1 |
| [43] | April to October 2007 | 2009 | Sri Lanka - study on pregnant mothers and neonates |  | Neonates - hyperpigmentation in 9/30 healthy newborns whose mothers were infected in different trimesters |  | 1/4 infants born to mothers with infection - with meningoencephalitis |
| [44] | July to August 2007 | 2011 | India |  |  | 10/10 decrease in vision or blurring of vision with sudden onset, 7/10 unilateral optic neuritis, 3/10 bilateral optic neuritis |  |
| [45] | June to December 2007 | 2011 | India |  |  |  | 3 patients had altered sensorium and one patient had *ADEM [acute disseminated encephalomyelitis]*. |
| [46] | May to July 2007 | 2009 | India - study in infants |  | Infants <1 year with CHIKV infection - one infant with chikungunya with extensive peeling of skin, with co-existing hypo-pigmentation and hyper pigmentation of skin, in 39 infants [69.69%] lesions healed leaving hyperpigmented scars while 13 [23.21%] had hypopigmented lesions. |  |  |
| [47] | Jul-2008 | 2010 | Singapore |  |  |  | 1/2 - suggested encephalitis, seizures [1.6%] |
| [48] | June 2008 to April 2009 | 2010 | India |  |  |  | Neuritis [24%] |
| [49] | June to August 2008 | 2011 | India |  | Pigmentary changes - 12/75; Diffuse hyperpigmentation of face - 9/75 |  |  |
| [50] | May to July 2008 | 2011 | India |  |  |  | Numbness 5/180 |
| [51] | 2009 - 2010 | 2012 | India |  | 27/52 [51.9%] - Generalized pigmentary changes were the commonest in children whereas it was localized in adults in the form of centrofacial or melasma like pigmentation. |  | Meningoencephalitis 1/52 |
| [52] | April to July 2009 | 2012 | Thailand |  |  |  | Neurological complications - 12.5% |
| [53] | April to June 2009 | 2011 | Thailand - neuro Chik reports |  |  |  | 1/3 - Cranial MRI showed diffused brain atrophy and confluent and large hyperintense signals of periventricular white matter area 1/3 - Neurologic examination showed a conscious woman with facial diplegia and an areflexic grade 2/5 motor power on proximal muscles of the lower and upper extremities. |
| [54] | Dec 8 to Oct 2009 | 2011 | Thailand |  |  |  | 14%/neurological signs-dizziness, alteration of consciousness, weakness, convulsion/serologically confirmed, 62%/neurological signs-dizziness, alteration of consciousness, weakness, convulsion/virology confirmed |
| [55] | January 2009 to July 2012 | 2014 | India |  |  | 2/36 anterior uveitis, 2/36 keratic precipitates, 1/36 abnormal iris pattern, 1/36 moth eaten appearance iris, 1/36 increase in intraocular pressure |  |
| [56] | July to September 2009 | 2011 | India |  | Post-inflammatory hypopigmentation |  | Febrile seizure - 1/10 |
| [57] | July to September 2009 | 2010 | India |  | Hyperpigmentation in 38.27% of patients, mainly on nose. |  |  |
| [58] | 2010 | 2014 | Patient travelled to India and returned to Canada |  | 1/1/hyperpigmentation on bridge of the nose |  |  |
| [59] | 2010 | 2012 | India |  | 10/37, Hyperpigmentation of palms, feet and calves |  |  |
| [60] | August 2010 to Feb 2011 | 2011 | India |  |  |  | 1/148 meningoencephalitis |
| [61] | Jan 2010 to Dec 2010 | 2014 | India |  |  | 1/1/retinitis, 1/1/uveitis, 1/1/diminution of vision in both eyes, 1/1/relative afferent pupillary defect [RAPD] in the right eye, 1/1/macular edema, 1/1/serous detachment |  |
| [62] | November 2010 to March 2011 | 2015 | India |  |  |  | Generalised tonic clonic convulsion and prolonged unconsciousness. positive meningeal signs, disorientation, diminished sensation up to upper chest level,  *possibility of acute disseminated encephalomyelitis [ADEM]*, involvement of right 7 th , 9 th , 10 th and 12 th cranial nerves with quadriparesis . MRI findings suggested mild prominent cortical sulci. |
| [63] | March to June 2013 | 2013 | Nepal |  |  |  | Case 3 - transient peripheral neuropathy such as muscle weakness, tingling sensation, pins and needles, and numbness in his right arm and leg. |
| [64] | 2014 | 2016 | Dominican Republic - outbreak | Asian lineage |  | Enophthalmos 5/46 [14.6%] |  |
| [65] | 2014 | 2016 | USA - Chik patients identified through analysis of Veterans Health Administration medical records | Asian |  |  | Meningitis with a CSF profile consistent with a viral etiology -1/180, Altered mental status -17/180 [9.4%] |
| [66] | 2014 | 2016 | Guadeloupe |  |  |  | Central nervous system manifestations in 33/110, |
| [67] | 2014 | 2015 | USA - travellers with CHIKV returning to Rhode Island from Chik affected areas in the Caribbean and South America |  |  |  | 10/54 [19%] altered mental status, 1/54 [2%] seizures, 1/54 [2%] acute flaccid paralysis |
| [68] | Mar-2014 | 2014 | Traveler from the Kingdom of Tonga to the USA |  |  |  | 1/1 encephalitis, 1/1, altered mental status |
| [69] | May-2014 | 2014 | Traveller - physician returning to the USA from Haiti as a medical volunteer |  | Hyperpigmentation reported |  |  |
| [70] | 2014 - 2015 | 2015 | French Polynesia - increase in cases with Guillain-Barre syndrome associated with CHIKV |  |  |  | 8/9 Guillain Barré Syndrome, 1/9 - facial diplegia with sensory disorders of the face. |
| [71] | August 2014 to Jan 2015 | 2016 | Puerto Rico - retrospective review of hospital records of infants born to mothers with Chik-like symptoms |  |  |  | 3/7 neonates with congenital anomalies like hydrocephaly and brain abnormalities |
| [72] | August to October 2014 | 2016 | El Salvador, Colombia, Dominican Republic; pregnancy outcomes and consequences for infants born to Chik infected mothers | Asian | Hyperpigmentation - 4/169 [2.4%] in newborns born to CHIK suspected mothers |  | Meningoencephalitis - 12/169 [7.1%],in newborns born to CHIK suspected mothers |
| [73] | Jan - Nov 2014 | 2016 | French West Indies |  |  |  | Disorders of the central nervous system [CNS], including three who met criteria for encephalitis and two who had diffuse brain ischemia leading to brain death. 10/18 acute neurological disease. In addition, six patients [9%] had Guillain–Barre´ syndrome |
| [74] | Jan to Dec 2014 | 2017 | French West Indies - retrospective cases |  |  |  | Neurological symptoms - 130/687 |
| [75] | October 2014 to August 2015 | 2016 | Colombia |  |  |  | 57% Meningoencephalitis |
| [76] | October to November 2014 | 2015 | Venezuela - 3 adult patients with nasal skin necrosis seen at different medical centres | Asian | 3/3 adults - nasal skin necrosis - an unusual finding |  |  |
| [77] | September 2014 to Feb 2015 | 2015 | Colombia - case series of congenital ChikV |  |  |  | Meningoencephalitis - 2/8 newborns |
| [78] | Feb-2015 | 2016 | Colombia - differential diagnosis of DENV and CHIKV in pediatric cases |  |  |  | Neurological alterations 4/8 |
| [79] | Nov-2015 | 2016 | Brazil - vertical CHIKV transmission |  |  |  | 1/2-newborn: generalized seizures |
| [80] | November 2015 to Jan 2016 | 2016 | Brazil - case series of 14 infants with CHIKV infection |  | Infants >28 days under 2 years - 2/14 showed hyperpigmentation |  | seizures-1/14 |
| [81] | Before September 2015 | 2016 | Colombia - 2 cases of congenital and neonatal Chik |  |  |  | Encephalitis, generalized seizures, stupor |
| [82] | N/A | 2015 | India |  | 3/3 - Blistering hyperpigmented rash involving the face, limbs, trunk and tip of the nose |  | 1/3/tonic seizures, 2/3/multifocal clonic seizures |
| [83] | N/A | 2015 | Patient from St. Martin, presented to hospital in France |  | Periorbital hyperpigmentation | 1/1/blurred vision, 1/1/stromal keratouveitis | 1/1/neurological complications |
| [84] | N/A | 2014 | India |  |  |  | Paralysis -1/1 asymmetrical spastic quadriplegia, *1/1/acute demyelinating encephalomyelitis[ADEM]*, 1/1/sensory loss in lower extremities, trunk up to upper chest, patchy areas over both upper limbs as well |
| [85] | N/A | 2014 | India |  | Neonate born to CHIK infected mother showed hyperpigmentation on nose |  |  |
| [86] | N/A | 2014 | India - Neuro Chik study |  |  |  | Paralysis, specify: 2/5 Quadriparesis, 4/5 encephalitis/neuroCHIKV |
| [87] | N/A | 2013 | India |  |  | 1/1/Fuchs heterochromic iridocyclitis [FHI], 1/1/blurred vision, 1/1/pigmented KPs [keratic precipitates], 1/1/moth-eaten appearance of the iris |  |
| [88] | N/A | 2012 | India |  | Hyperpigmentation on nose and the upper lip, irregular, flagellate or “whip-lash” pattern |  |  |
| [89] | N/A | 2012 | India |  | 2/2 Diffuse hyperpigmentation |  |  |
| [90] | N/A | 2012 | India |  | 1/2 infants with congenital Chik - brownish- black pigmented macular lesions on nose, paranasal and perioral areas |  |  |
| [91] | N/A | 2010 | India |  | 1 year old child with blackish discolouration of his toes |  |  |
| [92] | N/A | 2008 | India |  |  |  | 2/2 encephalomyeloradiculitis, 2/2 viral encephalitis |
| [93] | N/A | 2008 | India - neonates with Chik infection |  | 1/4 neonates with hyperpigmentation on face, nose and also on abdomen, extremities and knuckles |  |  |
| [94] | N/A | 2006 | India | West African |  |  | 1%/876 meningoencephalitis |
| [95] | N/A | 2008 | India |  |  |  | *1/1 Acute Disseminated Encephalomyelitis [ADEM]*, Six days after subsiding of fever, the patient developed rapidly progressive quadriplegia and slurring of speech. The weakness progressed over 2 days and patient became bedridden. There was no alteration in sensorium or seizures. Examination on the second day of illness revealed, asymmetrical UMN quadriplegia with sustained ankle and patellar clonus. His speech was slurred and facial reflexes were exaggerated. He also had dystonic posturing of right upper limb |
| [96] | N/A | 2012 | India |  |  | 1/1 - bilateral Chikungunya neuroretinitis. Left eye showed 2+ cells in the anterior chamber. |  |
| [97] | N/A | 2011 | India - neonate with Chik born to mother infected with Chik prior to giving birth |  | Diffuse hyperpigmentation in baby |  |  |
| [98] | N/A | 2012 | India - neurodevelopmental outcomes in neonates with vertically transmitted ChikV |  | 2/2 infants with hyperpigmentation on nose, face, body |  | 2/2 encephalopathy. On admission one baby had features of encephalopathy, Case 1- developed spastic diplegia. He also has a seizure disorder |
| [99] | N/A | 2007 | India |  |  |  | 1/1 diagnosis of hypokalemic motor paralysis a week after infection. Could not test for GBS. |
| [100] | N/A | 2010 | India |  |  |  | 1/1 - Chikungunya myeloradiculopathy: A rare complication, 1/1 CNS examination revealed loss of abdominal reflexes, decreased power [3/5] in upper and lower limbs, |
| [101] | N/A | 2014 | India |  |  |  | Flaccid quadriparesis, *acute disseminated encephalomyelitis [ADEM]*, generalized tonic clonic seizure, loss of all sensory modalities below the C5 dermatome |
| [102] | N/A | 2013 | India |  |  |  | 1/1, generalized tonic–clonic seizures, severe myoclonus [grade IV] aggravated by touch, sound, or change in position |
| [103] | N/A | 2011 | India - neonatal Chik case |  | Baby developed hyperpigmentation on face followed by trunk and limbs |  | 1/2 -focal seizures Involving the left lower limb |
| [104] | N/A | 2011 | Reunion - materno-fetal Chik infection associated with Bernard-Soulier Syndrome |  |  |  | 1/1/convulsions |
| [105] | N/A | 2010 | Singapore - case report of an infant with Chik infection mimicking atypical Kawasaki disease |  |  |  | 1/1 - seizure |
| [106] | N/A | 2009 | India |  |  | 1/1 diminution of vision in both eyes; bilateral neuroretinitis with peripapillary cotton wool spots |  |
| [107] | N/A | 2008 | India |  |  | 1/1/A diagnosis of neuroretinitis was made in the right eye while left eye showed features of retinitis |  |
| [108] | N/A | 2007 | Reunion - 3 cases of GBS associated with Chik |  |  |  | Guillain Barré Syndrome manifestations: facial diplegia followed within 48 hours by tingling sensations and weakness in both hands and feet. 1/3; tetraparesis, predominant in the upper limbs, on admission 1/3; facial peripheral palsy. 1/3; acute weakness in both legs and paresthesia in four limbs on admission 1/3; symmetric paraparesis, proprioceptive ataxia more pronounced in the lower limbs, areflexia, and facial diplegia. 1/3 |
| [109] | N/A | 2007 | India - case series |  | Facial melanosis involving malar area in 30 cases, 3 of these patients also had pigmentation on other areas of the face |  |  |
| [110] | N/A | 2015 | India - Chik neonate case with mother testing negative for CHIKV |  | 1/1 peri-oral hyperpigmentation starting over the face and progressing to rest of the body |  | Microcephaly 1/1, seizures 1/1; severe neurodevelopmental delay 1/1 |
| [111] | N/A | 2016 | USA - Chik case study; patient returned from a Chik affected area. |  |  | 1/1 blurry vision and floaters in the right eye, unilateral intermediate uveitis |  |
| [112] | N/A | 2016 | Colombia - case with CHIKV and DENV3 co-infection |  |  |  | 1/1/altered state of consciousness |
| [113] | N/A | 2016 | India - Chik in neonate twins and their mother |  | Neonate twins born with CHIKV infection - hyperpigmentation in midfacial region |  | 2/3 encephalopathy, 2/3-seizures |
| [114] | N/A | 2016 | Brazil - case of DENV and CHIKV co-infection |  |  |  | 1/1-neurological examination showed right hemiparesis and ipsilateral sensory disorders, with Babinski sign on the right foot |
| [115] | N/A | 2016 | India - case report; unusual myelitis with myositis - Chik associated |  |  |  | Unusual acute transverse myelitis with myositis during convalescence 1/1 |
| [116] | N/A | 2015 | USA - case report of patient returning to the USA after travel to a Chik affected area |  |  | 1/1 uveitis and exudative retinal detachment |  |
| [117] | N/A | 2015 | Traveller returning to UK from West Indies |  |  | 1/1 acute optic neuropathy | 1/1 - an episode of right lower motor neuron facial palsy |
| [118] | N/A | 2016 | India - case report - 12 day old neonate with CHIKV with infected mother a week before delivery and continuing for 3 days postpartum |  | Neonate - progressive pigmented patches over the nose, upper lip, trunk, and lower extremities |  |  |
| [119] | N/A | 2016 | India - case report |  | Adult- Blotchy hyperpigmented macules were noted over the nose, periorbital area, cheeks and ears. |  |  |
| [120] | N/A | 2016 | Brazil - case report of ChikV associated myositis |  |  |  | 1/1 - mental confusion and aggressiveness. First reported case of encephalitis and myositis |
| [121] | N/A | 2015 | India - case report of detection of CHIKV from an encephalitis case | ECSA |  |  | 1/1 encephalitis, convulsions-1/1; altered sensorium-1/1 |

**References:**

1. Carey DE, Myers RM, Deranitz CM, Jadhav M, Reuben R. The 1964 chikungunya epidemic at vellore, South India, including observations on concurrent dengue. Trans R Soc Trop Med Hyg 1969;63(4):434-445.

2. Thiruvengadam KV, Kalyanasundaram V, Rajgopal J. Clinical and pathological studies on chikungunya fever in Madras city. Indian J Med Res 1965;53(8):729-744.

3. Mazaud R, Salaun JJ, Montabone H, Goube P, Bazillio R. Acute neurologic and sensorial disorders in dengue and Chikungunya fever. Bull Soc Pathol Exot Filiales 1971 Jan-Feb;64 (1):22-30.

4. Gu HX, Artsob H, Lin YZ, Wang DM, Zhao BY, Long QZ. Arboviruses as aetiological agents of encephalitis in the People's Republic of China. Trans R Soc Trop Med Hyg 1992;86 (2):198-201.

5. Economopoulou A, Dominguez M, Helynck B, Sissoko D, Wichmann O, Quenel P, et al. Atypical Chikungunya virus infections: clinical manifestations, mortality and risk factors for severe disease during the 2005-2006 outbreak on Reunion. Epidemiol Infect 2009;137 (4):534-541.

6. Dominguez M, Economopoulou A, Sissoko D, Boisson V, Gauzere BA, Pierre V, et al. Atypical forms of chikungunya in epidemic period, Reunion Island, France, 2005-2006. (Special issue - What did we learn from the chikungunya outbreak in the Indian Ocean in 2005-2006?) [French]. Bulletin Epidemiologique Hebdomadaire 2008 (38/40):349-352.

7. Ernould S, Walters H, Alessandri JL, Llanas B, Jaffar MC, Robin S, et al. Chikungunya in paediatrics: epidemic of 2005-2006 in Saint-Denis, Reunion Island. Archives de Pediatrie 2008;15 (3):253-262.

8. Talarmin F, Staiowsky F, Schoenlaub P, Risbourg A, Nicolas X, Zagnoli A, et al. Skin and mucosal manifestations of chikungunya virus infection in adults in Reunion Island. Med Trop 2007;67(2):167-173.

9. Renault P, Solet JL, Sissoko D, Balleydier E, Larrieu S, Filleul L, et al. A major epidemic of chikungunya virus infection on Reunion Island, France, 2005-2006. Am J Trop Med Hyg 2007;77 (4):727-731.

10. Gerardin P, Couderc T, Bintner M, Tournebize P, Renouil M, Lemant J, et al. Chikungunya virus-associated encephalitis: A cohort study on La Reunion Island, 2005-2009. Neurology 2016 Jan 5;86 (1):94-102.

11. Gerardin P, Barau G, Michault A, Bintner M, Randrianaivo H, Choker G, et al. Multidisciplinary prospective study of mother-to-child chikungunya virus infections on the Island of La Reunion. PLoS Medicine 2008;5 (3):e60.

12. Oliver M, Grandadam M, Marimoutou C, Rogier C, BotelhoNevers E, Tolou H, et al. Persisting mixed cryoglobulinemia in Chikungunya infection. PLoS Neglected Tropical Diseases 2009;3 (2):e374.

13. Lemant J, Boisson V, Winer A, Thibault L, Andre H, Tixier F, et al. Serious acute Chikungunya virus infection requiring intensive care during the Reunion Island outbreak in 2005-2006. Crit Care Med 2008;36 (9):2536-2541. 35

14. Paquet C, Quatresous I, Solet JL, Sissoko D, Renault P, Pierre V, et al. Chikungunya outbreak in Reunion: epidemiology and surveillance, 2005 to early January 2006. Euro Surveill 2006 Feb 2;11(2):E060202.3.

15. Iborra C, Fite S, Thia-Thiong-Fat C, Hope-Rapp E, Assouere M-. Skin pigmentation disorders during the Chikungunya infection in Reunion. Nouvelles Dermatologiques 2007;26(4 SPEC. ISS.):20-21.

16. Robillard PY, Boumahni B, Gerardin P, Michault A, Fourmaintraux A, Schuffenecker I, et al. Vertical maternal-fetal transmission of chikungunya virus: ten cases on Reunion among 84 pregnant women. Presse Medicale 2006;355 (Cahier 1):785-788.

17. Paquet C, Quatresous I, Solet JL, Sissoko D, Renault P, Pierre V, et al. Epidemiology of chikungunya virus infection on the Island of Reunion: situation on 8 January 2006. (Infection par le virus Chikungunya a l'Ile de la Reunion) [French]. Bulletin Epidemiologique Hebdomadaire 2006 (hors serie):2-3.

18. Bessaud M, Peyrefitte CN, Pastorino BAM, Tock F, Merle O, Colpart JJ, et al. Chikungunya virus strains, Reunion Island outbreak. Emerging Infectious Diseases 2006;12 (10):1604-1606.

19. Tournebize P, Charlin C, Lagrange M. Neurological manifestations in chikungunya: about 23 cases collected in Reunion Island. Rev Neurol 2009;165 (1):48-51.

20. Ramful D, Carbonnier M, Pasquet M, Bouhmani B, Ghazouani J, Noormahomed T, et al. Mother-to-child transmission of chikungunya virus infection. Pediatr Infect Dis J 2007;26 (9):811-815.

21. Quatresous I, Investigation Group. E-alert 27 January: Chikungunya outbreak in Reunion, a French overseas department. Euro Surveill 2006 Feb 2;11(2):E060202.1.

22. Gaüzère B-, Bohrer M, Drouet D, Gasque P, Jaffar-Bandjee M-, Filleul L, et al. Chikungunya virus infection in Reunion Island in 2005-2006: Severe emerging adult forms in the intensive care unit. Reanimation 2011;20 (3):211-222.

23. Wadia RS. A neurotropic virus [chikungunya] and a neuropathic aminoacid [homocysteine]. Annals of Indian Academy of Neurology 2007;10 (4):198-213.

24. Doke PP, Dakhure DS, Patil AV. A clinico-epidemiological study of chikungunya outbreak in Maharashtra state, India. Indian J Public Health 2011;55(4):313-316.

25. Suryawanshi SD, Dube AH, Khadse RK, Jalgaonkar SV, Sathe PS, Zawar SD, et al. Clinical profile of chikungunya fever in patients in a tertiary care centre in Maharashtra, India. Indian J Med Res 2009;129 (4):438-441.

26. Lebrun G, Chadda K, Reboux AH, Martinet O, Gauzere BA. Guillain-Barre syndrome after chikungunya infection. Emerging Infectious Diseases 2009;15 (3):495-496.

27. Tandale BV, Sathe PS, Arankalle VA, Wadia RS, Rahul Kulkarni, Shah SV, et al. Systemic involvements and fatalities during Chikungunya epidemic in India, 2006. Journal of Clinical Virology 2009;46 (2):145-149.

28. Staikowsky F, Roux Kl, Schuffenecker I, Laurent P, Grivard P, Develay A, et al. Retrospective survey of Chikungunya disease in Reunion Island hospital staff. Epidemiol Infect 2008;136 (2):196-206.

29. Rampal, Sharda M, Meena H. Neurological complications in Chikungunya fever. J Assoc Physicians India 2007 Nov;55:765-769.

30. Bomin Al, Hebert JC, Marty P, Delaunay P. Confirmed chikungunya in children in Mayotte. Description of 50 patients hospitalized from February to June 2006. Revue Medecine Tropicale 2008;68 (5):491-495.

31. Pellot AS, Alessandri JL, Robin S, Samperiz S, Attali T, Brayer C, et al. Severe forms of chikungunya virus infection in a pediatric intensive care unit on Reunion Island. Med Trop (Mars) 2012 Mar;72 Spec No:88-93.

32. Robin S, Ramful D, Seach Fl, JaffarBandjee MC, Rigou G, Alessandri JL. Neurologic manifestations of pediatric Chikungunya infection. J Child Neurol 2008;23 (9):1028-1035.

33. Lewthwaite P, Ravi Vasanthapuram, Osborne JC, Ashia Begum, Plank JLM, Shankar MV, et al. Chikungunya virus and central nervous system infections in children, India. Emerging Infectious Diseases 2009;15 (2):329-331.

34. Prashant S, Kumar A, Mohammed Basheeruddin D, Chowdhary T, Madhu B. Cutaneous manifestations in patients suspected of chikungunya disease. Indian J Dermatol 2009;54 (2):128-131.

35. Kashyap RS, Morey SH, Chandak NH, Purohit HJ, Taori GM, Daginawala HF. Detection of viral antigen, IgM and IgG antibodies in cerebrospinal fluid of Chikungunya patients with neurological complications. Cerebrospinal Fluid Res 2010 Aug 13;7:12-8454-7-12.

36. Chandak NH, Kashyap RS, Dinesh Kabra, Poornima Karandikar, Saha SS, Morey SH, et al. Neurological complications of Chikungunya virus infection. Neurol India 2009;57 (2):177-180.

37. Inamadar AC, Aparna Palit, Sampagavi VV, Raghunath S, Deshmukh NS. Cutaneous manifestations of chikungunya fever: observations made during a recent outbreak in south India. Int J Dermatol 2008;47 (2):154-159.

38. Prajna Lalitha, Sivakumar Rathinam, Krishnadas Banushree, Shanmugam Maheshkumar, Rajendran Vijayakumar, Padmakar S. Ocular involvement associated with an epidemic outbreak of chikungunya virus infection. Am J Ophthalmol 2007;144 (4):552-556.

39. Apoorva Mittal, Saurabh Mittal, Bharati MJ, Rengappa Ramakrishnan, Sankarlingam Saravanan, Sathe PS. Optic neuritis associated with Chikungunya virus infection in South India. Arch Ophthalmol 2007;125 (10):1381-1386.

40. Nair PMC. Chikungunya in neonates. Indian Pediatr 2008;45 (7):605.

41. Elenjickal MG, Sushamabai S. Outbreak of Chikungunya disease in Kerala in 2007. Indian Pediatr 2009;46 (5):440-441.

42. Casolari S, Briganti E, Zanotti M, Zauli T, Nicoletti L, Magurano F, et al. A fatal case of encephalitis associated with Chikungunya virus infection. Scand J Infect Dis 2008;40 (11/12):995-996.

43. Senanayake MP, Senanayake SM, Vidanage KK, Gunasena S, Lamabadusuriya SP. Vertical transmission in chikungunya infection. Ceylon Med J 2009 Jun;54 (2):47-50.

44. Rose N, Anoop TM, John AP, Jabbar PK, George KC. Acute optic neuritis following infection with chikungunya virus in southern rural India. Int J Infect Dis 2011 Feb;15 (2):e147-50.

45. Paul BJ, Pannarkady G, Moni SP, Thachil EJ. Clinical profile and long-term sequelae of Chikungunya fever. Indian Journal of Rheumatology 2011;6 (1 SUPPL.):12-19.

46. Valamparampil JJ, Chirakkarot S, Letha S, Jayakumar C, Gopinathan KM. Clinical profile of Chikungunya in infants. Indian J Pediatr 2009 Feb;76 (2):151-155.

47. Kee ACL, Yang S, Tambyah P. Atypical Chikungunya virus infections in immunocompromised patients. Emerging Infectious Diseases 2010;16 (6):1038-1040.

48. Manimunda SP, Paluru Vijayachari, Raghuraj Uppoor, Sugunan AP, Singh SS, Rai SK, et al. Clinical progression of chikungunya fever during acute and chronic arthritic stages and the changes in joint morphology as revealed by imaging. Trans R Soc Trop Med Hyg 2010;104 (6):392-399.

49. Bhat RM, Yashaswi Rai, Amitha Ramesh, Nandakishore B, Sukumar D, Jacintha Martis, et al. Mucocutaneous manifestations of Chikungunya fever: a study from an epidemic in coastal Karnataka. Indian J Dermatol 2011;56 (3):290-294.

50. Prasanna Chandra, Biswajit Das, Sowmya MK, Sumeru Samanta, Thimmaraju KV. Epidemiologal study on Chikungunya outbreak in Mangalore district, Karnataka. International Journal of Pharma and Bio Sciences 2011;2 (4):B-342-B-347.

51. Seetharam KA, Sridevi K, Vidyasagar P. Cutaneous manifestations of chikungunya fever. Indian Pediatr 2012;49 (1):51-53.

52. Laoprasopwattana K, Kaewjungwad L, Jarumanokul R, Geater A. Differential diagnosis of chikungunya, dengue viral infection and other acute febrile illnesses in children. Pediatr Infect Dis J 2012;31(5):459-463.

53. Chusri S, Siripaitoon P, Hirunpat S, Silpapojakul K. Case reports of neuro-chikungunya in Southern Thailand. Am J Trop Med Hyg 2011;85 (2):386-389.

54. Chusri S, Siripaitoon P, Silpapojakul K. Chikungunya outbreak in Southern Thailand, December 2008 to October 2009. J Infect Dis Antimicrobial Agents 2011;28 (1):25-34.

55. Babu K, Kini R, Philips M, Subbakrishna DK. Clinical profile of isolated viral anterior uveitis in a South Indian patient population. Ocul Immunol Inflamm 2014 Oct;22 (5):356-359.

56. Jaheersha Pakran, Mamatha George, Najeeba Riyaz, Riyaz Arakkal, Sandhya George, Uma Rajan, et al. Purpuric macules with vesiculobullous lesions: a novel manifestation of Chikungunya. Int J Dermatol 2011;50 (1):61-69.

57. Najeeba Riyaz, Riyaz A, Rahima, Abdul Latheef EN, Anitha PM, Aravindan KP, et al. Cutaneous manifestations of chikungunya during a recent epidemic in Calicut, north Kerala, south India. Indian Journal of Dermatology, Venereology & Leprology 2010;76 (6):671-676.

58. Schwartz KL, Giga A, Boggild AK. Chikungunya fever in Canada: fever and polyarthritis in a returned traveller. CMAJ 2014 Jul 8;186 (10):772-774.

59. Gunasekaran P, Saravanamurali K, Mohana S, Kavitarunagiri, Senthilkumar V, Kaveri K. Dermatological atypical manifestations of Chikungunya infection in Tamilnadu-2010. International Journal of Pharma and Bio Sciences 2012;3 (4):B-374-B-379.

60. Chakravarti A, Malik S, Tiwari S, Ashraf A. A study of Chikungunya outbreak in Delhi. J Commun Dis 2011 Dec;43 (4):259-263.

61. Vishwanath S, Badami K, Sriprakash KS, Sujatha BL, Shashidhar SD, Shilpa YD. Post-fever retinitis: a single center experience from south India. Int Ophthalmol 2014 Aug;34 (4):851-857.

62. Taraphdar D, Roy BK, Chatterjee S. Chikungunya virus infection amongst the acute encephalitis syndrome cases in West Bengal, India. Indian Journal of Medical Microbiology 2015;33 (5(Suppl.):153-156.

63. Pun SB, Anup Bastola, Rajesh Shah. First report of Chikungunya virus infection in Nepal. Journal of Infection in Developing Countries 2014;8 (6):790-792.

64. Langsjoen RM, Rubinstein RJ, Kautz TF, Auguste AJ, Erasmus JH, Kiaty-Figueroa L, et al. Molecular Virologic and Clinical Characteristics of a Chikungunya Fever Outbreak in La Romana, Dominican Republic, 2014. PLoS Negl Trop Dis 2016 Dec 28;10 (12):e0005189.

65. Perti T, Lucero-Obusan CA, Schirmer PL, Winters MA, Holodniy M. Chikungunya Fever Cases Identified in the Veterans Health Administration System, 2014. PLoS Negl Trop Dis 2016 May 4;10 (5):e0004630.

66. Rolle A, Schepers K, Cassadou S, Curlier E, Madeux B, Hermann-Storck C, et al. Severe Sepsis and Septic Shock Associated with Chikungunya Virus Infection, Guadeloupe, 2014. Emerg Infect Dis 2016 May;22 (5):891-894.

67. Gosciminski M, Bandy U, Brady DS. Travel Associated Cases of Chikungunya Fever, Rhode Island, 2014. R I Med J [2013] 2015 Sep 1;98 (9):47-49.

68. Nelson J, Waggoner JJ, Sahoo MK, Grant PM, Pinsky BA. Encephalitis caused by Chikungunya virus in a traveler from the Kingdom of Tonga. J Clin Microbiol 2014;52 (9):3459-3461.

69. Anderson KB, Pureza V, Walker PF. Chikungunya: acute fever, rash and debilitating arthralgias in a returning traveler from Haiti. Journal of Travel Medicine 2014;21(6):418-420.

70. Oehler E., Fournier E., Leparc-Goffart I., Larre P., Cubizolle S., Sookhareea C., et al. Increase in cases of guillain-barre syndrome during a chikungunya outbreak, French Polynesia, 2014 to 2015,2015; Euro Surveill. 2015;20 (48):30079.

71. Rodriguez-Nieves M, Garcia-Garcia I, Garcia-Fragoso L. Perinatally Acquired Chikungunya Infection: The Puerto Rico Experience. Pediatr Infect Dis J 2016 Oct;35 (10):1163.

72. Torres JR, Falleiros-Arlant LH, Duenas L, Pleitez-Navarrete J, Salgado DM, Castillo JB. Congenital and perinatal complications of chikungunya fever: a Latin American experience. Int J Infect Dis 2016 Oct;51:85-88.

73. Crosby L, Perreau C, Madeux B, Cossic J, Armand C, Herrmann-Storke C, et al. Severe manifestations of chikungunya virus in critically ill patients during the 2013-2014 Caribbean outbreak. Int J Infect Dis 2016 Jul;48:78-80.

74. Godaert L, Najioullah F, Bousquet L, Malmontet T, Fournet B, Cesaire R, et al. Do Two Screening Tools for Chikungunya Virus Infection that were Developed among Younger Population Work Equally as Well in Patients Aged over 65 Years? PLoS Negl Trop Dis 2017 Jan 5;11(1):e0005256.

75. Raad J.J., Rosero A.S., Martinez J.V., Parody A., Raad R.J., Tovar D.C., et al. Immunological response of a population from the caribbean region of Colombia infected with the chikungunya virus. 2016; Rev.Colomb.Reumatol 2016; 23 (2):85-91.

76. Torres JR, Cordova LG, Saravia V, Arvelaez J, Castro JS. Nasal Skin Necrosis: An Unexpected New Finding in Severe Chikungunya Fever. Clin Infect Dis 2016 Jan 1;62(1):78-81.

77. Villamil-Gomez W, Alba-Silvera L, Menco-Ramos A, Gonzalez-Vergara A, Molinares-Palacios T, Barrios-Corrales M, et al. Congenital Chikungunya Virus Infection in Sincelejo, Colombia: A Case Series. J Trop Pediatr 2015 Oct;61(5):386-392.

78. Calvo EP, Coronel-Ruiz C, Velazco S, Velandia-Romero M, Castellanos JE. Diagnóstico diferencial de dengue y chikungunya en pacientes pediátricos^ies; Dengue and Chikungunya differential diagnosis in pediatric patients^ien. Biomédica (Bogotá) 2016 08;36:35-43.

79. Bandeira AC, Campos GS, Sardi SI, Rocha VF, Rocha GC. Neonatal encephalitis due to Chikungunya vertical transmission: First report in Brazil. IDCases 2016 Jul 25;5:57-59.

80. do Carmo Menezes BD, de ON, de MB, Cavalcanti LA, de BS, de Abreu SGAA, et al. Chikungunya infection in infants. Revista Brasileira de Saude Materno Infantil 2016;16:S63-S71.

81. Alvarado-Socarras JL, Ocampo-Gonzalez M, Vargas-Soler JA, Rodriguez-Morales AJ, Franco-Paredes C. Congenital and Neonatal Chikungunya in Colombia. J Pediatric Infect Dis Soc 2016 Sep;5 (3):e17-20.

82. Gupta D, Bose A, Rose W. Acquired Neonatal Chikungunya Encephalopathy. Indian J Pediatr 2015 Apr 14.

83. Hayek S, Rousseau A, Bouthry E, Prat CM, Labetoulle M. Chikungunya Virus Infection and Bilateral Stromal Keratouveitis. JAMA Ophthalmol 2015 Apr 9.

84. Maity P, Roy P, Basu A, Das B, Ghosh US. A case of ADEM following Chikungunya fever. J Assoc Physicians India 2014 May;62 (5):441-442.

85. Kumar N, Gupta V, Thomas N. Brownie-nose: hyperpigmentation in neonatal chikungunya. Indian Pediatr 2014 May;51 (5):419.

86. Kashyap RS, Morey S, Bhullar S, Baheti N, Chandak N, Purohit H, et al. Determination of Toll-like receptor-induced cytokine profiles in the blood and cerebrospinal fluid of Chikungunya patients. Neuroimmunomodulation 2014;21(6):338-346.

87. Babu K, Adiga M, Govekar SR, Kumar BR, Murthy KR. Associations of Fuchs heterochromic iridocyclitis in a South Indian patient population. J Ophthalmic Inflamm Infect 2013 Jan 15;3 (1):14-5760-3-14.

88. Kandhari R, Khunger N, Singh A. Flagellate pigmentation and exacerbation of melasma following chikungunya fever: a less frequently reported finding. Indian J Dermatol Venereol Leprol 2012 Nov-Dec;78 (6):774.

89. Gopakumar H, Ramachandran S. Congenital chikungunya. J Clin Neonatol 2012 Jul;1 (3):155-156.

90. Khandelwal K, Aara N, Ghiya BC, Bumb RA, Satoskar AR. Centro-facial pigmentation in asymptomatic congenital chikungunya viral infection. J Paediatr Child Health 2012 Jun;48 (6):542-543.

91. Adhisivam B. Chikungunya rash. Indian Pediatr 2010 Apr;47(4):348.

92. Ganesan K, Diwan A, Shankar SK, Desai SB, Sainani GS, Katrak SM. Chikungunya encephalomyeloradiculitis: report of 2 cases with neuroimaging and 1 case with autopsy findings. AJNR Am J Neuroradiol 2008 Oct;29 (9):1636-1637.

93. Rao G, Khan YZ, Chitnis DS. Chikungunya infection in neonates. Indian Pediatr 2008 Mar;45(3):240-242.

94. Mohan A. Chikungunya fever: clinical manifestations & management. Indian J Med Res 2006 Nov;124 (5):471-474.

95. Mohamed Musthafa AK, Abdurahiman P, Jose J. A case of ADEM following Chikungunya fever. Journal of Association of Physicians of India 2008;56 (JUNE):473.

96. Nair AG, Biswas J, Bhende MP. A case of bilateral Chikungunya neuroretinitis. Journal of Ophthalmic Inflammation and Infection 2012;2 (1):39-40.

97. Shrivastava A, Waqar Beg M, Gujrati C, Gopalan N, Rao PVL. Management of a vertically transmitted neonatal Chikungunya thrombocytopenia. Indian J Pediatr 2011;78 (8):1008-1009.

98. Shenoy S, Pradeep GCM. Neurodevelopmental outcome of neonates with vertically transmitted chikungunya fever with encephalopathy. Indian Pediatr 2012;49 (3):238-239.

99. Rampal, Sharda M, Meena H. Hypokalemic paralysis following chikungunya fever [2]. Journal of Association of Physicians of India 2007;55 (AUG.):598.

100. Rahul MK, Krishnamoorthy. Chikungunya myeloradiculopathy: A rare complication. Journal of Global Infectious Diseases 2012;4[4]:207-208.

101. Suman Das, Nirmalya Sarkar, Jayitri Majumder, Kaushani Chatterjee, Bholanath A. Acute disseminated encephalomyelitis in a child with chikungunya virus infection. Journal of Pediatric Infectious Diseases 2014;9[1]:37-41.

102. Kalita J, Kumar P, Misra UK. Stimulus-sensitive myoclonus and cerebellar ataxia following chikungunya meningoencephalitis. Infection 2013;41(3):727-729.

103. Shetty PK. Neonatal chikungunya - a case report. Pediatric OnCall 2011;8 (12):80.

104. Boumahni B, Kaplan C, Clabe A, Randrianaivo H, Lanza F. Maternal-fetal chikungunya infection associated with Bernard-Soulier syndrome. (Neonatologie.) (French). Archives de Pediatrie 2011;18 (3):272-275.

105. Lee YungSeng, Quek SweeChye, Koay SiewChuan, Tang W. Chikungunya mimicking atypical Kawasaki disease in an infant. Pediatr Infect Dis J 2010;29 (3):275-277.

106. Mahesh G, Giridhar A, Archis Shedbele, Ram Kumar, Saikumar SJ. A case of bilateral presumed chikungunya neuroretinitis. Indian J Ophthalmol 2009;57 (2):148-150.

107. Murthy KR, Nandita Venkataraman, Vidya Satish KB. Bilateral retinitis following chikungunya fever. Indian J Ophthalmol 2008;56 (4):329-331.

108. Wielanek AC, Monredon Jd, ElAmrani M, Roger JC, Serveaux JP. Guillain-Barre syndrome complicating a Chikungunya virus infection. Neurology 2007;69 (22):2105-2107.

109. Shivakumar V, Rajendra Okade, Rajkumar V, Rajashekar TS. Unusual facial melanosis in viral fever. Indian J Dermatol 2007;52(2):116-117.

110. Peter R, Krishnan L, Anandraj V, Kuruvila S. Chikungunya in a newborn. Journal of Clinical Neonatology 2015 01 Apr 2015;4 (2):145-146.

111. Lin J, Chen RW, Hazan A, Weiss M. Chikungunya Virus Infection Manifesting as Intermediate Uveitis. Ocul Immunol Inflamm 2016 Dec 23:1-3.

112. Rosso F, Pacheco R, Rodriguez S, Bautista D. Co-infection by Chikungunya virus (CHIK-V) and dengue virus (DEN-V) during a recent outbreak in Cali, Colombia: Report of a fatal case. Rev Chilena Infectol 2016 Aug;33 (4):464-467.

113. Karthiga V, Kommu PP, Krishnan L. Perinatal chikungunya in twins. J Pediatr Neurosci 2016 Jul-Sep;11 (3):223-224.

114. Brooks JB, Ruiz CA, Fragoso YD. Acute illness with neurological findings caused by coinfection of dengue and chikungunya viruses in a Brazilian patient. J Infect Public Health 2016 Sep 5.

115. Choudhary N, Makhija P, Puri V, Khwaja GA, Duggal A. An Unusual Case of Myelitis with Myositis. J Clin Diagn Res 2016 May;10 (5):OD19-20.

116. Scripsema NK, Sharifi E, Samson CM, Kedhar S, Rosen RB. Chikungunya-Associated Uveitis and Exudative Retinal Detachment: a Case Report. Retin Cases Brief Rep 2015 Fall;9 (4):352-356.

117. Mohite AA, Agius-Fernandez A. Chikungunya fever presenting with acute optic neuropathy. BMJ Case Rep 2015 Jul 28;2015:10.1136/bcr-2015-210081.

118. Vasani R, Kanhere S, Chaudhari K, Phadke V, Mukherjee P, Gupta S, et al. Congenital Chikungunya--A Cause of Neonatal Hyperpigmentation. Pediatr Dermatol 2016 Mar-Apr;33 (2):209-212.

119. Mishra AK, George AA, Sadhasiv M, Sathyendra S. Sign in Chikungunya Fever. Journal of Association of Physicians of India 2016;64:74.

120. Martins HA, Bernardino SN, Santos CC, Ribas VR. Chikungunya and myositis: A case report in Brazil. Journal of Clinical and Diagnostic Research 2016;10 (12):OD05-OD06.

121. Shaikh N, Raut C, Sinha D, Manjunath M. Detection of Chikungunya virus from a case of encephalitis, Bangalore, Karnataka State; 26068360. Indian Journal of Medical Microbiology 2015;33 (3):454-455.
