# Supplementary material for: A scoping review of published literature on chikungunya virus
Source: PLoS One. 2018 Nov 29;13(11):e0207554. doi: 10.1371/journal.pone.0207554 (PMC6264817; doi:10.1371/journal.pone.0207554)
Supplement: S5 Table — *Pathogen not specified. (DOCX) [file pone.0207554.s008.docx]

| Co-infection  **S5 Table: Reported co-infections with CHIKV** | Publication period | Country | Refid |
| --- | --- | --- | --- |
| CHIKV+DENV | 1965; 1967; 1969; 2008; 2009; 2011; 2012; 2012; 2012; 2013; 2013; 2014; 2014; 2014; 2015; 2015; 2015; 2016; 2016 | India | [1]; [2]; [3]; [4]; [5]; [6]; [7]; [8]; [9]; [10]; [11]; [12]; [13]; [14]; [15]; [16]; [17]; [18]; [19] |
| CHIKV+DENV | 1966; 1969; 2012 | Thailand | [20]; [21]; [22] |
| CHIKV+DENV | 1974; 2014 | Burma / Myanmar | [23]; [24] |
| CHIKV+DENV | 1994 | Vietnam | [25] |
| CHIKV+DENV | 2007; 2016 | Malaysia | [26]; [27] |
| CHIKV+DENV | 2008; 2009 | Sri Lanka | [28]; [29] |
| CHIKV+DENV | 2008 | Madagascar | [30] |
| CHIKV+DENV | 2008 | Belgium | [31] |
| CHIKV+DENV | 2009; 2012; 2012 | Gabon | [32]; [33]; [34] |
| CHIKV+DENV | 2009 | Germany | [35] |
| CHIKV+DENV | 2009 | Maldives | [36] |
| CHIKV+DENV | 2010; 2016 | Taiwan | [37]; [38] |
| CHIKV+DENV | 2014 | Portugal | [39] |
| CHIKV+DENV | 2014; 2015 | France | [40]; [41] |
| CHIKV+DENV | 2015 | Panama | [42] |
| CHIKV+DENV | 2015; 2016; 2016 | Colombia | [43]; [44]; [45] |
| CHIKV+DENV | 2015; 2015; 2016 | USA | [46]; [47]; [48] |
| CHIKV+DENV | 2015 | Nepal | [49] |
| CHIKV+DENV | 2016 | Puerto Rico | [50] |
| CHIKV+DENV | 2016; 2016; 2016 | Brazil | [51]; [52]; [53]; |
| CHIKV+DENV | 2016 | Guatemala | [54] |
| CHIKV+DENV | 2016; 2016 | Spain | [55]; [56] |
| CHIKV+DENV | 2016 | Lao PDR | [57] |
| CHIKV+DENV | 2016 | Canada | [58] |
| CHIKV+DENV | 2016 | Mexico | [59] |
| CHIKV+DENV | 2016 | Nicaragua | [60] |
| Bacillary dysentery*; acute bronchitis*;pneumonia*; tonsillitis*; malaria; ancylostomiasis; septic lesions* and gonorrhoea | 1955 | Tanganyika territory | [61] |
| CHIKV + Sindbis virus; CHIKV + group B arboviruses - could be any of JE; WNV; DENV; Kyasanur forest disease virus | 1965 | India | [62] |
| CHIKV + Group B virus* | 1967 | Sri Lanka | [63] |
| CHIKV + O'nyong-nyong | 1970 | Kenya | [64] |
| CHIKV + O'nyong-nyong | 1970 | Uganda | [65] |
| CHIKV + Semliki Forest | 1973 | Kuwait | [66] |
| CHIKV with either pneumonia*; primary pulmonary tuberculosis; coliform urinary tract infection | 1974 | Nigeria | [67] |
| CHIKV + malaria | 2003 | Democratic Republic of Congo | [68] |
| CHIKV + malaria | 2004 | Democratic Republic of Congo | [69] |
| CHIKV + Escherichia coli septicemia | 2006 | France | [70] |
| CHIKV + Klebsiella pneumoniae | 2007 | IOI | [71] |
| CHIKV and flaviviruses* | 2007 | Cameroon | [72] |
| CHIKV + pneumonia*; or possible nosocomial septicemia due to Pseudomonas | 2008 | IOI | [73] |
| CHIKV + Klebsiella pneumoniae | 2008 | IOI | [74] |
| CHIKV + Klebsiella pneumoniae | 2008 | IOI | [75] |
| CHIKV + amoebiasis | 2008 | France | [76] |
| CHIKV + either DENV; HIV or malaria | 2009 | Spain | [77] |
| CHIKV + either Tahyna virus [TAHV]; WNV; untypeable bunyavirus* | 2010 | Guinea | [78] |
| CHIKV + Klebsiella pneumoniae; Escherichia coli; and Candida glabrata | 2010 | Singapore | [79] |
| CHIKV + malaria | 2010 | Spain | [80] |
| CHIKV +HIV | 2011 | Thailand | [81] |
| CHIKV+DENV+Malaria | 2012 | Gabon | [82] |
| TB | 2016 | USA | [83] |
| CHIKV + either HIV or DENV | 2012 | Tanzania | [84] |
| CHIKV + Japanese encephalitis virus [JEV] | 2013 | India | [85] |
| CHIKV + either DENV; malaria; HIV; Hepatitis B ; Hepatitis A; Hepatitis C; tuberculosis; typhoid or syphilis. | 2013 | Sierra Leone | [86] |
| CHIKV+eitherDENV; WNV; YFV; malaria or typhoid | 2013 | Nigeria | [87] |
| CHIKV + Malaria; CHIKV +DENV | 2014 | Tanzania | [88] |
| CHIKV + Malaria | 2015 | Benin | [89] |
| CHIKV + Malaria; CHIKV +DENV | 2015 | India | [90] |
| CHIKV + HIV | 2015 | Italy | [91] |
| CHIKV + either DENV or typhoid fever* | 2015 | Colombia | [92] |
| Triple co-infection CHIKV + DENV + ZIKV | 2016 | Colombia | [93] |
| CHIKV + Rift Valley fever virus | 2016 | Sudan | [94] |
| Triple co-infection CHIKV + DENV + ZIKV | 2016 | Nicaragua | [95] |
| CHIKV + either DENV or ZIKV | 2016 | Brazil | [96] |
| Triple co-infection CHIKV + DENV + ZIKV | 2016 | Colombia | [97] |
| CHIKV and Leptospirosis | 2016 | French Polynesia | [98] |
| CHKV and ZIKV | 2016 | Brazil | [99] |
| CHIKV +either DENV or Acinetobacter baumannii | 2016 | Colombia | [100] |
| DENV; CHIKV and ZIKV in combinations of 2 or 3 of these infections | 2016 | USA | [101] |
| CHIKV and HIV | 2016 | Argentina | [102] |
| CHIKV + Pseudomonas aeruginosa | 2016 | Senegal | [103] |
| CHIKV + Malaria or Zika | 2016 | Senegal | [104] |
| CHIKV + either ZIKV or DENV | 2016 | Colombia | [105] |
| CHIKV + Malaria | 2016 | Nigeria | [106] |
| CHIKV + ZIKV | 2016 | Ecuador | [107] |
| CHIKV + either DENV; Pseudomonas aeruginosa; Enterococcus faecalis or Morganella morganii | 2016 | Colombia | [108] |
| CHIKV + cellulitis* | 2017 | Brazil | [109] |

*Pathogen not specified

**References:**

1. Sarkar JK, Chatterjee SN, Chakravarti SK, Mitra AC. Chikungunya virus infection with haemorrhagic manifestations. Indian J Med Res 1965;53(10):921-925.

2. Myers RM, Carey DE. Concurrent isolation from patient of two arboviruses, Chikungunya and dengue type 2. Science 1967 Sep 15;157(3794):1307-1308.

3. Carey DE, Myers RM, Deranitz CM, Jadhav M, Reuben R. The 1964 chikungunya epidemic at vellore, South India, including observations on concurrent dengue. Trans R Soc Trop Med Hyg 1969;63(4):434-445.

4. Dash PK, Manmohan Parida, Santhosh SR, Parag Saxena, Ambuj Srivastava, Mamidi Neeraja, et al. Development and evaluation of a 1-step duplex reverse transcription polymerase chain reaction for differential diagnosis of chikungunya and dengue infection. Diagn Microbiol Infect Dis 2008;62(1):52-57.

5. Chahar HS, Preeti Bharaj, Lalit Dar, Randeep Guleria, Kabra SK, Shobha Broor. Co-infections with chikungunya virus and dengue virus in Delhi, India. Emerging Infectious Diseases 2009;15(7):1077-1080.

6. Usha Kalawat, Sharma KK, Reddy SG. Prevalence of dengue and chickungunya fever and their co-infection. Indian J Pathol Microbiol 2011;54(4):844-846.

7. Taraphdar D, Sarkar A, Mukhopadhyay BB, Chatterjee S. Short report: A comparative study of clinical features between monotypic and dual infection cases with chikungunya virus and dengue virus in West Bengal, India. Am J Trop Med Hyg 2012;86(4):720-723.

8. Kumar KJ, Manjunath VG, Shailashree M, Girish GN. Coinfection with dengue and chikungunya - a case report. J Indian Med Assoc 2012;110(10):749-752.

9. Singh P, Mittal V, Rizvi MMA, Chhabra M, Sharma P, Rawat DS, et al. The first dominant co-circulation of both dengue and chikungunya viruses during the post-monsoon period of 2010 in Delhi, India. Epidemiol Infect 2012;140(7):1337-1342.

10. Mohanty I, Dash M, Sahu S, Narasimham MV, Panda P, Padhi S. Seroprevalence of chikungunya in southern odisha. J Family Med Prim Care 2013 Jan;2(1):33-36.

11. Neeraja M, Lakshmi V, Dash PK, Parida MM, Rao PVL. The clinical, serological and molecular diagnosis of emerging dengue infection at a tertiary care institute in Southern, India. Journal of Clinical and Diagnostic Research 2013;7(3):457-461.

12. Afreen N, Deeba F, Khan WH, Haider SH, Kazim SN, Ishrat R, et al. Molecular characterization of dengue and chikungunya virus strains circulating in New Delhi, India. Microbiol Immunol 2014 Dec;58(12):688-696.

13. Dutta SK, Tithi Pal, Bibhuti Saha, Syamsundar Mandal, Anusri Tripathi. Copy number variation of Chikungunya ECSA virus with disease symptoms among Indian patients. J Med Virol 2014;86(8):1386-1392.

14. Suguna Dumpala, Nagaraj Kondagunta, Malhotra VM, Venna GP, Jothula KY. An outbreak investigation of suspected Chikungunya fever in Nalgonda District, Telangana state. Journal of Clinical and Scientific Research 2014;3(4):219-223.

15. Saswat T, Kumar A, Kumar S, Mamidi P, Muduli S, Debata NK, et al. High rates of co-infection of Dengue and Chikungunya virus in Odisha and Maharashtra, India during 2013. Infect Genet Evol 2015 Oct;35:134-141.

16. Shaikh N, Raut C, Manjunatha M. Co-infections with chikungunya and dengue viruses: A serological study in Karnataka State, India; 26068364. Indian Journal of Medical Microbiology 2015;33(3):459-460.

17. Dayakar S., Goud I.K., Pillai H., Remadevi V., Dharmaseelan S., Nair R.R., et al. Molecular Diagnosis of Chikungunya virus (CHIKV) and Dengue virus (DENV) and its concomitant circulation in South Indian population. 2015; , 5.

18. Londhey V, Agrawal S, Vaidya N, Kini S, Shastri JS, Sunil S. Dengue and Chikungunya Virus Co-infections: The Inside Story. J Assoc Physicians India 2016 Mar;64(3):36-40.

19. Galate LB, Agrawal SR, Shastri JS, Londhey V. Chikungunya Fever Among Patients with Acute Febrile Illness Attending a Tertiary Care Hospital in Mumbai. J Lab Physicians 2016 Jul-Dec;8(2):85-89.

20. Nimmannitya S, Mansuwan P. Comparative clinical and laboratory findings in confirmed dengue and chikungunya infections. Bull World Health Organ 1966;35(1):42-43.

21. Halstead SB, Nimmannitya S, Margiotta MR. Dengue d chikungunya virus infection in man in Thailand, 1962-1964. II. Observations on disease in outpatients. Am J Trop Med Hyg 1969;18(6):972-983.

22. Laoprasopwattana K, Kaewjungwad L, Jarumanokul R, Geater A. Differential diagnosis of chikungunya, dengue viral infection and other acute febrile illnesses in children. Pediatr Infect Dis J 2012;31(5):459-463.

23. Khai Ming C, Thain S, Thaung U, U T, Myint KS, Swe T, et al. Clinical and laboratory studies on haemorrhagic fever in Burma, 1970-72. Bull World Health Organ 1974;51(3):227-235.

24. Mya Myat Ngwe Tun, Kyaw Zin Thant, Inoue S, Nabeshima T, Aoki K, Aung Kyaw Kyaw, et al. Detection of East/Central/South African genotype of Chikungunya virus in Myanmar, 2010. Emerging Infectious Diseases 2014;20(8):1378-1381.

25. Do QH, Vu TQH, Huynh TKL, Dinh QT, Deubel V. Dengue haemorrhagic fever in the south of Vietnam during 1975-1992 and its control strategy. Tropical Medicine 1994;36(4):187-201.

26. Nayar SK, Noridah O, Paranthaman V, Ranjit K, Norizah I, Chem YK, et al. Co-infection of dengue virus and Chikungunya virus in two patients with acute febrile illness. Med J Malaysia 2007;62(4):335-336.

27. Ooi MK, Gan HM, Rohani A, Syed Hassan S. First Complete Genome Sequence of a Chikungunya Virus Strain Isolated from a Patient Diagnosed with Dengue Virus Infection in Malaysia. Genome Announc 2016 Aug 25;4(4):10.1128/genomeA.00876-16.

28. Hapuarachchi HAC, Bandara, K. B. A. T., Hapugoda MD, Williams S, Abeyewickreme W. Laboratory confirmation of dengue and chikungunya co-infection. Ceylon Med J 2008;53(3):104-105.

29. Kularatne SAM, Gihan MC, Weerasinghe SC, Gunasena S. Concurrent outbreaks of Chikungunya and Dengue fever in Kandy, Sri Lanka, 2006-07: a comparative analysis of clinical and laboratory features. Postgrad Med J 2009;85(1005):342-346.

30. Ratsitorahina M, Harisoa J, Ratovonjato J, Biacabe S, Reynes JM, Zeller H, et al. Outbreak of dengue and chikungunya fevers, Toamasina, Madagascar, 2006. Emerging Infectious Diseases 2008;14(7):1135-1137.

31. Panning M, Grywna K, Esbroeck Mv, Emmerich P, Drosten C. Chikungunya fever in travelers returning to Europe from the Indian Ocean Region, 2006. Emerging Infectious Diseases 2008;14(3):416-422.

32. Leroy EM, Nkoghe D, Ollomo B, NzeNkogue C, Becquart P, Grard G, et al. Concurrent chikungunya and dengue virus infections during simultaneous outbreaks, Gabon, 2007. Emerging Infectious Diseases 2009;15(4):591-593.

33. Nkoghe D, Kassa RFK, Bisvigou U, Caron M, Grard G, Leroy EM. No clinical or biological difference between Chikungunya and Dengue fever during the 2010 Gabonese outbreak. Infectious Disease Reports 2012;4(1):11-13.

34. Caron M, Paupy C, Grard G, Becquart P, Mombo I, Nso BBB, et al. Recent introduction and rapid dissemination of Chikungunya virus and dengue virus serotype 2 associated with human and mosquito coinfections in Gabon, Central Africa. Clinical Infectious Diseases 2012;55(6):e45-e53.

35. Schilling S, Emmerich P, Gunther S, SchmidtChanasit J. Dengue and Chikungunya virus co-infection in a German traveller. Journal of Clinical Virology 2009;45(2):163-164.

36. Yoosuf AA, Shiham I, Mohamed AJ, Ali G, Luna JM, Pandav R, et al. First report of chikungunya from the Maldives. Trans R Soc Trop Med Hyg 2009;103(2):192-196.

37. Chang ShuFen, Su ChienLing, Shu PeiYun, Yang ChengFen, Liao TsaiLing, Cheng ChiaHsin, et al. Concurrent isolation of chikungunya virus and dengue virus from a patient with coinfection resulting from a trip to Singapore. J Clin Microbiol 2010;48(12):4586-4589.

38. Yang CF, Su CL, Hsu TC, Chang SF, Lin CC, Huang JC, et al. Imported Chikungunya Virus Strains, Taiwan, 2006-2014. Emerg Infect Dis 2016 Nov;22(11):1981-1984.

39. Parreira R, Centeno-Lima S, Lopes A, Portugal-Calisto D, Constantino A, Nina J. Dengue virus serotype 4 and chikungunya virus coinfection in a traveller returning from Luanda, Angola, January 2014. Euro Surveill 2014 Mar 13;19(10):20730.

40. Omarjee R, Prat CM, Flusin O, Boucau S, Tenebray B, Merle O, et al. Importance of case definition to monitor ongoing outbreak of chikungunya virus on a background of actively circulating dengue virus, St Martin, December 2013 TO January 2014. Eurosurveillance 2014;19(13).

41. Septfons A, Noël H, Leparc-Goffart I, Giron S, Delisle E, Chappert JL, et al. Surveillance du chikungunya et de la dengue en France métropolitaine, 2014. Bull Epidémiol Hebd. 2015;(13-14):204-11.

42. Diaz Y, Carrera JP, Cerezo L, Arauz D, Guerra I, Cisneros J, et al. Chikungunya virus infection: first detection of imported and autochthonous cases in Panama. Am J Trop Med Hyg 2015;92(3):482-485.

43. Campo Carey AR, Martinez Duran ME, Benavides Ocampo M, Constanza Cuellar N. Chikungunya outbreak in the municipality of San Juan Nepomuceno, Bolivar, Colombia, 2014. Informe Quincenal - Epidemiologico Nacional 2015;20(1):2-20.

44. Rosso F, Pacheco R, Rodriguez S, Bautista D. Co-infection by Chikungunya virus (CHIK-V) and dengue virus (DEN-V) during a recent outbreak in Cali, Colombia: Report of a fatal case. Rev Chilena Infectol 2016 Aug;33(4):464-467.

45. Calvo EP, Coronel-Ruiz C, Velazco S, Velandia-Romero M, Castellanos JE. Diagnóstico diferencial de dengue y chikungunya en pacientes pediátricos^ies; Dengue and Chikungunya differential diagnosis in pediatric patients^ien. Biomédica (Bogotá) 2016 08;36:35-43.

46. Shiferaw B, Lam P, Tuthill S, Choudhry H, Syed S, Ahmed S, et al. The Chikungunya Epidemic: A look at five cases. IDCases 2015 Sep 5;2(4):89-91.

47. Gosciminski M, Bandy U, Brady DS. Travel Associated Cases of Chikungunya Fever, Rhode Island, 2014. R I Med J (2013) 2015 Sep 1;98(9):47-49.

48. Millman AJ, Esposito DH, Biggs HM, Decenteceo M, Klevos A, Hunsperger E, et al. Chikungunya and Dengue Virus Infections Among United States Community Service Volunteers Returning from the Dominican Republic, 2014. Am J Trop Med Hyg 2016 Jun 1;94(6):1336-1341.

49. Pandey BD, Neupane B, Pandey K, Tun MM, Morita K. Detection of Chikungunya Virus in Nepal. Am J Trop Med Hyg 2015 Oct;93(4):697-700.

50. Sharp TM, Ryff KR, Alvarado L, Shieh WJ, Zaki SR, Margolis HS, et al. Surveillance for Chikungunya and Dengue During the First Year of Chikungunya Virus Circulation in Puerto Rico. J Infect Dis 2016 Dec 15;214(suppl 5):S475-S481.

51. Bandeira AC, Campos GS, Rocha VF, Souza BS, Soares MB, Oliveira AA, et al. Prolonged shedding of Chikungunya virus in semen and urine: A new perspective for diagnosis and implications for transmission. IDCases 2016 Nov 4;6:100-103.

52. Brooks JB, Ruiz CA, Fragoso YD. Acute illness with neurological findings caused by coinfection of dengue and chikungunya viruses in a Brazilian patient. J Infect Public Health 2016 Sep 5.

53. Bandeira AC, Campos GS, Sardi SI, Rocha VF, Rocha GC. Neonatal encephalitis due to Chikungunya vertical transmission: First report in Brazil. IDCases 2016 Jul 25;5:57-59.

54. Edwards T, Signor LD, Williams C, Donis E, Cuevas LE, Adams ER. Co-infections with Chikungunya and Dengue Viruses, Guatemala, 2015. Emerg Infect Dis 2016 Nov;22(11):2003-2005.

55. Fernandez-Garcia MD, Bangert M, de Ory F, Potente A, Hernandez L, Lasala F, et al. Chikungunya virus infections among travellers returning to Spain, 2008 to 2014. Euro Surveill 2016 Sep 8;21(36):10.2807/1560-7917.ES.2016.21.36.30336.

56. Bocanegra C, Anton A, Sulleiro E, Pou D, Salvador F, Roure S, et al. Imported cases of Chikungunya in Barcelona in relation to the current American outbreak. J Travel Med 2016 Mar 16;23(3):10.1093/jtm/tav033. Print 2016 Mar.

57. Phommanivong V, Kanda S, Shimono T, Lamaningao P, Darcy AW, Mishima N, et al. Co-circulation of the dengue with chikungunya virus during the 2013 outbreak in the southern part of Lao PDR. Trop Med Health 2016 Aug 4;44:24-016-0020-y. eCollection 2016.

58. Therrien C, Jourdan G, Holloway K, Tremblay C, Drebot MA. First Imported Case of Chikungunya Virus Infection in a Travelling Canadian Returning from the Caribbean. Can J Infect Dis Med Microbiol 2016;2016:2980297.

59. Cigarroa-Toledo N, Blitvich BJ, Cetina-Trejo RC, Talavera-Aguilar LG, Baak-Baak CM, Torres-Chable OM, et al. Chikungunya Virus in Febrile Humans and Aedes aegypti Mosquitoes, Yucatan, Mexico. Emerg Infect Dis 2016 Oct;22(10):1804-1807.

60. Balmaseda A, Gordon A, Gresh L, Ojeda S, Saborio S, Tellez Y, et al. Clinical Attack Rate of Chikungunya in a Cohort of Nicaraguan Children. Am J Trop Med Hyg 2016 Feb;94(2):397-399.

61. Marion C. Robinson. An epidemic of virus disease in Southern Province, Tanganyika Territory, in 1952-53, I. Clinical Features. Transactions of the Royal Society of Tropical Medicine and Hygiene 1955;49(1):28.

62. Thiruvengadam KV, Rao AR, Pavri KM, Rao TR. Haemagglutination inhibition tests on paired sera collected from cases of dengue-like illness in Madras city. Indian J Med Res 1965;53(8):702-706.

63. Hermon YE. Virological investigations of Arbovirus infections in Ceylon, with special reference to the recent Chikungunya fever epidemic. Ceylon Med J 1967;12(2):81-92.

64. Geser A, Henderson BE, Christensen S. A multipurpose serological survey in Kenya. 2. Results of arbovirus serological tests. Bull World Health Organ 1970;43(4):539-552.

65. Henderson BE, Kirya GB, Hewitt LE. Serological survey for arboviruses in Uganda, 1967-69. Bull World Health Organ 1970;42(5):797-805.

66. Ibrahim SH, Darwish MA, Wahdan MH, el-Ghoroury AA. Serologic survey of Kuwait population for evidence of group A arbovirus infection. J Egypt Public Health Assoc 1973;48(5):308-324.

67. Moore DL, Reddy S, Akinkugbe FM, Lee VH, DavidWest TS, Causey OR, et al. An epidemic of chikungunya fever at Ibadan, Nigeria, 1969. Ann Trop Med Parasitol 1974;68(1):59-68.

68. MuyembeTamfum JJ, Peyrefitte CN, Yogolelo R, Basisya EM, Koyange D, Pukuta E, et al. Epidemics of Chikungunya virus in 1999 and 2000 in the Democratic Republic of Congo. Med Trop 2003;63(6):637-638.

69. Pastorino B, MuyembeTamfum JJ, Bessaud M, Tock F, Tolou H, Durand JP, et al. Epidemic resurgence of Chikungunya virus in Democratic Republic of the Congo: identification of a new Central African strain. J Med Virol 2004;74(2):277-282.

70. Parola P, Lamballerie Xd, Jourdan J, Rovery C, Vaillant V, Minodier P, et al. Novel chikungunya virus variant in travelers returning from Indian Ocean islands. Emerging Infectious Diseases 2006;12(10):1493-1499.

71. Ramful D, Carbonnier M, Pasquet M, Bouhmani B, Ghazouani J, Noormahomed T, et al. Mother-to-child transmission of chikungunya virus infection. Pediatr Infect Dis J 2007;26(9):811-815.

72. Peyrefitte CN, Rousset D, Pastorino BAM, Pouillot R, Bessaud M, Tock F, et al. Chikungunya virus, Cameroon, 2006. Emerging Infectious Diseases 2007;13(5):768-771.

73. Bomin Al, Hebert JC, Marty P, Delaunay P. Confirmed chikungunya in children in Mayotte. Description of 50 patients hospitalized from February to June 2006. Revue Medecine Tropicale 2008;68(5):491-495.

74. Lemant J, Boisson V, Winer A, Thibault L, Andre H, Tixier F, et al. Serious acute Chikungunya virus infection requiring intensive care during the Reunion Island outbreak in 2005-2006. Crit Care Med 2008;36(9):2536-2541.

75. Sissoko D, Malvy D, Giry C, Delmas G, Paquet C, Gabrie P, et al. Outbreak of Chikungunya fever in Mayotte, Comoros archipelago, 2005-2006. Trans R Soc Trop Med Hyg 2008;102(8):780-786.

76. Ezzedine K, Cazanave C, Pistone T, Receveur MC, Neau D, Ragnaud JM, et al. Dual infection by chikungunya virus and other imported infectious agent in a traveller returning from India. Travel Medicine and Infectious Disease 2008;6(3):152-154.

77. SanchezSeco MP, Negredo AI, Puente S, Pinazo MJ, Shuffenecker I, Tenorio A, et al. Microbiological diagnosis of chikungunya virus in Spain (2006-2007): case detection in travelers. Enferm Infecc Microbiol Clin 2009;27(8):457-461.

78. Jentes ES, Robinson J, Johnson BW, Conde I, Sakouvougui Y, Iverson J, et al. Acute arboviral infections in Guinea, West Africa, 2006. Am J Trop Med Hyg 2010;83(2):388-394.

79. Kee ACL, Yang S, Tambyah P. Atypical Chikungunya virus infections in immunocompromised patients. Emerging Infectious Diseases 2010;16(6):1038-1040.

80. Collao X, Negredo AI, Cano J, Tenorio A, Ory Fd, Benito A, et al. Different lineages of Chikungunya virus in Equatorial Guinea in 2002 and 2006. Am J Trop Med Hyg 2010;82(3):505-507.

81. Chusri S, Siripaitoon P, Silpapojakul K. Chikungunya outbreak in Southern Thailand, December 2008 to October 2009. J Infect Dis Antimicrobial Agents 2011;28(1):25-34.

82. Nkoghe D, Kassa RF, Caron M, Grard G, Mombo I, Bikié B, et al. Clinical forms of Chikungunya in Gabon, 2010. PLoS Neglected Tropical Diseases 2012;6(2).

83. Lin J, Chen RW, Hazan A, Weiss M. Chikungunya Virus Infection Manifesting as Intermediate Uveitis. Ocul Immunol Inflamm 2016 Dec 23:1-3.

84. Hertz JT, Munishi OM, Ooi EngEong, Howe ShiQin, Lim WenYan, Chow A, et al. Chikungunya and dengue fever among hospitalized febrile patients in northern Tanzania. Am J Trop Med Hyg 2012;86(1):171-177.

85. Thenmozhi V, Paramasivan R, Samuel PP, Kamaraj T, Balaji T, Dhananjeyan KJ, et al. Dual infection in human by Japanese encephalitis virus & chikungunya virus in Alappuzha district, Kerala, India. Indian J Med Res 2013;138(3):362-363.

86. Ansumana R, Jacobsen KH, Leski TA, Covington AL, Bangura U, Hodges MH, et al. Reemergence of chikungunya virus in Bo, Sierra Leone. Emerging Infectious Diseases 2013;19(7):1108-1110.

87. Baba M, Logue CH, Oderinde B, Abdulmaleek H, Williams J, Lewis J, et al. Evidence of arbovirus co-infection in suspected febrile malaria and typhoid patients in Nigeria. Journal of Infection in Developing Countries 2013;7(1):51-59.

88. Chipwaza B, Mugasa JP, Selemani M, Amuri M, Mosha F, Ngatunga SD, et al. Dengue and Chikungunya fever among viral diseases in outpatient febrile children in Kilosa District Hospital, Tanzania. PLoS Neglected Tropical Diseases; 2014 3335;8(11).

89. Bacci A, Marchi S, Fievet N, Massougbodji A, Perrin RX, Chippaux JP, et al. High Seroprevalence of Chikungunya Virus Antibodies Among Pregnant Women Living in an Urban Area in Benin, West Africa. Am J Trop Med Hyg 2015; 92(6): 1133–1136.

90. Raut CG, Rao NM, Sinha DP, Hanumaiah H, Manjunatha MJ. Chikungunya, dengue, and malaria co-infection after travel to Nigeria, India. Emerg Infect Dis 2015 May;21(5):907-909.

91. Dalla Gasperina D, Balsamo ML, Garavaglia SD, Rovida F, Baldanti F, Grossi PA. Chikungunya infection in a human immunodeficiency virus-infected kidney transplant recipient returning to Italy from the Dominican Republic. Transpl Infect Dis 2015 Dec;17(6):876-879.

92. Betancur JF, Navarro EP, Echeverry A, Moncada PA, Canas CA, Tobon GJ. Hyperferritinemic syndrome: Still's disease and catastrophic antiphospholipid syndrome triggered by fulminant Chikungunya infection: a case report of two patients. Clin Rheumatol 2015 Nov;34(11):1989-1992.

93. Villamil-Gomez WE, Rodriguez-Morales AJ, Uribe-Garcia AM, Gonzalez-Arismendy E, Castellanos JE, Calvo EP, et al. Zika, dengue, and chikungunya co-infection in a pregnant woman from Colombia. Int J Infect Dis 2016 Oct;51:135-138.

94. Baudin M, Jumaa AM, Jomma HJ, Karsany MS, Bucht G, Naslund J, et al. Association of Rift Valley fever virus infection with miscarriage in Sudanese women: a cross-sectional study. Lancet Glob Health 2016 Nov;4(11):864-871.

95. Waggoner JJ, Gresh L, Vargas MJ, Ballesteros G, Tellez Y, Soda KJ, et al. Viremia and Clinical Presentation in Nicaraguan Patients Infected With Zika Virus, Chikungunya Virus, and Dengue Virus. Clin Infect Dis 2016 Dec 15;63(12):1584-1590.

96. Cabral-Castro MJ, Cavalcanti MG, Peralta RH, Peralta JM. Molecular and serological techniques to detect co-circulation of DENV, ZIKV and CHIKV in suspected dengue-like syndrome patients. J Clin Virol 2016 Sep;82:108-111.

97. Calvo EP, Sanchez-Quete F, Duran S, Sandoval I, Castellanos JE. Easy and inexpensive molecular detection of dengue, chikungunya and zika viruses in febrile patients. Acta Trop 2016 Nov;163:32-37.

98. Nhan TX, Bonnieux E, Rovery C, De Pina JJ, Musso D. Fatal leptospirosis and chikungunya co-infection: Do not forget leptospirosis during chikungunya outbreaks. IDCases 2016 Jun 22;5:12-14.

99. Sardi SI, Somasekar S, Naccache SN, Bandeira AC, Tauro LB, Campos GS, et al. Coinfections of Zika and Chikungunya Viruses in Bahia, Brazil, Identified by Metagenomic Next-Generation Sequencing. J Clin Microbiol 2016 Sep;54(9):2348-2353.

100. Mercado M, Acosta-Reyes J, Parra E, Pardo L, Rico A, Campo A, et al. Clinical and histopathological features of fatal cases with dengue and chikungunya virus co-infection in Colombia, 2014 to 2015. Euro Surveill 2016 Jun 2;21(22):10.2807/1560-7917

101. Waggoner JJ, Gresh L, Mohamed-Hadley A, Ballesteros G, Davila MJ, Tellez Y, et al. Single-Reaction Multiplex Reverse Transcription PCR for Detection of Zika, Chikungunya, and Dengue Viruses. Emerg Infect Dis 2016 Jul;22(7):1295-1297.

102. Ghiglione Y, Ruiz MJ, Salido J, Trifone C, Sued O, Martin Y, et al. Modification of the HIV-specific CD8+ T-cell response in an HIV elite controller after chikungunya virus infection. AIDS 2016 Jul 31;30(12):1905-1911.

103. Mendez-Dominguez N, Achach-Asaf JA, Basso-Garcia LM, Quinones-Pacheco YB, Gomez-Carro S. Septic shock secondary to non-congenital chikungunya fever in a young infant: A clinical case. Rev Chil Pediatr 2016 Mar-Apr;87(2):143-147.

104. Sow A, Loucoubar C, Diallo D, Faye O, Ndiaye Y, Senghor CS, et al. Concurrent malaria and arbovirus infections in Kedougou, southeastern Senegal. Malar J 2016 Jan 28;15:47-016-1100-5.

105. Villamil-Gomez WE, Gonzalez-Camargo O, Rodriguez-Ayubi J, Zapata-Serpa D, Rodriguez-Morales AJ. Dengue, chikungunya and Zika co-infection in a patient from Colombia. J Infect Public Health 2016 Sep-Oct;9(5):684-686.

106. Ayorinde AF, Oyeyiga AM, Nosegbe NO, Folarin OA. A survey of malaria and some arboviral infections among suspected febrile patients visiting a health centre in Simawa, Ogun State, Nigeria. J Infect Public Health 2016 Jan-Feb;9(1):52-59.

107. Zambrano H, Waggoner JJ, Almeida C, Rivera L, Benjamin JQ, Pinsky BA. Case report: Zika virus and chikungunya virus coinfections: A series of three cases from a single center in Ecuador. Am J Trop Med Hyg 2016;95(4):894-896.

108. Muñoz CM, Castillo JO, Salas D, Valderrama MA, Rangel CT, Vargas HP, et al. Atypical mucocutaneous manifestations in neonates and infants with chikungunya fever in the municipalities of Cúcuta, Los Patios and Villa del Rosario, Norte de Santander, Colombia, 2014. Biomedica 2016;36(3):368-377.

109. Mogami R, de Almeida Vieira A, Junqueira Filho EA, Lopes AJ. Chikungunya fever outbreak in Rio de Janeiro, Brazil: Ultrasonographic aspects of musculoskeletal complications. J Clin Ultrasound 2017 Jan;45(1):43-44.
