# Supplementary material for: A scoping review of published literature on chikungunya virus
Source: PLoS One. 2018 Nov 29;13(11):e0207554. doi: 10.1371/journal.pone.0207554 (PMC6264817; doi:10.1371/journal.pone.0207554)
Supplement: S6 Table — (DOCX) [file pone.0207554.s009.docx]

| Reference  **S6 Table: Studies on materno-fetal transmission with chikungunya infection** | Country | Materno-fetal or post natal infection | Pregnant mothers | Neonates | Comments |
| --- | --- | --- | --- | --- | --- |
| *Outbreak reports* | | | | | |
| [1] | Réunion Island | Mother-to-child transmission or post-natal infection |  | 27 serious infections have been notified in newborns | Eurosurveillance report - acquired either by mother-to-baby transmission or by mosquito bites in newborns aged > nine days |
| [2] | Comoros island | Mother-to-child transmission | The mothers of all six children had acute chikungunya infection within the 48 hours before delivery | Six newborns who showed symptoms of acute infection, and presented with a meningoencephalitis picture, within five days of birth | Eurosurveillance report - acquired either by mother-to-baby transmission or by mosquito bites in newborns aged > nine days |
| [3] | French Territories | Mother-to-child transmission | 44 pregnant mothers infected | 44 materno-neonatal infections | Eurosurveillance report |
| [4] | Réunion Island | Mother-to-child transmission |  | Six cases occurred in newborns, and mother-to-child transmission is strongly suspected in these cases | Eurosurveillance report |
| [5] | Kerala, India | Mother-to-child transmission |  | Unexpected infection in neonates | Noticed an increased incidence of meconium stained amniotic fluid and meconium aspiration syndrome associated with this fever in mothers |
| [6] | Dominican Republic | Mother-to-child transmission | 84 pregnant mothers | 49 neonates infected /84 pregnant mothers |  |
| [7] | French overseas territories of the Americas | Mother-to-child transmission | 2 pregnant mothers | 2 cases of materno-fetal transmission - 1 case with severe disease |  |
| [8] | Réunion Island | Mother-to-child transmission | 43 confirmed materno-neonatal transmission in the perinatal period | 43 | 43 confirmed materno-neonatal transmission in the perinatal period. All neonates from the 43 mothers experienced symptoms |
| [9] | Réunion Island | Mother-to-child transmission |  | 44 under 10 days of age | Mother-to-child transmission cases reported |
| [10] | Réunion Island-Mayotte IREU | Mother-to-child transmission |  | 44 neonates |  |
| [11] | Réunion Island | Mother-to-child transmission |  | 44 neonates |  |
| [12] | Réunion Island | Mother-to-child transmission | 739/7504 [9.8%]. All near-term deliveries [median length of gestation: 38 wk, range 35–40 wk]. C-section had no influence on mother-to-child viral transmission, either for intrapartum infections | 19 cases of vertical transmission out of 39 women with intrapartum viremia, prevalence rate 0.25%, vertical transmission rate 48.7%. The median onset of neonatal disease [defining the incubation time] was 4 days | Enrolled all parturient women and their offspring admitted at the maternity department of Groupe Hospitalier |
| [13] | Réunion Island | Mother-to-child transmission | 2905 pregnant mothes | 70 neonates |  |
| [14] | Réunion Island | Mother-to-child transmission |  | 55 cases of materno-neonatal transmission were reported in infants less than 10 days of age; 41 of these cases were confirmed and included 10 infants with meningoencephalitis and 1 death |  |
| [15] |  | Mother-to-child transmission | 36 pregnant mothers | Thirty-eight neonates born between March 2005 and April 2006 were included in this study. One pair of twins | Retrospective descriptive study conducted by 5 neonatal medicine departments in public or private hospitals |
| [16] | France | Mother-to-child transmission | 7 pregnant mothners | 3 aborted fetuses tested positive for CHIKV | First three cases of maternal-fetal transmission of the Chikungunya virus [CHIKV] before 16 weeks' gestational age. |
| [17] | Réunion Island | Mother-to-child transmission |  |  |  |
| [18] | Réunion Island | Mother-to-child transmission | Cases > From June 2005 through the end of January 2006, 84 pregnant women had acute chikungunya infections during pregnancy | 10 newborns infected with severe illness. 74 neonates asymptomatic when mothers were infected distant to delivery | In 88% of these cases [n = 74] — all involving infections relatively distant from delivery — the newborns appeared asymptomatic. Conversely, 10 newborns had severe attacks [4 with meningoencephalitis and 3 with intravascular coagulations] after birth and required prolonged neonatal hospitalization [6 in the neonatal intensive care unit with intubation and assisted ventilation] |
| [19] | Réunion Island | Mother-to-child transmission | 6 – Mothers infected within 48 hours of delivery | 6 newborn cases born with infection |  |
| [20] | Brazil | Mother-to-child transmission | 1 infected mother | 1 infected neonate | First case of Chikungunya encephalitis acquired in perinatal period |
| [21] | Thailand | Mother-to-child transmission | 5 pregnant mothers | 0 neonates infected | Community-based post-epidemic seroprevalence study was conducted in parturient women |
| *Case study reports* | | | | | |
| [22] | Benin, West Africa |  | High seroprevalence of chikungunya virus antibodies among pregnant women living in an urban area in Benin | No cases of acute/recent CHIKV infection were detected at delivery | No chance of mother to-child transmission of CHIKV in study group |
| [23] | India |  |  | 3 neonates with postnatally acquired chikungunya |  |
| [24] | India | Mother-to-child transmission | 1 mother with fever a week prior to delivery. Confirmed for infection after delivery | 1 neonate infected -born to mother with fever a week before delivery. Neonate confirmed as case | Neonate born to a mother with fever for a week prior to delivery suggested the possibility of neonatal chikungunya, which was confirmed by positive IgM antibodies to chikungunya in both the mother and baby |
| [25] | India | Mother-to-child transmission | 1 mother infected | 1 baby infected |  |
| [26] | India | Mother-to-child transmission | 1 mother infected | A case of asymptomatic infection in a 20 day old infant |  |
| [27] | Sri Lanka | Mother-to-child transmission | 48 pregnant women with confirmed infection; 2 women post- partum with infected neonates | 7 neonates infected from 32 births to infected mothers. Evidence of perinatal infection in 2 neonates | Prospective descriptive study of women attending maternity clinics |
| [28] | India | Mother-to-child transmission | 2 mothers infected during pregnancy | 2 infected neonates |  |
| [29] | India | Mother-to-child transmission | 1 mother confirmed for CHIKV with infection 3 days prior to delivery | 1 neonate infected at birth with mother diagnosed 3 days prior to delivery |  |
| [30] | India | Mother-to-child transmission |  |  | Neurodevelopmental follow-up of neonates with vertically transmitted Chikungunya fever up to 3 years of age |
| [31] | India | Mother-to-child transmission | 21 infected | 21 infected | Eighty pairs of mother-cord sera were examined for presence of HI antibodies to CHIKV. 59/80 pairs of sera were not positive for CHIKV |
| [32] | Réunion Island | Mother-to-child transmission |  | 19 neonates infected | Report the first case of chikungunya neonatal infection and the 5-years outcome of the 18 neonates hospitalized in neonatal intensive care unit between June 2005 and March 2006 |
| [33] | India | Mother-to-child transmission |  | 1 neonate infected |  |
| [34] | Réunion Island | Mother-to-child transmission |  | 1 neonate infected | Materno-fetal syndrome associated with Bernard-Soulier syndrome in the neonate |
| [35] | Réunion Island | Mother-to-child transmission | 4 CHIK infected pregnant mothers | 1 newborn infected on the third day of life. The mother had had chikungunya symptoms the day before delivery. The other 3 neonates remained asymptomatic and had no detectable IgM against chikungunya virus. Of 624 placentas examined from women found to be infected during pregnancy, only the placenta from the case of mother-to-child transmission had histologic signs compatible with viral infection | Recruited all pregnant women [with or without symptoms of chikungunya infection] who received care at 1 of the 6 main maternity units in Réunion to determine the consequences of chikungunya infection on pregnancy outcomes |
| [36] | Réunion Island-Mayotte | Mother-to-child transmission |  | 44 newborns aged < 10 days infected |  |
| [37] | India |  |  | Postnatally acquired CHIKV case |  |
| [38] | India | Mother-to-child transmission | 1 infected mother | Vertically transmitted chikungunya infection in heterozygous twin neonates presenting as seizures, encephalopathy, midfacial hyperpigmentation, anemia, and thrombocytopenia |  |
| [39] | Several countries in South America | Mother-to-child transmission | 191 in El Salvador, All women were in the third trimester of pregnancy [mean gestational age 38.2 weeks, range 35–40 weeks]. Mothers with documented [20 patients] or suspected [17 patients] clinical CHIKF, were evaluated in Neiva, Colombia. In Dominican Republic, 205 pregnant women with symptoms of CHIKF, classified as suspected cases were evaluated | A total of 53 newborns developed a congenital infection rate 27.7%]. Premature delivery occurred in 17 cases [8.9%], but no miscarriages were observed. Thirty-seven symptomatic neonates [24 male, 13 female] born to mothers with documented [20 patients] or suspected [17 patients] clinical CHIKF, were in Colombia. In Dominican Republic, a total of 99 newborns developed symptoms suggestive of the condition. The vertical transmission rate was 48.29% [99 symptomatic neonatal infections out of 205 mothers]. Caesarean sections were performed in 59 of the 99 symptomatic mothers [74.6%]. The incidence of congenital disease in newborns was statistically unrelated to cesarean delivery. Vertical transmission rates in the present series [27.2% to 48.29%] | Prospective clinical and epidemiological data collected from all symptomatic pregnant women and newborns with a confirmed diagnosis of CHIKF |
| [40] | Colombia |  | 1 pregnant mother with triple co-infection of CHIKV, DENV and ZIKV |  | Pregnancy outcome unknown as paper published during case pregnancy |
| [41] | Sudan | Mother-to-child transmission | 31 -normal pregnancy -20/31, miscarriage - 8/31, preterm delivery - 3/31 | 20 normal delivery babies and 3 preterm babies, Of the four women with preterm delivery, one was coinfected with Rift Valley fever virus and chikungunya virus, two others were positive for chikungunya virus only. Preterm delivery was associated with chikungunya virus infection | Retrieved clinical information and laboratory test results from the patients’ medical records and stored blood samples were analysed |
| [42] | Puerto Rico | Mother-to-child transmission | 10 pregnant cases; 7 with symptoms | 10 neonates |  |
| [43] | Brazil | Mother-to-child transmission | 2 pregnant cases | 2 infected neonates |  |
| [44] | Colombia |  | 1 co- infected mother with DENV and CHIKV | Two days after hospital admission, this patient gave birth to a live child with normal physical examination |  |
| [45] | Colombia | Mother-to-child transmission | 1 pregnant mother | a case of congenital CHIK and a case of neonatal CHIK infection |  |
| [46] | Colombia | Mother-to-child transmission | Retrospective case series of 7 pregnant women with confirmed CHIKV who delivered 8 neonates | All 8 newborns [1 set of twins] were confirmed to be infected with CHIKV | Of the total cohort of neonates, 3 died, including the twins. Two neonates who died presented necrotizing enterocolitis and sepsis. No congenital malformations in any of the neonates |
| [47] | India | Mother-to-child transmission | 1 infected mother | 12-day-old neonate infected |  |
| [48] | India | Mother-to-child transmission | Pregnant lady who had transient fever for two days in the third trimester | 1 foetal pericardial effusion and intrauterine growth restriction |  |
| [49] | Colombia | Mother-to-child transmission |  | 4 cases suspected of vertical transmission | Reviewed the cases of newly born and lactating mothers until December 2014 with a diagnosis of the chikungunya virus |

**References:**

1. Cordel H, Investigation Group. Chikungunya outbreak on Reunion: update. Euro Surveill 2006 Mar 2;11(3):E060302.3.

2. Paquet C, Quatresous I, Solet JL, Sissoko D, Renault P, Pierre V, et al. Chikungunya outbreak in Reunion: epidemiology and surveillance, 2005 to early January 2006. Euro Surveill 2006 Feb 2;11(2):E060202.3.

3. Ledrans M, Quatresous I, Renault P, Pierre V. Outbreak of chikungunya in the French Territories, 2006: lessons learned. Euro surveillance : bulletin européen sur les maladies transmissibles = European communicable disease bulletin 2007;12(9).

4. Quatresous I, Investigation Group. E-alert 27 January: Chikungunya outbreak in Reunion, a French overseas department. Euro Surveill 2006 Feb 2;11(2):E060202.1.

5. Nair PMC. Chikungunya in neonates. Indian Pediatr 2008;45(7):605.

6. Pimentel R, SkewesRamm R, Moya J. Chikungunya in the Dominican Republic: lessons learned in the first six months. Rev Panam Salud Publica/Pan Am J Public Health 2014;36(5):336-341.

7. Ledrans M, Cassadou S, Boucau S, HucAnais P, LeparcGoffart I, Prat C, et al. Emergence of chikungunya in the French overseas territories of the Americas: organization and results of epidemiological surveillance, April 2014. Bulletin Epidemiologique Hebdomadaire 2014(21/22):368-379.

8. Flahault A, Aumont G, Boisson V, Lamballerie Xd, Favier F, Fontenille D, et al. An interdisciplinary approach to controlling chikungunya outbreaks on French islands in the south-west Indian Ocean. (Special chikungunya.). Revue Medecine Tropicale 2011;72(Special):66-71.

9. Larrieu S, Balleydier E, Renault P, Baville M, Filleul L. Epidemiological surveillance du chikungunya on Reunion Island from 2005 to 2011. (Special chikungunya.) [French]. Revue Medecine Tropicale 2011;72(Special):38-42.

10. Ramful D, Samperiz S, Fritel X, Michault A, JaffarBandjee MC, Rollot O, et al. Antibody kinetics in infants exposed to Chikungunya virus infection during pregnancy reveals absence of congenital infection. J Infect Dis 2014;209(11):1726-1730.

11. Dominguez M, Economopoulou A, Sissoko D, Boisson V, Gauzere BA, Pierre V, et al. Atypical forms of chikungunya in epidemic period, Reunion Island, France, 2005-2006. (Special issue - What did we learn from the chikungunya outbreak in the Indian Ocean in 2005-2006?) [French]. Bulletin Epidemiologique Hebdomadaire 2008(38/40):349-352.

12. Gerardin P, Barau G, Michault A, Bintner M, Randrianaivo H, Choker G, et al. Multidisciplinary prospective study of mother-to-child chikungunya virus infections on the Island of La Reunion. PLoS Medicine 2008;5(3):e60.

13. Grivard P, Roux Kl, Laurent P, Fianu A, Perrau J, Gigan J, et al. Molecular and serological diagnosis of Chikungunya virus infection. Pathologie Biologie 2007;55(10):490-494.

14. Renault P, Solet JL, Sissoko D, Balleydier E, Larrieu S, Filleul L, et al. A major epidemic of chikungunya virus infection on Reunion Island, France, 2005-2006. Am J Trop Med Hyg 2007;77(4):727-731.

15. Ramful D, Carbonnier M, Pasquet M, Bouhmani B, Ghazouani J, Noormahomed T, et al. Mother-to-child transmission of chikungunya virus infection. Pediatr Infect Dis J 2007;26(9):811-815.

16. Touret Y, Randrianaivo H, Michault A, Schuffenecker I, Kauffmann E, Lenglet Y, et al. Early maternal-fetal transmission of the Chikungunya virus. Presse Medicale 2006;35(11(Cahier 1):1656-1658.

17. Bessaud M, Peyrefitte CN, Pastorino BAM, Tock F, Merle O, Colpart JJ, et al. Chikungunya virus strains, Reunion Island outbreak. Emerging Infectious Diseases 2006;12(10):1604-1606.

18. Robillard PY, Boumahni B, Gerardin P, Michault A, Fourmaintraux A, Schuffenecker I, et al. Vertical maternal-fetal transmission of chikungunya virus: ten cases on Reunion among 84 pregnant women. Presse Medicale 2006;35(5(Cahier 1):785-788.

19. Paquet C, Quatresous I, Solet JL, Sissoko D, Renault P, Pierre V, et al. Epidemiology of chikungunya virus infection on the Island of Reunion: situation on 8 January 2006. (Infection par le virus Chikungunya a l'Ile de la Reunion) [French]. Bulletin Epidemiologique Hebdomadaire 2006(hors serie):2-3.

20. Bandeira AC, Campos GS, Sardi SI, Rocha VF, Rocha GC. Neonatal encephalitis due to Chikungunya vertical transmission: First report in Brazil. IDCases 2016 Jul 25;5:57-59.

21. Laoprasopwattana K, Suntharasaj T, Petmanee P, Suddeaugrai O, Geater A. Chikungunya and dengue virus infections during pregnancy: seroprevalence, seroincidence and maternal-fetal transmission, southern Thailand, 2009-2010. Epidemiol Infect 2016 Jan;144(2):381-388.

22. Bacci A, Marchi S, Fievet N, Massougbodji A, Perrin RX, Chippaux JP, et al. High Seroprevalence of Chikungunya Virus Antibodies Among Pregnant Women Living in an Urban Area in Benin, West Africa. Am J Trop Med Hyg 2015; 92(6): 1133–1136.

23. Gupta D, Bose A, Rose W. Acquired Neonatal Chikungunya Encephalopathy. Indian J Pediatr 2015;82(11):1065-6

24. Kumar N, Gupta V, Thomas N. Brownie-nose: hyperpigmentation in neonatal chikungunya. Indian J Pediatr 2014;51(5):419.

25. Gopakumar H, Ramachandran S. Congenital chikungunya. J Clin Neonatol 2012 Jul;1(3):155-156.

26. Khandelwal K, Aara N, Ghiya BC, Bumb RA, Satoskar AR. Centro-facial pigmentation in asymptomatic congenital chikungunya viral infection. J Paediatr Child Health 2012 Jun;48(6):542-543.

27. Senanayake MP, Senanayake SM, Vidanage KK, Gunasena S, Lamabadusuriya SP. Vertical transmission in chikungunya infection. Ceylon Med J 2009 Jun;54(2):47-50.

28. Rao G, Khan YZ, Chitnis DS. Chikungunya infection in neonates. Indian Pediatr 2008 Mar;45(3):240-242.

29. Shrivastava A, Waqar Beg M, Gujrati C, Gopalan N, Rao PVL. Management of a vertically transmitted neonatal Chikungunya thrombocytopenia. Indian J Pediatr 2011;78(8):1008-1009.

30. Shenoy S, Pradeep GCM. Neurodevelopmental outcome of neonates with vertically transmitted chikungunya fever with encephalopathy. Indian Pediatr 2012;49(3):238-239.

31. Sarkar JK, Chakravarty SK, Ray SN. Transplacental transmission of haemagglutination inhibiting antibodies of pox and arboviruses. Indian J Med Res 1973;61(8):1153-1157.

32. Boumahni B, Bintner M. Five-year outcome of mother-to-child transmission of chikungunya virus. (Special chikungunya.) [French]. Revue Medecine Tropicale 2011;72(Special):94-96.

33. Shetty PK. Neonatal chikungunya - a case report. Pediatric OnCall 2011;8(12):80.

34. Boumahni B, Kaplan C, Clabe A, Randrianaivo H, Lanza F. Maternal-fetal chikungunya infection associated with Bernard-Soulier syndrome. (Neonatologie.) [French]. Archives de Pediatrie 2011;18(3):272-275.

35. Fritel X, Rollot O, Gerardin P, Gauzere BA, Bideault J, Lagarde L, et al. Chikungunya virus infection during pregnancy, Reunion, France, 2006. Emerging Infectious Diseases 2010;16(3):418-425.

36. Economopoulou A, Dominguez M, Helynck B, Sissoko D, Wichmann O, Quenel P, et al. Atypical Chikungunya virus infections: clinical manifestations, mortality and risk factors for severe disease during the 2005-2006 outbreak on Reunion. Epidemiol Infect 2009;137(4):534-541.

37. Peter R, Krishnan L, Anandraj V, Kuruvila S. Chikungunya in a newborn. Journal of Clinical Neonatology 2015 01 Apr 2015;4(2):145-146.

38. Karthiga V, Kommu PP, Krishnan L. Perinatal chikungunya in twins. J Pediatr Neurosci 2016 Jul-Sep;11(3):223-224.

39. Torres JR, Falleiros-Arlant LH, Duenas L, Pleitez-Navarrete J, Salgado DM, Castillo JB. Congenital and perinatal complications of chikungunya fever: a Latin American experience. Int J Infect Dis 2016 Oct;51:85-88.

40. Villamil-Gomez WE, Rodriguez-Morales AJ, Uribe-Garcia AM, Gonzalez-Arismendy E, Castellanos JE, Calvo EP, et al. Zika, dengue, and chikungunya co-infection in a pregnant woman from Colombia. Int J Infect Dis 2016 Oct;51:135-138.

41. Baudin M, Jumaa AM, Jomma HJ, Karsany MS, Bucht G, Naslund J, et al. Association of Rift Valley fever virus infection with miscarriage in Sudanese women: a cross-sectional study. Lancet Glob Health 2016 Nov;4(11):e864-e871.

42. Rodriguez-Nieves M, Garcia-Garcia I, Garcia-Fragoso L. Perinatally Acquired Chikungunya Infection: The Puerto Rico Experience. Pediatr Infect Dis J 2016 Oct;35(10):1163.

43. Lyra PP, Campos GS, Bandeira ID, Sardi SI, Costa LF, Santos FR, et al. Congenital Chikungunya Virus Infection after an Outbreak in Salvador, Bahia, Brazil. AJP Rep 2016 Jul;6(3):e299-300.

44. Mercado M, Acosta-Reyes J, Parra E, Pardo L, Rico A, Campo A, et al. Clinical and histopathological features of fatal cases with dengue and chikungunya virus co-infection in Colombia, 2014 to 2015. Euro Surveill 2016 Jun 2;21(22):10.2807/1560-7917.ES.2016.21.22.30244.

45. Alvarado-Socarras JL, Ocampo-Gonzalez M, Vargas-Soler JA, Rodriguez-Morales AJ, Franco-Paredes C. Congenital and Neonatal Chikungunya in Colombia. J Pediatric Infect Dis Soc 2016 Sep;5(3):e17-20.

46. Villamil-Gomez W, Alba-Silvera L, Menco-Ramos A, Gonzalez-Vergara A, Molinares-Palacios T, Barrios-Corrales M, et al. Congenital Chikungunya Virus Infection in Sincelejo, Colombia: A Case Series. J Trop Pediatr 2015 Oct;61(5):386-392.

47. Vasani R, Kanhere S, Chaudhari K, Phadke V, Mukherjee P, Gupta S, et al. Congenital Chikungunya--A Cause of Neonatal Hyperpigmentation. Pediatr Dermatol 2016 Mar-Apr;33(2):209-212.

48. Nigam A, Sharma S, Jain A, Gupta A, Prakash A. Vertical transmission of chikungunya manifesting as foetal pericardial effusion. Journal of Association of Physicians of India 2016;64:76-79.

49. Muñoz CM, Castillo JO, Salas D, Valderrama MA, Rangel CT, Vargas HP, et al. Atypical mucocutaneous manifestations in neonates and infants with chikungunya fever in the municipalities of Cúcuta, Los Patios and Villa del Rosario, Norte de Santander, Colombia, 2014. Biomedica 2016;36(3):368-377.
