# Supplementary material for: A scoping review of published literature on chikungunya virus
Source: PLoS One. 2018 Nov 29;13(11):e0207554. doi: 10.1371/journal.pone.0207554 (PMC6264817; doi:10.1371/journal.pone.0207554)
Supplement: S7 Table — (DOCX) [file pone.0207554.s010.docx]

| **Refid** | **Country** | **Publication year** | **Economic measure assessed** |
| --- | --- | --- | --- |
| [1] | India | 2013 | Loss of work due to long term morbidity |
| [2] | Indonesia | 2013 | Absenteeism at work due to CHIKV infection |
| [3] | India | 2009 | Treatment costs for private healthcare. Government offers free treatment |
| [4] | Italy | 2008 | Loss of gross proceeds during a CHIKV outbreak due to depletion of blood supply and reduced blood inventory |
| [5] | India | 2007 | Impact of CHIKV infection on patients with different income levels |
| [6] | Reunion Island | 2014 | Cost-benefit of control measures |
| [7] | Reunion Island | 2013 | Cost of treatment for post-CHIKV related long term arthralgias |
| [8] | India | 2013 | Costs related to work days lost, expenditures on medicines, costs of transportation, medical consults and diagnosis – Chik infection related |
| [9] | India | 2010 | Costs arising from treatment, transportation ,wages lost |
| [10] | India | 2009 | Costs per case, costs on measures to prevent and control chikungunya and dengue – mainly insecticides and personnel |
| [11] | India | 2012 | Average cost per admission of chikungunya patient |
| [12] | India | 2012 | Costs of treatment, food, loss of man days |
| [13] | Reunion Islands | 2012 | Economic impact due to loss in tourism |
| [14] | India | 2011 | Out-of-pocket expenses among patients during the course of chikungunya illness |
| [15] | Reunion Island | 2011 | Medical costs relating to consultation, hospitalization and drugs. Comparison of costs during epidemic and non-epidemic periods |
| [16] | Canada | 2010 | Cost effectiveness of whole blood PRT compared to other interventions used in Canada |
| [17] | India | 2010 | Estimating the burden of disease using DALYs and direct and indirect costs of chikungunya in India |
| [18] | India | 2009 | Costs for diagnosis, drugs and consultations, transportation, hospitalizations and loss of income |
| [19] | India | 2009 | Estimate of national burden due to CHIK epidemic of 2006. Burden of DALYs, productivity loss, income foregone, burden from long term sequelae |
| [20] | Fiji | 1980 | Cost of vector control interventions like habitat clearing, public education and insecticide spraying |
| [21] | Colombia | 2015 | Estimation of DALYs lost and outbreak costs for 2014 outbreak |
| [22] | Colombia | 2015 | DALYs lost |
| [23] | Colombia | 2015 | Comparison of chikungunya related DALYs in Colombia versus Latin America |

**References:**

1. Mohanty I, Dash M, Sahu S, Narasimham MV, Panda P, Padhi S. Seroprevalence of chikungunya in southern Odisha. J Family Med Prim Care 2013 Jan;2(1):33-36.

2. Kosasih H, de Mast Q, Widjaja S, Sudjana P, Antonjaya U, Ma'roef C, et al. Evidence for endemic chikungunya virus infections in Bandung, Indonesia. PLoS Negl Trop Dis 2013 Oct 24;7(10):e2483.

3. Nandha B, Krishnamoorthy K. Cost of illness due to Chikungunya during 2006 outbreak in a rural area in Tamil Nadu. Indian J Public Health 2009 Oct-Dec;53(4):209-213.

4. Liumbruno GM, Calteri D, Petropulacos K, Mattivi A, Po C, Macini P, et al. The Chikungunya epidemic in Italy and its repercussion on the blood system. Blood Transfus 2008 Oct;6(4):199-210.

5. Kumar CJ, Baboo CA, Krishnan BU, Kumar A, Joy S, Jose T, et al. The socioeconomic impact of the chikungunya viral epidemic in India. Open Med 2007;1(3):e150-2.

6. Thuilliez J, Bellia C, Dehecq JS, Reilhes O. Household-level expenditure on protective measures against mosquitoes on the island of La Réunion, France. PLoS Neglected Tropical Diseases 2014;8(1).

7. Schilte C, Staikovsky F, Couderc T, Madec Y, Carpentier F, Kassab S, et al. Chikungunya Virus-associated Long-term Arthralgia: A 36-month Prospective Longitudinal Study. PLoS Neglected Tropical Diseases 2013;7(3).

8. Vijayakumar K, George B, Anish TS, Rajasi RS, Teena MJ, Sujina CM. Economic impact of chikungunya epidemic: out-of-pocket health expenditures during the 2007 outbreak in Kerala, India. Southeast Asian J Trop Med Public Health 2013;44(1):54-61.

9. Dileep Mavalankar, Dipti Govil, Neha Trivedi, Vinubhai P. Prevalence of various symptoms and cost of treatment during the chikungunya epidemic in Ahmedabad, Gujarat, India, in 2006. (Special Issue: Cost and burden of dengue and chikungunya from the Americas to Asia.). Dengue Bulletin; 2010.34:46-53.

10. Murtola TM, Vasanb SS, Puwar TI, Dipti Govil, Fielde RW, Gong HongFei, et al. Preliminary estimate of immediate cost of chikungunya and dengue to Gujarat, India. (Special Issue: Cost and burden of dengue and chikungunya from the Americas to Asia.). Dengue Bulletin; 2010.34:32-39.

11. Kiran LJ, Jyothi CH, Narendranath S, Gokul CG, Shivashankaramurthy KG, Srinivas LD, et al. Prescribing patterns in clinically suspected chikungunya: a retrospective study. Journal of Pharmacy Research 2012;5(3):1704-1705.

12. Nagpal BN, Rekha Saxena, Aruna Srivastava, Neeru Singh, Ghosh SK, Sharma SK, et al. Retrospective study of chikungunya outbreak in urban areas of India. Indian J Med Res 2012;135(3):351-358.

13. Flahault A, Aumont G, Boisson V, Lamballerie Xd, Favier F, Fontenille D, et al. An interdisciplinary approach to controlling chikungunya outbreaks on French islands in the south-west Indian Ocean. (Special chikungunya.). Revue Medecine Tropicale 2011;72(Special):66-71.

14. Murhekar MV, Manickam P, Kumar RM, Ganesakumar SRB, Ramachandran V, Ramakrishnan R, et al. Treatment practices & laboratory investigations during Chikungunya outbreaks in South India. Indian J Med Res 2011;133(5):546-547.

15. Soumahoro MK, Boelle PY, Gauzere BA, Atsou K, Pelat C, Lambert B, et al. The chikungunya epidemic on La Reunion Island in 2005-2006: a cost-of-illness study. PLoS Neglected Tropical Diseases; 2011 1197;5(6)

16. Custer B, Agapova M, Martinez RH. The cost-effectiveness of pathogen reduction technology as assessed using a multiple risk reduction model. Transfusion 2010;50(11):2461-2473.

17. Seyler T, Hutin Y, Ramanchandran V, Ramakrishnan R, Manickam P, Murhekar M. Estimating the burden of disease and the economic cost attributable to chikungunya, Andhra Pradesh, India, 2005-2006. Trans R Soc Trop Med Hyg 2010;104(2):133-138.

18. Gopalan SS, Ashis Das. Household economic impact of an emerging disease in terms of catastrophic out-of-pocket health care expenditure and loss of productivity: investigation of an outbreak of chikungunya in Orissa, India. Journal of Vector Borne Diseases 2009;46(1):57-64.

19. Krishnamoorthy K, Harichandrakumar KT, Kumari AK, Das LK. Burden of chikungunya in India: estimates of disability adjusted life years (DALY) lost in 2006 epidemic. Journal of Vector Borne Diseases 2009;46(1):26-35.

20. Goettel MS, Toohey MK, Pillai JS. The urban mosquitoes of Suva, Fiji: seasonal incidence and evaluation of environmental sanitation and ULV spraying for their control. J Trop Med Hyg 1980 Aug;83(4):165-171.

21. Cardona-Ospina JA, Villamil-Gomez WE, Jimenez-Canizales CE, Castaneda-Hernandez DM, Rodriguez-Morales AJ. Estimating the burden of disease and the economic cost attributable to chikungunya, Colombia, 2014. Trans R Soc Trop Med Hyg 2015 Dec;109(12):793-802.

22. Cardona-Ospina JA, Diaz-Quijano FA, Rodriguez-Morales AJ. Burden of chikungunya in Latin American countries: estimates of disability-adjusted life-years (DALY) lost in the 2014 epidemic. Int J Infect Dis 2015 Sep;38:60-61.

23. Cardona-Ospina JA, Rodriguez-Morales AJ, Villamil-Gomez WE. The burden of Chikungunya in one coastal department of Colombia (Sucre): Estimates of the disability adjusted life years (DALY) lost in the 2014 epidemic. J Infect Public Health 2015 Nov-Dec;8(6):644-646.
